# Current Status of the Disease-Resistant Gene(s)/QTLs, and Strategies for Improvement in *Brassica juncea*

**DOI:** 10.3389/fpls.2021.617405

**Published:** 2021-03-03

**Authors:** Kaushal Pratap Singh, Preetesh Kumari, Pramod Kumar Rai

**Affiliations:** ^1^ICAR-Directorate of Rapeseed-Mustard Research, Bharatpur, India; ^2^Genetics Division, ICAR-Indian Agricultural Research Institute, New Delhi, India

**Keywords:** *B. juncea*, diseases, resistant sources, quantitative trait loci, improvement strategies

## Abstract

*Brassica juncea* is a major oilseed crop in tropical and subtropical countries, especially in south-east Asia like India, China, Bangladesh, and Pakistan. The widespread cultivation of genetically similar varieties tends to attract fungal pathogens which cause heavy yield losses in the absence of resistant sources. The conventional disease management techniques are often expensive, have limited efficacy, and cause additional harm to the environment. A substantial approach is to identify and use of resistance sources within the Brassica hosts and other non-hosts to ensure sustainable oilseed crop production. In the present review, we discuss six major fungal pathogens of *B. juncea*: Sclerotinia stem rot (*Sclerotinia sclerotiorum*), Alternaria blight (*Alternaria brassicae*), White rust (*Albugo candida*), Downy mildew (*Hyaloperonospora parasitica*), Powdery mildew (*Erysiphe cruciferarum*), and Blackleg (*Leptoshaeria maculans*). From discussing studies on pathogen prevalence in *B. juncea*, the review then focuses on highlighting the resistance sources and quantitative trait loci/gene identified so far from Brassicaceae and non-filial sources against these fungal pathogens. The problems in the identification of resistance sources for *B. juncea* concerning genome complexity in host subpopulation and pathotypes were addressed. Emphasis has been laid on more elaborate and coordinated research to identify and deploy R genes, robust techniques, and research materials. Examples of fully characterized genes conferring resistance have been discussed that can be transformed into *B. juncea* using advanced genomics tools. Lastly, effective strategies for *B. juncea* improvement through introgression of novel R genes, development of pre-breeding resistant lines, characterization of pathotypes, and defense-related secondary metabolites have been provided suggesting the plan for the development of resistant *B. juncea*.

## Introduction

The Brassicaceae family has about 3709 species and 338 genera, displaying enormous diversity and used as a source of oil, vegetables, mustard condiments, and fodder (Warwick et al., 2006). *Brassica juncea* (L.) Czern & Coss is a natural amphidiploid (AABB, 2n = 36) of *Brassica rapa* (AA, 2n = 20) and *Brassica nigra* (BB, 2n = 16) belonging to this family and cultivated worldwide for its edible oil. The estimated area, production, and yield of rapeseed-mustard in the world were 36.59 Mha, 72.37 Mt, and 1980 kg/ha, respectively, during 2018–2019. India contributed 19.8 and 9.8% to global acreage and production, respectively (USDA, 2020). It is presumed that this species originated approximately 0.039-0.055 million years ago (Yang et al., 2016) and the present *B. juncea* species evolved through chromosomal triplications and other re-arrangements (Lysak et al., 2005). The linkage map of *B. juncea* showed that both parental genomes were conserved and have remained unchanged since hybridization (Axelsson et al., 2000). This evolution theory is supported by the genome assemblies of *B. rapa* (Wang et al., 2011; Cai et al., 2017; Zhang et al., 2018), *B. nigra* (Yang et al., 2016; Perumal et al., 2020), *B. juncea* (Yang et al., 2016; Paritosh et al., 2020), *B. napus* (Chalhoub et al., 2014; Bayer et al., 2017; Lee et al., 2020), and the pan-genomes of *B. napus* (Song et al., 2020). The A and B genomes of *B. juncea* belong to the two different lineages of the Brassicaceae family namely Nigra and Rapa/Oleracea, thus contains the most dissimilar genomes (Kaur et al., 2014).

The genetic uniformity between all cultivated varieties of *B. juncea* makes it vulnerable to pathogen attacks. The major biotic diseases and pathogens of mustard that cause a serious threat to *B. juncea* worldwide are Alternaria blight [*Alternaria brassicae* (Berk.) Sacc.; *A. brassicicola* (Schwein.) Wiltshire; *A. raphani* Groves & Skolko], Sclerotinia stem rot [*Sclerotinia sclerotiorum* (Lib.) deBary], White rust [*Albugo candida* (Pers.) Kunze], Clubroot [*Plasmodiophora brassicae* Woronin], Powdery mildew [*Erysiphe polygoni* DC.], Blackleg [*Leptosphaeria maculans* (Desmaz.) Ces.& De Not.], and Downy mildew [*Hyaloperonospora parasitica* (Pers.: Fr.) Fr.] (Williams and Saha, 1993). These diseases cause serious damage to mustard production worldwide owing to the lack of vertical and horizontal resistance against pathogens in current cultivating varieties. Disease management through chemical fungicides is not climate-resilient and also economically not suitable. However, despite cultural adaptations very limited efficiency in disease management has been demonstrated. The host’s genetic resistance is the most effective and consistent means to control diseases (Ren et al., 2016). Fortunately, the resistant gene(s) and quantitative trait locus (QTLs) for most of the aforementioned pathogens have been identified within the Brassicaceae family, with some wild members as a treasure trove of resistant genes (Table 1).

**TABLE 1:** The resistance genes/QTLs that have been mapped in Brassicaceae family in relation of major fungal pathogens of *B. juncea* (*Sclerotinia sclerotiorum*, *Alternaria brassicae*, *Albugo candida*, *Hyaloperonospora parasitica*, *Erysiphe cruciferarum, Leptosphaeria maculans*).

Pathogen and disease	QTLs/R genes mapped
*Sclerotinia sclerotiorum*, a necrotrophic pathogen causing stem rot disease	• Three QTLs were found associated with leaf resistance at the seedling and another 3 were identified at mature plant stage in *B. napus* lines. Out of them two major QTLs (qSRM1 and qLRS1) were present on LG 15 and 17, respectively (Zhao and Meng, 2003). • A total of nine QTLs for SSR resistance were identified at chromosome A2, A3, A5, C2, C4, C6, and C9 in two segregating DH populations derived from *B. napus* (Zhao et al., 2006). • Total 21 QTLs were found associated with A3, A4, C1, C2, and C7 in DH population derived from *B. napus* DH821 (R) × DHBao604 (S) cross (Yin et al., 2010). • Out of 13 QTLs for stem and leaf resistance, one major QTL was found on C06 chromosomes associated with candidate resistant gene BnaC.IGMT5.a for SSR (Wu et al., 2013). • The biparental population derived from resistant wild *B. oleracea* (*B. incana*) with susceptible *B. oleracea* var. *alboglabra* exhibited a total of 12 and 6 QTLs for leaves and stem resistance, respectively. The candidate R genes were identified at on C09 (Mei et al., 2013). • A total of six and five QTLs were identified for SSR resistance in DH population of European winter × Chinese semi-winter rapeseed in field and controlled conditions, respectively while 17 QTLs for flowering time were associated with SSR QTLs on LG A02 and C02 (Wei et al., 2014). • A total of 35 QTLs, including 8 leaf resistances and 27 stem resistances were identified on chromosome A9 and C6 in *B. napus* (Li et al., 2015). • Chromosome C04, C06, and C08 were harbored putative QTLs for SSR resistance in *B. napus* (Wu et al., 2016). • Three QTLs of SSR resistance were identified on A08, C06, and C09 chromosomes in *B. napus* (Gyawali et al., 2016). • Introgressions in B-genome from *B. fruticulosa* to *B. juncea* identified a total of 10 marker trait associations for SSR resistance (Rana et al., 2017). • Six marker loci have been identified for SSR resistance in A and B genomes (A03, A06, and B03 chromosomes) from *B. juncea*–*Erucastrum cardaminoid* introgression lines (Rana et al., 2019). • *B. napus* chromosomes A02, A03, C02, and C06 were found to have QTLs for SSR resistance in association with QTLs of flowering time (Wu et al., 2019). • The biparental population derived from *B. napus* var. Zhongshuang 9 and *B. incana* was used to identify three resistant QTLs for SSR present on C01, C09-1, and C09-2 chromosomes (Mei et al., 2020). • A total of three major QTLs were identified through transcriptome sequencing for harboring 36 putative candidate genes for SSR resistance in *B. napus* (Qasim et al., 2020).
*Alternaria brassicae* and *A. brassicicola*, a necrotrophic fungus causing blight disease	• The transgenic *B. juncea* cv. RLM-198 for hevein (a chitin binding lectin protein) was evaluated resistant against *A. brassicae* (Kanrar et al., 2002). • The transgenic *B. juncea* for tomato glucanase conferred higher resistance against *A. brassicae* (Mondal et al., 2007). • The transgenic *B. juncea* plants with barley antifungal genes class II chitinase (AAA56786) and type I ribosome-inactivating protein (RIP; AAA32951) was reported resistant against *A. brassicae* (Chhikara et al., 2012). • PmAMP1, a cysteine-rich antimicrobial peptide from *Pinus monticola* provides resistance to *B. napus* against multiple fungal plant pathogens including *A. brassicae, L. maculans*, and *S. sclerotiorum* (Verma et al., 2012). • PR-1, PR-2, PR-3, NPR-1, and PDF1.2 were reported for resistance in the seedlings of *B. juncea* and *S. alba* (Nayanakantha et al., 2016). • A total six QTLs were identified in *A. thaliana* governing resistance against *A. brassicae*. The RtAbeCvG2-1 and RtAbeCZ5-1 QTLs consisted of 55 and 27 probable candidate genes with known functions in disease resistance pathways, respectively. The other genes present in this region were BIR1, MYC3, ERFs, BRG, RBOHD, and NPR3 including 14 defensin and defensin-like genes known for antimicrobial/antifungal activities (Rajarammohan et al., 2017).
*Albugo candida*, an obligate pathogen causing white rust in crucifers	• The *Raphanus sativus* cv. caudatus carried a dominant resistant gene for *A. candida* race 1 designated as AC-1 (Humaydan and Williams, 1976). • The white rust resistant gene (Acr) linked RFLP markers were identified in *B. juncea* (Cheung et al., 1998). • The mapping (RAC1, RAC2, and RAC3) and cloning of white rust resistant genes (RAC1) were reported from *A. thaliana* (Accession: Ksk-1 and Ksk-2) (Borhan et al., 2001). • The white rust resistant gene, WRR4 was cloned from *A. thaliana* (Accession: Col) for three races of *A. thaliana* (race 2, 4, 7, and 9) (Borhan et al., 2008). • A large number of resistant genes were identified in different Brassica species such as in *B. juncea* (Acr, Cheung et al., 1998; AC-21, Prabhu et al., 1998; AC-2, Varshney et al., 2004; ACB1-A4.1 and ACB1-a5.1, Massand et al., 2010), *B. rapa* (ACA1, Kole et al., 1996), and *B. napus* (ACA1, Ferreira et al., 1994; AC 2V1, Somers et al., 2002) conferring resistance against one or more races of *A. candida*. • The IP markers At5g41560 and At2g36360 derived from *A. thaliana* were successfully validated to have a close link with white rust resistant loci AcB1-A4.1 and AcB1-A5.1 of *B. juncea*, respectively (Singh et al., 2015). • The *A. thaliana* possesses WRR4A, WRR4B, WRR8, WRR9, and WRR12 genes for resistance (Cevik et al., 2019). • The *B. juncea*—Donskaja-IV mapped for conferring resistance locus (AcB1-A5.1) that possess R gene (BjuWRR1) (Arora et al., 2019). • The mapping population of *B. juncea* var. Tumida (resistant) × *B. juncea* var. Varuna (susceptible) was identified with a new white rust resistance-conferring locus on A6 (BjuA046215) (Bhayana et al., 2020).
*Hyaloperonospora parasitica*, an obligate pathogen causing downy mildew disease	• A single dominant gene conferred resistance was identified in *B. napus* (Lucas et al., 1988). • A single dominant gene against race-2 was identified in broccoli (*B. oleracea*) (Dickson and Petzoldt, 1993). • Partially dominant gene was identified in resistant accessions RES-02 and RES-26 of *B. napus* (Nashaat et al., 1997). • A dominant and single R gene (Pp523) was identified in broccoli expressed at the adult plant stage (Coelho et al., 1998). • The DH population of broccoli (*B. oleracea*) was identified for partial resistance at cotyledon stage (Jensen et al., 1999). • *B. oleracea* (DH broccoli) was identified for two unlinked dominant genes at seedling stage (Wang et al., 2001). • *A. thaliana* was identified with downy mildew resistant genes RPP9 and RPP8 (Borhan et al., 2001). • A recessive resistant gene was identified in *B. oleracea* at cotyledon stage (Carlsson et al., 2004). • *A. thaliana* was encodes for TIR- and CC-NBS-LRR categories R genes such as RPP5 (Parker et al., 1997), RPP8 (McDowell et al., 1998), RPP1 (Botella et al., 1998), RPP13 (Bittner-Eddy et al., 2000), RPP4 (Van Der Biezen et al., 2002), and RPP2A/RPP2B (Sinapidou et al., 2004). • A major gene (RPP31) for adult stage resistance was genetically mapped *in A. thaliana* on chromosome 5 (McDowell et al., 2005).
	• Two dominant genes at cotyledonary and one at adult plant stage were identified in *B. oleracea* mapping population (Monteiro et al., 2005). • *B. oleracea* possessed R gene (Pp523) on chromosome C8 (Carlier et al., 2011) with the syntenic region at the top arm of *A. thaliana* chromosome 1 (Farinhó et al., 2007). • A single dominant gene BrRHP1 on chromosome 1 of *B. rapa* was identified (Kim et al., 2011). • A total of two SSRs (kbrb006c05-2 and kbrb058m10-1) and one SCAR (SCK14-825) markers were found closely linked to downy mildew resistant QTL BrDW on chromosome A8 in *Brassica rapa* ssp. *pekinensis* (Yu et al., 2011). • A single dominant R gene, Ppa3 was identified in *B. oleracea* (Singh et al., 2012). • A total of four major QTLs (sBrDM8, yBrDM8, rBrDM8, and hBrDM8) were mapped to A08 through three SNP markers (A08-709, A08-028, and A08-018) for resistance at the seedling, young plant, rosette and heading stages in *B. rapa* (Yu et al., 2016).
*Erysiphe cruciferarum*, an obligate pathogen causing powdery mildew disease	• A total of six *A. thaliana* accessions were identified to confer resistance through single/double locus (RPW1, RPW2, RPW3, RPW4, RPW5) functioning independently (Adam and Somerville, 1996). • Two independent segregating dominant loci (RPW6 and RPW7) on chromosome 5 and 3 in *A. thaliana* were identified (Xiao et al., 1997). • The transgenic of *N. tabacum* developed with RPW8.1 and RPW8.2 genes confers higher resistance (Xiao et al., 2003). • The R genes characterized in *A. thaliana* were NDR1, EDS1, PAD4, NPR1, EDS5, RAR1, SGT1b, RPW8, PBS3, COI1, EIN2, and EDR1 (Xiao et al., 2005). • A total of one and three genes homologous to R gene (RPW8) were present in *B. rapa* and *B. oleracea*, respectively (Li et al., 2016).
*Leptosphaeria maculans*, a hemibiotroph pathogen causing blackleg	• A total 17 QTLs on 13 linkage groups were identified in mapping population of *B. napus* DH lines derived from Darmor-bzh(R) × Yudal(S) (Huang et al., 2016). • *B. napus* DH lines from cultivar “Cresar”(R) × “Wester”(S) has LmR1 resistant gene and LepR4 recessive gene on A- genome (Yu et al., 2013). • Resistant gene Rlm1 was mapped on chromosome A7 in B. napus DH lines from “Maxol” × ‘’Columbus” (Raman et al., 2012a). • A major QTL, Rlm4 was mapped on chromosome A7 in B. napus population derived from “Skiptone” × “Ag-spectrum” (Raman et al., 2012b). • *B. napus* cv. Surpass400 was mapped for a single dominant allele LepR3 on same linkage group and below LepR2 of A- genome (Yu et al., 2008). • The resistant genes LepR1 and LepR2 were introgressed from *B. rapa* subsp. *sylvestris* to DH population of *B. napus* (DHP95 and DHP96) (Yu et al., 2005). • *B. juncea* (Bj168) was used to introgress Rlm6 resistant gene in *B. napus* lines (Chevre et al., 1997). • Two resistant genes (LMJR1-Resistant and LMJR2-Recessive) for blackleg was mapped in B. juncea cv. AC Vulcan (Christianson et al., 2006) • A recessive resistant gene rjlm2 was introgressed from *B. juncea* cv. Stoke to *B. napus* (Saal and Struss, 2005). • A dominant resistant gene PhR2 was transferred from *B. juncea* cv. Stoke to *B. napus* (Plieske and Struss, 2001). • LmBR1, a dominant resistant gene was introgressed in *B. napus* from *B. juncea* cv. GrGC No.4 (Dixelius, 1999).

The plants respond to pathogens either through constitutive or inducible defense mechanisms to protect themselves from damage. Constitutive defense includes preformed physical barriers such as cell walls, cuticle, wax on the outer surface, bark, and cork layers. Alternately, plants produce toxic chemicals, cell lytic enzymes, and deliberate cell suicide when attacked by pathogens, which form the inducible defense systems. The inducible defense system of the plants activated only after a pathogen is recognized by his immune responsive system because of the production of chemicals or proteins. This leads to high energy costs and nutrient requirements in association with their production and management. A lot of plant pathogens, known as biotrophs, make a close connection with their host plant suppressing its defense system but keeping their host alive, use the plant’s nutrients for their growth and reproduction. However, some pathogens produced toxins, cell degrading enzymes, or proteins to overcome plant defense systems promoting the rapid release of nutrients called necrotrophs. The pathogens can infect more than one host plant called the host range. Similarly, plants can defend themselves against a particular, or wide range of pathogens, or races of the pathogen. This type of plant defense is called vertical and horizontal resistance, respectively. The vertical resistance of the host plant can protect from a particular type of pathogen or race with high intensity but does not last long against the pathogen. The vertical resistance of the host plants is due to one or two major genes and is highly specific. However, the horizontal resistance is durable and it can protect host plants against a wide range of pathogens and races. In this, a group of genes simultaneously take part in host defense and present within the QTL. The genes that take part in the host defense systems are called R genes. The plant pathogenic fungi and bacteria have Avr (avirulence) genes to identify their host plants and infect them. The Avr genes produce small secreted protein (SSP) effectors and secondary metabolites (SM), the key elements of their pathogenesis that regularize innate immunity of the host plant and facilitate infection. However, the host resistance can be established only when the R gene of the host recognizes the effectors or secondary metabolites. This recognition usually triggers its defense responses including the hypersensitive response (HR) and results in resistance from the plant to the pathogen (Freeman and Beattie, 2008).

Apart from resistance genes, plants also resist pathogenic attacks via hormone signaling pathways such as Jasmonates (JAs), Salicylates (SAs), Brassinosteroids (BR), Ethylene (ET), Abscisic acid (ABA), and Phosphoglycerolipids (PGL). Out of all the above signaling pathways, JAs and SAs are reported most effective against pathogenic diseases. The polyunsaturated fatty acids (PUFA), a group of plant oxylipins produced Jasmonates (JAs) through oxidation by using one of the seven different branches of the lipoxygenase (LOX) pathway. However, the key component of JA biosynthesis is allene oxide synthase (AOS) that utilizes 13-hydroperoxide from α-linolenic acid (18:3, α-LeA). PUFA is released from the chloroplast membrane and converted into 9 or 13-hydroperoxides through the LOX pathway, which further enters into the AOS pathway to produce free JA. There are a lot of JA derivatives present in the host plants such as free JA, methyl-jasmonate (Me-JA), *cis*-jasmone, Jasmonyl isoleucine (JA-Ile), and Jasmonoyl ACC (JA-ACC). The role of free JA and Me-JA is well established under various biotic and abiotic stress conditions (Sirhindi et al., 2017). Salicylic acid (SA) is a phenolic plant hormone that actively participates in defense mechanisms under biotic and abiotic stresses to protect the host plant. For instance, the basal resistance for the fungal pathogen *B. cinerea* is regulated by SA in tomato but by jasmonic acid and ethylene in tobacco (Loon et al., 2006). The key enzymes of the phosphoglycerolipid (PGL) pathways are phospholipase C (PLC) and phospholipase D (PLD) capable of producing phosphatidic acid (PA). Phosphatidic acid acts as a signaling molecule by binding to target proteins altering their subcellular localization and enzymatic activity. This signaling pathway is activated during plant exposure to both biotic and abiotic stresses (Pokotylo et al., 2017). The hormone signaling pathways functions systemically in host plants, thus labeled as the systemic acquired resistance (SAR) effective against a wide range of pathogens. These plant defense mechanisms regulated through resistance genes and hormone signaling pathways under strict control (Larkan et al., 2013; Ma and Borhan, 2015) exhibited highly complex and specific immune responses (Stotz et al., 2014). Very few R genes have been characterized and cloned as yet, thus resistance breeding programs are slow as they depend on chemical management. However, the next-generation sequencing projects have developed highly specific thorough genomic data of diploid and polyploidy plants, providing an opportunity to identify resistance genes.

**FIGURE 1:**
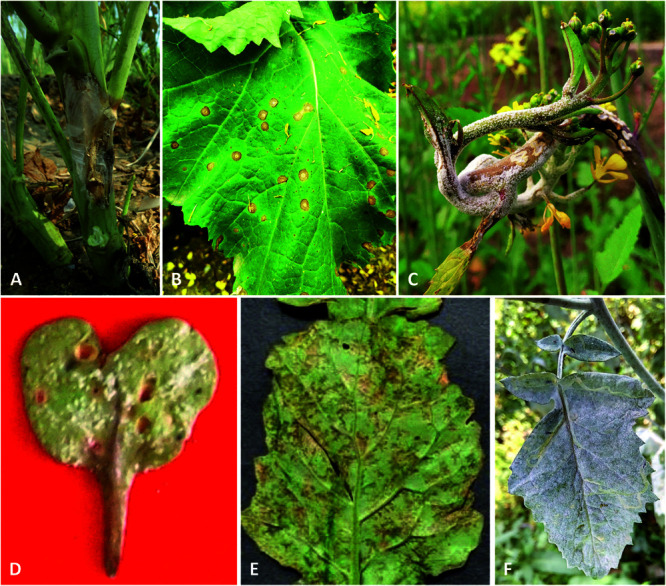
Major fungal diseases of *B. juncea*. **(A)** Sclerotinia stem rot; **(B)** Alternaria leaf blight; **(C)** Staghead formation in flower buds of *B. juncea* by *A. candida*; **(D,E)** Downy mildew symptoms at lower surface of cotyledon and mature leaf, respectively (Photo courtesy: A. K. Tewari); **(F)** Powdery mildew of *B. juncea*.

The present study aims to review the work done on identifying the sources of these resistance genes/QTLs across the Brassicaceae family. This knowledge may help improve the resistance of *B. juncea* by focusing on major pathogens and corresponding diseases such as Sclerotinia stem rot, Alternaria blight, White rust, Downy mildew, and Powdery mildew (Figure 1). We shall confirm the sources of resistance genes for these pathogens in Brassicaceae to support their potential use in resistance breeding of *B. juncea*. Also, the challenges in identification and the use of resistance genes, and effective strategies to use resistance genes in the development of resistant *B. juncea* lines for such major pathogens will be discussed.

## Resistance Sources for Major Pathogens

### Sclerotinia Stem Rot

The *Sclerotinia sclerotiorum* (Lib.) deBary is a necrotrophic fungal pathogen causing stem rot disease in *B. juncea* worldwide (Ghasolia et al., 2004; Singh et al., 2008). Severe outbreaks of stem rot disease under cool and moist weather conditions (Purdy, 1979; Saharan and Mehta, 2008) cause heavy yield losses ranging between 5 to 100% (Ghasolia et al., 2004; McDonald and Boland, 2004; Shukla, 2005). Sclerotinia stem rot (SSR) is also now recognized as a major yield-limiting disease of *B. napus* in Australia, Canada, China, Europe, and India (Rana et al., 2017). However, the management of stem rot by traditional and/or biochemical practices has been a cumbersome process, owing to its wide host range (about 408 plant species of 75 families) and the potential to survive in harsh environmental conditions by forming sclerotia (Boland and Hall, 1994; Peltier et al., 2012). Genetic diversity studies of *S. sclerotiorum* have been conducted since last three decades in different parts of the world including Australia, Brazil, Canada, India, Iran, United Kingdom, and the United States (Kohn et al., 1991; Sexton et al., 2006; Hemmati et al., 2009; Litholdo et al., 2011; Barari et al., 2012; Clarkson et al., 2012; Mandal and Dubey, 2012; Attanayake et al., 2014). Therefore, instead of using traditional practices and chemicals, the cultivation of resistant varieties is the most economical and climate-resilient approach to manage SSR in the mustard crop. Resistant cultivars can efficiently minimize yield losses due to the disease (Westman et al., 1999; Taylor et al., 2002). Thus, the resistance sources were investigated within the Brassicaceae and out of the family to identify potential R genes for *B. juncea* resistance breedings and introgressions. The resistance screening experiments were mainly focused on leaf and stem at cotyledonary and adult plant stages under *in vitro* and/or field conditions (Garg et al., 2008; Li et al., 2008; Barbetti et al., 2014; Uloth et al., 2014; Ge et al., 2015; Naher et al., 2018; Kumari and Singh, 2019).

The species displaying a promising resistance against the SSR disease are *Brassica fruticulosa*, wild *Brassica oleracea, Brassica napus, Camelina sativa, Diplotaxis tenuisiliqua, Erucastrum abyssinicum*, *E. cardaminoides*, and *Sinapis alba* (Morrall and Dueck, 1982; Kolte, 1985; Garg et al., 2010; Mei et al., 2013; Wu et al., 2013; Rana et al., 2017, 2019; Purnamasari et al., 2019). These species were used to introgress resistance in the cultivated oilseed Brassicas. The introgression lines were developed through somatic hybridization or embryo rescue, followed by subsequent backcrossing and/or selfing. The wild crucifers viz. *Erucastrum cardaminoides*, *Diplotaxis tenuisiliqua*, and *E. abyssinicum* were used for resistance introgressions in *B. juncea* and *B. napus* and then evaluated for a higher level of resistance as compared to 54 Brassica germplasms of Australia, China, and India (Garg et al., 2010). These introgression lines were then used to locate the candidate gene or QTL for *S. sclerotiorum* resistance and physically mapped through advanced molecular tools. Rana et al. (2017) produced fertile *B. juncea*–*B. fruticulosa* introgression lines with segmental introgressions in B-genome chromosomes at terminal positions. All these lines were resistant and genotyped with 262 microsatellite markers. They have identified a total of ten significant marker-trait associations through association mapping. In another study, Rana et al. (2019) developed a set of 96 *B. juncea*–*Erucastrum cardaminoid* introgression lines for SSR resistance. All these introgression lines were genotyped by transferable microsatellite markers, followed by genotyping by sequencing to establish marker-trait associations. They have detected a total of six marker loci in both A and B genomes associated with resistance through SSR markers. However, the genome-wide association analysis (GWAS) identified a large number of single nucleotide polymorphisms (SNPs) linked to resistance in A03, A06, and B03 chromosomes. The annotation studies identified an array of resistance mechanisms, i.e., signal transduction pathways, hypersensitive responses, and production of anti-fungal proteins and metabolites. A total of five SNPs on the A03 chromosome were found to be associated with LRR-RLK genes that encoded LRR-protein kinase family proteins. They also predicted genetic factors associated with pathogen-associated molecular patterns (PAMPs) and effector-triggered immunity (ETI) on the A03 chromosome. These belonged to three R-Genes encoding TIR-NBS-LRR proteins. Hitherto, no resistant gene has been cloned for this disease (Wu et al., 2016, 2019).

The population derived from the resistant wild *B. oleracea* (*B. incana*) with the susceptible *B. oleracea* var. *alboglabra* was used to identify QTL for SSR. The F_2_ population exhibited a total of 12 and 6 QTLs for leaves and stem resistance, respectively. The candidate R genes were identified for Sclerotinia resistance on C09 of *B. oleracea* by blasting the sequence of adjacent markers to the *B. rapa* reference genome. The 12.8-cM genetic region on C09 contains two major QTLs for both leaf and stem resistance, while the corresponding 2.7-Mb genomic region on chromosome A09 of *B. rapa* harbors about 30 genes putatively encoding resistance-related and defense associated proteins. The putative genes present on the A09 chromosome belonged to the CC-NBS-LRR class (Mei et al., 2013).

Furthermore, the *B. napus* has been evaluated by many to identify putative QTLs for SSR resistance. The GWAS for SSR resistance in *B. napus* had identified three putative QTLs on C04, C06, and C08 (Wu et al., 2016), A08, C06, and C09 chromosomes (Gyawali et al., 2016). The chromosome A02, A03, C02, and C06 of *B. napus* were identified to have QTLs for SSR resistance through SNP-array genotyping. However, it was recorded that these genomic regions also harbored QTLs for flowering time (Wu et al., 2019). Mei et al. (2020) developed a BC_1_F_8_ population of *B. napus* var. Zhongshuang 9 and *B. incana* for pyramiding of three resistant QTLs for SSR present on C01, C09-1, and C09-2 chromosomes. They concluded that all QTLs worked cumulatively for disease and the lines having all three QTLs showed the highest resistant level. Qasim et al. (2020) identified three major QTLs through comparative transcriptomic studies harboring 36 putative candidate genes from resistant *B. napus* lines that might actively involved in SSR resistance. Wu et al. (2013) identified 10 and 3 QTLs for stem and leaf resistance in *B. napus*, respectively. Out of these, a major QTL present on the C06 chromosome was associated with the BnaC.IGMT5, a candidate resistant-gene for SSR. Theoretically, there could be a strong correlation between leaf and stem resistance as noticed in various studies of Brassica species (Mei et al., 2012; Wu et al., 2013; You et al., 2016). Yet, this observation could not be correlated practically when some genotypes of Brassica were evaluated for SSR resistance (Uloth et al., 2013; You et al., 2016). Nevertheless, some other genotypes share common genomic regions for leaf and stem resistance (Li et al., 2015). Such contradictory findings indicate that the resistance against SSR in leaves and stem is specific at subpopulation levels. The inheritance of common resistance genes on both genomes (A and C) indicated a common ancestry of chromosomes (Wei et al., 2014).

### Alternaria Blight

Alternaria is a key pathogenic genera of the family Brassicaceae, causing severe damage to host plants and one of the major yield-limiting factors. The *Alternaria* genus was first identified by Nees Von Esenbeck (1817) and Berkeley (1836) as a causal pathogen of leaf blight on Brassicaceae plants as *Macrosporium brassicae* Berk., which was later renamed as *Alternaria brassicae* (Berk.) (Saccardo, 1886). The blight disease of *B. juncea* is responsible for heavy yield losses ranging upto 47% in India (Kolte, 1985) and 32–57% in Nepal (Shrestha et al., 2005). The pathogen has a wide host range in cruciferous crops and is reported from all parts of the world including Canada (Berkenkamp and Kirkham, 1989; Conn and Tewari, 1990), India (Kadian and Saharan, 1983), Iran (Nourani et al., 2008), Italy (Tosi and Zezzerini, 1985), Poland (Nowicki et al., 2012), and United States, United Kingdom, Europe (Gladders, 1987). This pathogen attacks leaves, stem, and siliquae at all growth stages producing characteristic disease symptoms. However, the blighted spots produced by *A. brassicae* are gray as compared to the black sooty velvety spots of *A. brassicicola*, another leaf blight pathogen of mustard (Meena et al., 2010; Kumari and Singh, 2019). The infection spot leads to the formation of a necrotic zone which looks like a target board due to the interrupted growth of the pathogen. The pathogen resides at the center of the lesion surrounded by an uninvaded chlorotic halo created by the diffusion of pathogen toxins (Tewari, 1983; Agarwal et al., 1997). The Indian isolates of *A. brassicae* were grouped under three categories according to their virulence level as A (highly virulent), C (moderately virulent), and D (avirulent) collected from all over India (Vishwanath and Kolte, 1997). A total of 32 isolates of *A. brassicae* were examined by Random Amplified Polymorphic DNA (RAPD) and Inter Simple Sequence Repeat (ISSR) markers for genetic variability. The results of the study revealed that there was no clear grouping of isolates but little variations were recorded which may be due to ecological or survival-based adaptations (Sharma et al., 2013).

The pathogen has a very high survival rate on Brassica host debris and other alternative hosts (*Anagallis arvensis, Convolvulus arvensis*) carry the inoculum from one crop season to another (Tripathi and Kaushik, 1984; Verma and Saharan, 1994; Mehta et al., 2002). Thus, the air-borne spores of *A. brassicae* are the prime source of the inoculum of this polycyclic disease (Kolte, 1985). The pathogen can infect host plants through epidermis penetration, stomata, insects- or wounds. However, *A. brassicae* preferred stomatal penetration while *A*. *brassicicola* penetrated directly through the epidermis (Nowicki et al., 2012). The *A. brassicae* produced host-specific toxins (HST) during infection characterized as ABR toxin protein (Parada et al., 2008; Wight et al., 2009). This compound plays a vital role in the pathogenesis, also determining the virulence and pathogenicity level of the pathogen (Nishimura and Kohmoto, 1983). However, some additional toxins like destruxin B (Bains and Tewari, 1987) and it’s derivatives (homodestruxin B, desmethyldestruxin B, and destruxin B2) are also produced. All these toxins are responsible for the typical tissue necrosis and chlorosis. These toxins stimulate the phytoalexins, brassilexin, and sinalbin A (Sodelade et al., 2012). The *A. brassicae* produces abscisic acid causing premature leaf aging and defoliation, dropping of flowers, and premature siliquae breaking (Tewari, 1991b), while cytokines are responsible for green discolorations within the blighted spots (Tylkowska et al., 2004).

It was recorded that when *Brassica rapa* seedlings were inoculated with the fungal pathogen *A*. *brassicae*, 3-butenyl, and 4-pentenyl isothiocyanates were released together with dimethyl disulfide, dimethyl trisulphide, 4-oxoisophorone, and several sesquiterpenes (Kevin et al., 1996). The release of isothiocyanates is evidence for the catabolism of glucosinolates during infection, which is a prerequisite for their involvement in resistance (Doughty et al., 1996). The host resistance is the most economical and feasible mode to control yield losses. This approach allows us to decrease pesticide usage and also lowers the chemical pollution. The resistance breeding programs for Brassica improvement are hindered due to bottlenecks in the resistance introgression from the wild species into the commercial crops. The cultivated vegetable and oilseed Brassicas are devoid of genetic resistance against Alternaria blight pathogens. However, among the Brassica crops, the Ethiopian mustard has displayed the highest level of resistance against *A. brassicae*. The wild members of the Brassicaceae family such as *S*. *alba* (Kolte, 1985; Brun et al., 1987; Ripley et al., 1992; Sharma and Singh, 1992; Hansen and Earle, 1995, 1997; Kumari et al., 2018, 2020b), *Camelina sativa*, *Capsella bursa-pastoris*, *Eruca sativa, Neslia paniculata*, *Alliaria petiolata, Barbarea vulgaris, Brassica elongate, B. desnottessi, B. fruticulosa*, *B. maurorum*, *B. nigra*, *B. souliei*, *B. spinescens*, *Camelina sativa*, *Capsella bursa-pastoris*, *Coincya* spp., *Diplotaxis catholica*, *D. berthautii*, *D. creacea*, *D. erucoides*, *D. tenuifolia*, *Erucastrum gallicum, Eruca vesicaria* subsp. *sativa*, *Hemicrambe fruticulosa*, *H. matronalis*, *Neslia paniculata, Raphanus sativus*, and *S. arvensis* (Conn and Tewari, 1986; Brun et al., 1987; Conn et al., 1988; Tewari, 1991a; Zhu and Spanier, 1991; Tewari and Conn, 1993; Sharma et al., 2002; Warwick, 2011) reportedly possess the highest level of resistance against *A. brassicae*.

*Camelina sativa* produces phytoalexin on the landing of conidia on leaves and accumulates near and under conidial drops. This phytoalexin accumulation is a rapid response initiated just after conidial inoculation solely responsible for the inhibition of pathogen growth on the leaf surface (Jejelowo et al., 1991). The *C. sativa* produces two types of phytoalexins in leaves after elicitation by the pathogen, namely camalexin (C11N8N2S) and 6-methoxycamalexin (C H N SO) (Browne et al., 1991). The pathogenic attack elicits the production of camalexin, 6-methyloxycamalexin, and *N*-methylcamalexin (C H N S) in *Capsella bursa-pastoris* (Jimenez et al., 1997). The mutant Arabidopsis (phytoalexin deficient) is devoid of camalexin biosynthesis displaying higher susceptibility to *A. brassicicola*, establishing the contribution of camalexin in host resistance (Thomma et al., 1999b). However, it appears that the defense against *A. brassicicola* in Arabidopsis depends on multiple components, as Jasmonate insensitive Arabidopsis mutant coi1 was found vulnerable to this pathogen. This indicates that both components contribute to host resistance against *A. brassicicola* pathogen separately but in parallel pathways (Thomma et al., 1998, 1999a, 2000, 2001). The pathogen initiates signaling cascade through MAPK3 inducing the expression of LOX which further regulates the expression of MAPK3. Interaction of both the genes initiates the Jasmonate acid biosynthesis which induces the defense mechanism in *B. juncea* (Taj et al., 2011). The *S. alba* produces two types of phytoalexins (sinalexin) sinalbins A and B in the leaf and stem tissues for resistance against the *A. brassicae* (Pedras and Smith, 1997; Pedras and Zaharia, 2000). The variation in the elicitation level of phytoalexin is believed to be responsible for resistance status in plants (Conn et al., 1988).

Nayanakantha et al. (2016) examined five defense-related genes viz., PR-1, PR-2, PR-3, non-expresser of PR-1 (NPR-1), and plant defensin (PDF1.2) after inoculation of the seedlings of *B. juncea* and *S. alba*. The transcripts of all five defense-related genes accumulated locally as well as systemically at a greater level and earlier in *S. alba* than in *B. juncea* upon challenge inoculation with *A. brassicae*. The PDF1.2 was induced by SA and PR-1 by JA in both *B. juncea* and *S. alba* resulting in both JA and SA responsive genes conferring resistance against *A. brassicae* in *S. alba*. Similarly, upon challenging with *A. brassicicola*, the *S. alba* plants showed higher accumulation of abscisic acid and JA than *B. juncea* (Mazumder et al., 2013). The molecular basis of resistance could not be uncovered in *S. alba* due to the lack of molecular markers. However, the recently developed *de novo* genome assembly and a large number of microsatellites may assist to identify the potential genomic regions of resistance (Kumari et al., 2020a). The transgenic *B. juncea* cv. RLM-198 for hevein (a chitin binding lectin protein) was developed and evaluated against *A. brassicae* infection under glasshouse conditions. It was found that transgenics showed higher resistance levels for diseases such as longer incubation and latent period, smaller necrotic lesion size, lower disease intensity, and delayed senescence (Kanrar et al., 2002). Chhikara et al. (2012) developed transgenic *B. juncea* plants with barley antifungal genes class II chitinase (AAA56786) and type I ribosome-inactivating protein (RIP; AAA32951) to coexpress. These transgenics were evaluated *in vitro* and greenhouse conditions for resistance against *A. brassicae*. The study revealed a 44% reduction in hyphal growth, delayed onset of the disease, and a limited number of lesions as compared to wild resistant plants (Mondal et al., 2003). The transgenic *B. juncea* for tomato glucanase conferred higher resistance against *A. brassicae* under *in vitro* and polyhouse conditions (Mondal et al., 2007). Rajarammohan et al. (2017) developed three bi-parental mapping populations from three resistant (CIBC-5, Ei-2, and Cvi-0) and two susceptible (Gre-0 and Zdr-1) accessions. A total of five population-specific and one non-specific QTLs were identified governing resistance against *A. brassicae*. The presence of population-specific and non-specific QTLs indicated the quantitative nature of resistance to *A. brassicae*. The RtAbeCvG2-1 QTL consisted of 55 probable candidate genes with known functions in disease resistance pathways. A total of 27 candidate genes were identified at RtAbeCZ5-1 QTL on chromosome 5, out of which 24 belong to TIR-NBS-LRR and three genes to the CC-NBS-LRR class. The other genes present in this region involved in defense signaling pathways include BIR1 (BAK1-interacting receptor-like kinase), MYC3 (JAZ-interacting transcription factor), ERFs (Ethylene response factors), BRG (Botrytis-induced Related Gene), RBOHD (Respiratory Burst Oxidative Homolog D), NPR3 (Non-expressor of PR3) including 14 defensin and defensin-like genes known for antimicrobial/antifungal activities. The exogenous application of β-Aminobutyric Acid to *B. juncea* prevented plants from *A. brassicae* infection independent of SA and JA signaling pathways (Kamble and Bhargava, 2007). The cysteine-rich antimicrobial peptide (PmAMP1) from *Pinus monticola* provides resistance to *B. napus* against multiple fungal plant pathogens including *A. brassicae, Leptosphaeria maculans*, and *S. sclerotiorum* (Verma et al., 2012). Vishwanath et al. (1999) induced resistance in susceptible *B. juncea* cv. PR-15 against highly virulent isolate-A of *A. brassicae* by using avirulent isolate-D.

### White Rust

*Albugo candida* (Pers. Ex. Lev.) Kuntze. is an obligate parasite of the oilseed Brassicas causing white rust and staghead disease worldwide including Brazil (Viegas and Teixeira, 1943), Canada (Petrie, 1973), China (Zhang et al., 1984), Germany (Klemm, 1938), India (Chowdhary, 1944), Japan (Hirata, 1954), Korea (Choi et al., 2011), New Zealand (Hammett, 1969), Pakistan (Perwaiz et al., 1969), Palestine (Rayss, 1938), Romania (Savulescu, 1946), Turkey (Bremer et al., 1947), United Kingdom (Berkeley, 1848), and United States (Walker, 1957). The disease can be identified as localized white to pale cream-colored spots on leaves and inflorescence (hypertrophied flowers). The formation of staghead is accounted for complete loss of seed formation which causes upto 90% yield loss. The yield loss is dependent on the disease severity and has been reported upto 60% in *Brassica rapa* L. in Canada (Petrie, 1973; Petrie and Vanterpool, 1974; Harper and Pittman, 1974), 23–89.8% of *B. juncea* in India (Bains and Jhooty, 1979; Lakra and Saharan, 1989), and about 5–10% in Australia (Barbetti, 1981; Barbetti and Carter, 1986). The *A. candida* has reportedly infected more than 63 genera and 241 species of the Brassicaceae family from all over the world (Farr et al., 1989; Saharan and Verma, 1992; Choi et al., 2007). The fungal pathogen can also infect plant species outside of the Brassicaceae family (Choi et al., 2006, 2007, 2008, 2009, 2011). The infection of pathogen leads to many biochemical changes in the host for a successful establishment of the pathogen and causes disease. The chlorophyll, sugars, and total phenol content was found in higher concentrations in resistant cultivars than the susceptible ones during all growth stages. However, the number of total proteins and free amino acids were increased in the susceptible cultivars in their life cycle (Singh, 2000). Gupta et al. (1997) identified a positive role of chlorophyll in *A. candida* resistance in *B. juncea*. A higher concentration of auxins (indole-3-acetonitrile [IAN], indole-3-acetic acid [IAA]) is produced in *A. candida* infected plants which lead to hyperplasia and hypertrophy of leaf, stem, and floral parts (Kiermayer, 1958). The abundance of nineteen proteins was found variable between the susceptible and resistant varieties of *B. juncea* after the pathogen invasion and out of these nineteen proteins, five were present in the resistant variety (Kaur et al., 2011a).

Cheung et al. (1998) identified Restriction Fragment Length Polymorphism (RFLP) markers linked to the white rust resistant gene (Acr) in *B. juncea*. The genotype non-specific intron polymorphic (IP) markers At5g41560 and At2g36360 derived from *A. thaliana* were successfully validated to have a close link with white rust resistant loci AcB1-A4.1 and AcB1-A5.1 of *B. juncea*, respectively (Singh et al., 2015). The mapping (RAC1, RAC2, and RAC3) and cloning of white rust-resistant genes (RAC1) were reported from *A. thaliana* (Accession: Ksk-1 and Ksk-2) for resistance against *A. candida*. The RAC genes belong to the Drosophila toll/interleukin-1 receptor (TIR) nucleotide-binding site leucine-rich repeat (NB-LRR) class of plant resistant gene (Borhan et al., 2001). Another white rust resistant gene, WRR4, was cloned from *A. thaliana* (Accession: Col) (Borhan et al., 2008) which encodes for the same TIR-NB-LRR protein and was found to be resistant against three races of *A. thaliana* (race 2,4,7, and 9). The expression of WRR4 in susceptible lines of *B. juncea* and *B. napus* provided complete resistance against white rust pathogen belonging to the races 2 and 7, respectively (Borhan et al., 2010).

A large number of resistant genes were identified in different Brassica species such as in *B. juncea* (Acr – Cheung et al., 1998; AC-21 – Prabhu et al., 1998; AC-2 – Varshney et al., 2004; ACB1-A4.1 and ACB1-a5.1 – Massand et al., 2010), *B. rapa* (ACA1 - Kole et al., 1996), *B. napus* (ACA1 – Ferreira et al., 1994; AC 2V1 – Somers et al., 2002), and *A. thaliana* (RAC-1, RAC-2, RAC-3, and RAC-4 – Borhan et al., 2001, 2008) conferring resistance against one or more races of *A. candida*. The *Raphanus sativus* cv. caudatus possess a dominant resistant gene for *A. candida* race 1 designated as AC-1 (Humaydan and Williams, 1976). However, some other Brassica species demonstrated monogenic control of the white rust pathogen race 2 such as *B. nigra*, *B. rapa*, *B. carinata*, and *B. juncea* (Delwiche and Williams, 1974; Ebrahimi et al., 1976; Thukral and Singh, 1986; Tiwari et al., 1988). The resistance to *A. candida* race 2 (Ac2V) can be explained in *A. thaliana* accessions by at least one of four genes (WRR4A, WRR4B, WRR8, and WRR9) encoding nucleotide-binding, leucine-rich repeat (NLR) immune receptors. However, the WRR12 gene identified in *A. thaliana* confers resistance to *A. candida* race 9 that infects *B. oleracea*. Thus, the effector-triggered immunity conferred by the distinct NLR-encoding genes in multiple *A. thaliana* accessions provides species-wide resistance to different races of *A. candida* (Volkan et al., 2019). The *B. juncea*—Donskaja-IV from the east European gene pool line was mapped for conferring resistance locus (AcB1-A5.1) against white rust and was found to be completely resistant to six different Indian isolates. The locus was identified to possess a single CC-NB-LRR protein-coding R gene (BjuWRR1) which provided resistance to genetically transformed susceptible Indian mustard variety Varuna from all the isolates (Arora et al., 2019). The F1DH mapping population of *B. juncea* var. Tumida (resistant) × *B. juncea* var. Varuna (susceptible) was identified with a new white rust resistance-conferring locus on LG A6 which was most likely to BjuA046215 candidate gene, a CC-NBS-LRR type R gene, and closely related to BjuWRR1 (Bhayana et al., 2020).

### Downy Mildew

The disease is incited by a biotrophic fungal pathogen *Hyaloperonospora parasitica* (Pers.) Constant. Syn. *H. brassicae* which causes heavy yield loss to cruciferous crops and is distributed worldwide. In India, the disease is responsible for upto 66% yield loss in *B. juncea* making up a yearly loss of about 683.1 million INR, depending on the disease severity (Meena et al., 2014). The pathogen can infect all aerial parts (leaf, stem, and flowers) of the plant. However, plant resistance depends on the plant age and environmental variations (Coelho et al., 2009). Mohammed et al. (2018) screened about 154 members of the Brassicaceae family comprising *Brassica napus*, *B. carinata*, *B. juncea*, *R. sativus*, *Rapistrum rugosum*, *B. incana*, *Crambe abyssinica*, *B. fruticulosa*, *Hirschfeldia incana*, *B. insularis*, *B. oleracea*, and *Sinapis arvensis* for resistance against seven isolates of *H. parasitica*. They found most of the resistant lines from *R. sativus*, *B. carinata*, and *B. juncea* genotypes. However, they did not conduct any molecular study to identify a downy mildew resistant gene. The downy mildew resistant and white rust susceptible *B. juncea* genotype were inoculated with *A. candida* followed by *H. parasitica* that lead to asymptomatic systemic colonization of downy mildew pathogen and a more severe infection of white rust about four days earlier (Kaur et al., 2011b). The field resistance of *B. oleracea* var. *tronchuda* can not be predicted through cotyledon resistance as both are governed by different genetic systems (Monteiro et al., 2005). Nashaat and Awasthi (1995) screened 31 *B. juncea* accessions against four isolates of *H. parasitica* at the cotyledon stage and almost all expressed high-level resistance against the pathogen. These accessions were grouped into five categories showing differential disease responses.

The homozygous resistant and susceptible varieties (USVL012 and USVL047, respectively) of *B. oleracea* were used for the development of the self and backcross population. The F_2_ population showed segregation for resistant character and backcross population developed with resistant plant remained resistant and with susceptible plant remained susceptible. Thus, they concluded that the resistant character must be governed by two complementary dominant genes (Wang et al., 2001). In similar studies on 200 *Raphanus sativus* accessions, it was confirmed that the resistance was governed by a single dominant gene (Coelho and Monteiro, 2018). Dang et al. (2000) used 36 genotypes of different varieties of Brassicaceae for resistance screening against leaf pathogens during three consecutive years and found *Brassica alba* (*S. alba*) highly resistant to downy mildew disease in all 3 years. The F_2_ population developed from the resistant and susceptible genotypes of broccoli (*B. oleracea* L. Italica Group) and cauliflower (*B. oleracea* L. var. *botrytis*) showed segregation in a 3:1 (resistant:susceptible) ratio which indicated that the resistance is governed by a single dominant gene and also confirmed by test-cross (Farnham et al., 2002; Vicente et al., 2012; Verma and Singh, 2018). It was also evident from different studies that the resistance against downy mildew pathogen in *B. napus* and *B. juncea* was also governed by a single dominant gene (Nashaat et al., 1997, 2004).

Coelho et al. (1998) identified the dominant and monogenic (Pp523) inheritance of plant resistance expressed at the adult stage in broccoli located on another genetic map of RAPD and AFLP markers assigned to chromosome C8 of *B. oleracea* (Carlier et al., 2011), with the syntenic region at the top arm of *A. thaliana*’s chromosome 1 (Farinhó et al., 2007). Similarly, Singh et al. (2012) mapped a single dominant R gene, Ppa3 in *B. oleracea* through molecular markers. The map-based cloning of resistant genes to *H. parasitica* from *A. thaliana* was done to encode for TIR- and CC-NBS-LRR categories viz. as RPP5 (Parker et al., 1997), RPP8 (McDowell et al., 1998), RPP1 (Botella et al., 1998), RPP13 (Bittner-Eddy et al., 2000), RPP4 (Van Der Biezen et al., 2002), and RPP2A/RPP2B (Sinapidou et al., 2004). The white rust-resistant genes RAC1 and RAC3 were found closely associated with downy mildew resistant genes RPP9 and RPP8 in *A. thaliana*, respectively (Borhan et al., 2001). A major gene RPP31 for the adult stage resistance against downy mildew pathogen was genetically mapped *in A. thaliana* on chromosome 5 (McDowell et al., 2005). The mutation on the RPP-non-specific locus called EDS1 that is required for the proper functioning of RPP genes revealed enhanced susceptibility in *A. thaliana* (Parker et al., 1996). A large number of R genes have been mapped or cloned in *A. thaliana* and the orthologous genes can be searched in other Brassicaceae members through genome sequence comparisons (Yu et al., 2014) and pan-genome analysis (Golicz et al., 2016). The cDNA clones (Bcchi and BcAF) were isolated and characterized for their role in plant defense mechanisms from *B. rapa* ssp. chinensis L. cv. Suzhouquing against downy mildew pathogen. These clones were translated into protein products and found to be homologous to plant chitinases and defensins. The study reveals their involvement in plant resistance upon infection by a fungal pathogen (Chen et al., 2008). Yu et al. (2011) identified two SSRs (kbrb006c05-2 and kbrb058m10-1) and one Sequence Characterized Amplified Region-SCAR (SCK14-825) marker closely linked to downy mildew resistant QTL (BrDW) on chromosome A8 and used them for MAS in *Brassica rapa* ssp. *pekinensis.* A further extension to this map, a total of four major QTLs (sBrDM8, yBrDM8, rBrDM8, and hBrDM8) identical to BraDM were mapped to A08 through three SNP markers (A08-709, A08-028, and A08-018) for resistance at the seedling, young plant, rosette, and heading stages (Yu et al., 2016). Lucas et al. (1988) have identified a single dominant gene conferred resistance for downy mildew pathogen in *B. napus*. However, the genetic background and environment could influence the phenotypic expression of resistance. The DMR6 gene is responsible for susceptibility in *A. thaliana* plants on infection by downy mildew pathogen. However, the mutants for this gene have lost the susceptibility for *H. parasitica*. The susceptibility of DMR6 mutant *A. thaliana* can be restored by two closely related gene DLO1 and DLO2 (Zeilmaker et al., 2015).

### Powdery Mildew

The powdery mildew of Brassicaceae is caused by biotrophic fungal pathogen *Erysiphe cruciferarum* Opiz ex L. Junell. The pathogen was reported from several parts of the world including Australia, China, Europe, the Former Soviet Union, India, Japan, Korea, South Africa (Kaur et al., 2008; Farr and Rossman, 2013; Kim et al., 2013) in dry-warm weather conditions with low relative humidity favoring the pathogen growth. Besides *B. juncea*, the powdery mildew pathogen can also infect wild members of Brassicaceae such as *Camelina sativa* and *Sinapis arvensis* (Vellios et al., 2017). The pathogen can infect all above-ground plant parts which get covered with dense cottony growth leading to premature leaf fall. The pathogen produces dark-colored cleistothecia on the plant surface, spreading secondary infection, i.e., polycyclic disease. The powdery mildew pathogen can cause upto 17% yield losses in India at harvest (Dange et al., 2002). Tonguc and Griffiths (2004) developed interspecific hybrids and backcross progeny from *B. carinata* and *B. oleracea* cultivars through embryo rescue to transfer resistance against powdery mildew pathogen. The interspecific hybrids tested with powdery mildew under greenhouse conditions were found to be completely resistant. However, only 38% of the first backcross progeny showed a resistant response against the disease. Frye and Innes (1998) have identified an Arabidopsis mutant that displays enhanced disease resistance for powdery mildew caused by *Erysiphe cichoracearum*. The *edr1* mutant does not constitutively express the pathogenesis-related genes *PR-1*, *BGL2*, or *PR-5.* The *edr1* mutation is recessive and maps to chromosome 1 between molecular markers *ATEAT1* and *NCC1.* It was speculated that the *edr1* mutation derepresses multiple defense responses, making them more easily induced by virulent pathogens. However, Vogel and Somerville (2000) identified 20 recessive mutants of *A. thaliana* that inhibit the normal growth of powdery mildew pathogen (*E. cichoracearum*). They concluded that resistance for powdery mildew is not simply due to constitutive activation of the salicylic acid or ethylene and jasmonic acid-dependent defense pathways, but because the mutants did not constitutively accumulate elevated levels of PR1 or PDF1.2 mRNA.

Li et al. (2016) studied multiple evolutionary events involved in maintaining homologous resistance genes in *B. napus* conferring broad-spectrum resistance for powdery mildew pathogen. One and three genes homologous to RPW8 were present in *B. rapa* and *B. oleracea*, respectively. There should be seven homologs of RPW8 in *B. napus*. They found that the copy of *B. oleracea* resistant genes was highly conserved, while the *B. rapa* homolog was variable in the *B. napus* genome possibly due to gene loss, point mutation, insertion, deletion, and intragenic recombination. Nanjundan et al. (2020) evaluated 1020 *B. juncea* accessions against *E. cruciferarum* under natural hot spot conditions. They have identified only one accession (RDV29) consistently resistant for 5 years. This line was used for hybridization with a highly susceptible line (RSEJ775) to obtain filial and backcross populations. The study revealed that resistance was governed by two different semi-dominant genes. Adam and Somerville (1996) used six *A. thaliana* accessions to identify resistant loci for powdery mildew pathogen (*E. cichoracearum*). Their study revealed that out of six, five accessions conferred resistance through a single locus and all were independent and the another one accession have resistance from two unlinked loci. Xiao et al. (1997) identified two independent segregating dominant loci (RPW6 and RPW7) on chromosome 5 and 3 in *A. thaliana* for resistance to powder mildew. The majority of R genes characterized in *A. thaliana* for powdery mildew disease belong to the nucleotide-binding site and C-terminal leucine-rich-repeats (NB-LRRs) and a less prevalent N-terminal transmembrane domain and a coiled-coil motif superfamily. These genes were identified as NDR1, EDS1, PAD4, NPR1, EDS5, RAR1, SGT1b, RPW8, PBS3, COI1, EIN2, and EDR1 (Xiao et al., 2005). However, the transgenic form of *Nicotiana tabacum* and *N*. *benthamiana* developed with RPW8.1 and RPW8.2 genes confers higher resistance against *E. orontii*, *O. lycopersici*, and *E. cichoracearum* but failed to provide resistance to *Lycopersicum esculentum*. Thus, these genes could be used to develop transgenics in other families to provide resistance against powdery mildew disease (Xiao et al., 2003). Alkooranee et al. (2015) investigated the mechanism of systemic resistance induced by *T. harzianum* (TH12) or its cell-free culture filtrate in *B. napus* and *R. alboglabra* to powdery mildew pathogen. The results of the study revealed that the pathogen failed to develop colonies on *R. alboglabra* leaves even after 10 days of inoculation while *B. napus* leaves have fungal colonies after 6 days of inoculation. The expression of PR-1 and PR-2 levels increased in *E. cruciferarum* infected leaves but decreased in the TH12 treated leaves. However, the expression of PR-3 and PDF1.2 is decreased in *E. cruciferarum* infected plants whereas it was increased when treated with TH12 suggesting that TH12 can be used for improving resistance to powdery mildew in hosts.

### Blackleg

The blackleg, also known as phoma stem canker, is one of the most devastating diseases of Brassicaceae family that limits oilseed production worldwide such as in Australia, Canada, and Europe (Zhang and Fernando, 2018). The disease is causing by two coexisting fungal pathogens *Leptosphaeria maculans* (Desm.) Ces et de Not. [anamorph: *Phoma lingam* (Tode: Fr.) Desm.] and *L. biglobosa* sp. nov., Shoemaker & Brun which belongs order Ascomycota (Shoemaker and Brun, 2001). However, the former pathogen causes severe damage to crops than the latter (Petrie, 1978; Rouxel and Balesdent, 2005; Vincenot et al., 2008; Dilmaghani et al., 2009). The strains of *L. maculans* have been categorized into two groups according to pathogenesis, i.e., the strains that causes stem cankers on canola named aggressive, virulent, or “A” group and that do not cause cankers on canola named non-aggressive, avirulent, or “B” group (Howlett et al., 2001). The ascospores are the primary source of disease formed in pseudothecia, produced on host plant residues of the former crop season, and survive up to 5 years on residues (Petrie, 1986, 1995; West et al., 2001; Aubertot et al., 2006). The successful infection leads to the formation of small light green to pale color lesions without margins in the case of *L. maculans* and *L. biglobosa* formed dark brown or gray lesions with distinct dark margin. The asexual fruiting bodies (pycnidia) of the pathogen are formed as small dark spots on leaves and stem which contains pycnidiospores that transmitted through water splashes (Kaczmarek and Jêdryczka, 2011). The pathogens causes severe damage to host plants by girdling and lodging stems (Khangura and Barbetti, 2001). However, the pathogens can infect other parts of host plants such as roots and siliquae.

The commercial varieties of *B. napus* introgressed by resistant genes for blackleg from *B. rapa* subsp. s*ylvestris* lost their ability within three years possibly due to the rapid increase in the frequency of *L. maculans* isolates (Sprague et al., 2006b). Sjodin and Glimelius (1988) screened 96 accessions of the Brassicaceae family at cotyledon, seedling, and adult plant stages against seventeen isolates of *L. maculans* to find out the resistant source for blackleg disease. Out of all tested accessions, only five accessions of *B. juncea*, two of *B. nigra*, and two of *B. carinata* were found resistant to the disease and all contain B genome (Barret et al., 1998). In another study, Dixelius and Wahlberg (1999) analyzed asymmetric progeny of *B. napus* with three B genome donors (*B. nigra*, *B. juncea*, and *B. carinata*) for the presence of resistance to blackleg disease. They have identified a total of four co-segregating RFLP markers for cotyledon and adult-leaf resistance which was associated with six loci present on linkage groups 2, 5, and 8. A triplicate region in the B- genome had preserved the resistant loci in all three species. Fredua-Agyeman et al. (2014) provided evidence for the B3 chromosome of the B genome to carry resistant genes and confirmed that the entire chromosome was associated with blackleg resistance. Thus, *B. napus* suffer more severely from the blackleg disease than *B. juncea* (Marcroft et al., 2002). However, a large number of attempts were made to transfer blackleg resistance into *B. napus* from members of the Brassicaceae family (Chèvre et al., 2003; Winter et al., 2003; Gaebelein et al., 2019) and stabilized (Chevre et al., 1997; Barret et al., 1998; Chèvre et al., 2008). The *B. nigra* monosomic lines for chromosome 4 in *B. napus* have the same level of resistance as in *B. nigra* (Chevre et al., 1996). The resistance to blackleg disease is governed by a polygenic system and present in a clustered manner. However, the resistance provided by the B genome remains constant throughout the life cycle of the host plant (Rimmer and Van-den Berg, 1992; Chèvre et al., 2003). The F_2_ and backcross population of two different cultivars of *B. juncea* (resistant- AC Vulcan and susceptible- UM3132) was segregated for blackleg resistance which suggested that the resistance was controlled by two independent dominant and recessive genes positioned on linkage group J13 and J18, respectively (Christianson et al., 2006). The NILs developed from the asymmetric somatic hybrids of *B. napus* with *B. juncea* and B. nigra showed that one single dominant allele and two independent loci govern adult plant stage resistance to blackleg in *B. napus–B. juncea* and *B. napus–B. nigra* lines, respectively (Dixelius, 1999). The B- genome introgression lines of *B. napus* confirmed the inheritance of recessive resistant gene, rjlm2 from *B. juncea* for the blackleg disease at the cotyledon stage (Saal et al., 2004).

In contrast to A- or B- genome crops where a large number of resistant genes have been identified, few resistant genes were also identified on C- genome crops (Hossain et al., 2020). During a study, *Rlm1*, *Rlm2*, *Rlm3*, *Rlm4*, *Rlm9*, *RlmS*, *LepR1*, and *LepR2* genes were reported to present in Canadian canola varieties (Zhang et al., 2016) and also a large number of avirulence (Avr) genes (∼14) in corresponding *L. maculans* pathogen and cloned few of them (Liban et al., 2016). The mapping and cloning of an avirulence gene, AvrLmJ1, from *L. maculans* confers avirulence to *B. juncea* cultivars (Van-de Wouw et al., 2014). To date, at least sixteen resistant genes for blackleg were mapped in *B. napus* and allied Brassica species. Out of these resistant genes, many were present in an association of others or clustered together (Delourme et al., 2004). The inheritance of resistance against blackleg in *B. juncea* was breached in the recent past by newly evolving strains of *L. maculans* in Australia (Elliott et al., 2015) but the breakdown of *Rlm3* resistance was not reported until recently in Canadian canola varieties (Zhang and Fernando, 2018). However, some other reports indicated that *Rlm1, Rlm3, Rlm6, Rlm7, LepR1*, and *LepR3* lost their effectiveness in the field against the blackleg pathogen (Rouxel et al., 2003; Sprague et al., 2006a,b; Brun et al., 2010; Winter and Koopmann, 2016; Zhang et al., 2016; Van-de Wouw et al., 2017). In a study, the blackleg resistant gene *Rlm2* was located on chromosome A10 of the *B. napus* cv. Glacier with the help of tightly linked microsatellites (sR1448, sN8502, sN1982, and sN8474). The gene was localized to a 5.8 cM interval corresponding to approximately 873 kb of the *B. napus* chromosome A10 (Larkan et al., 2014).

The *Rlm4* quantitative trait locus was characterized for harboring 18 candidate resistant genes (BLR1–BLR18) for blackleg in *B. napus* through NGS (Tollenaere et al., 2012). The LmR1 locus controls seedling resistance for blackleg in *B. napus* cv. Shiralee was positioned on linkage group N7, find out after fine mapping of 2500 backcross lines. A total of three microsatellites were found associated with resistance and co-segregated with this gene. However, an additional seedling resistance gene, ClmR1, was identified in the same region of LmR1 (Mayerhofer et al., 2005). Yu et al. (2005) have identified and mapped two resistant alleles, *LepR1* and *LepR2*, for blackleg resistance in *B. napus* lines introgressed from *B. rapa* subsp. *sylvestris* that were located on linkage group N2 and N10, respectively. In addition to both these, they have mapped a third resistant gene *LepR3* by microsatellite markers on linkage group N10 at below the *LepR2* gene in *B. napus* “Surpass 400” (Yu et al., 2008). Raman et al. (2018) assessed quantitative resistance (QR) for blackleg in DH lines of Darmor-bzh/Yudal (DYDH) population in three successive years and identified a total of 27 significant QTLs on 12 different chromosomes. Out of which, seven were repeatedly present on chromosomes A02, A07, A09, A10, C01, and C09 in all experiments. They have identified eight stable QTLs for blackleg in three diverse growing conditions of Australia, France, and the United Kingdom. In another study, genome-wide association analysis found extensive variations in resistance to blackleg at the adult plant stage in *B. napus*. A total of 59 statistically significant SNPs were identified on seventeen chromosomes of *B. napus* genome that were responsible for variations to blackleg resistance (Raman et al., 2020). Huang et al. (2019) identified a total of five QTLs on linkage groups A02, A03, A10, C01, and C09 for resistance to *L. maculans* growth on oilseed rape DY (“Darmor-*bzh*” × “Yudal”). Mapping population contributed about 35 percent phenotypic variations.

## Bottlenecks in Identification of Resistance Sources

### Genome Complexity in *B. juncea*

The diploid ancestral species (*B. rapa* and *B. nigra*) of *B. juncea* have evolved from a common ancestor followed by ancient genome triplication along with structural and numerical changes about 7.9–14.6 million years ago (Lysak et al., 2005). *B. juncea* developed through the spontaneous hybridization of these two ancestral Brassica species by combining the genome to give rise to an allotetraploid species. This event was followed by a natural chromosome doubling. It is suggested that *B. rapa* was a cytoplasmic donor in *B. juncea* development process (Banga et al., 1983; Warwick and Black, 1991; Pradhan et al., 1992; Prakash et al., 2009). The crop was diversified into vegetable and oil-producing subvarieties and cultivation was started about 6000–7000 years ago in China (Yang et al., 2018) and 2300 BC in India (Prakash and Hinata, 1980). However, it was assumed that *B. juncea* formed about 0.039–0.055 million years ago (mya) while *B. napus* formed about 0.038–0.051 mya, slightly after the formation of *B. juncea*. *B. juncea* was estimated by flow cytometry to possess a 922 Mb genome size. The *B. juncea* genome has 316.1 Mb of repetitive sequence, out of which 131.2 Mb are from *B. rapa* and 216.5 Mb from *B. nigra* genomes. During the speciation process, it was estimated that a total of 562 and 545 genes from *B. rapa* and *B. nigra* subgenomes of *B. juncea* were lost, respectively, as compared to their common ancestral genomes. This number is higher than the gene loss estimated in *B. napus* subgenomes (BnaA and BnaC) as compared to their common ancestral genomes. However, the number of genes lost in *B. juncea* and *B. napus* was consistent since their formation. The A subgenome of *B. juncea* and *B. napus* had divergent origins. It was discovered that A subgenome of *B. juncea* might be derived from Asian *B. rapa* ssp. *tricolaris*, while the subgenome A of *B. napus* might be derived from European *B. rapa* ssp. rapa. Thus, both A subgenomes of allotetraploids had independent geographical origins. It was also discovered that the chromosomal regions of *B. juncea* had gone through various rearrangements and formed the current species contributing to gene duplications and losses. The polyploidy nature of *B. juncea* played an essential role in genome speciation and plasticity (Yang et al., 2016). The A subgenome of *B. juncea* remained intact while the B subgenome has changed considerably. However, the B subgenome in *B. carinata* is unchanged during evaluation (Prakash et al., 2009). *B. rapa* possesses a rich genetic diversity with various desirable agronomic traits, i.e., rapid growth and tolerance to nutrient-deficient soil and low temperatures (Franks, 2011; Waalen et al., 2011; Ahmed et al., 2012).

The cultivated *B. juncea* varieties have very lower genetic diversity due to the unidirectional selection force for yield characteristics. This is also evident from recently developed genome assemblies of *B. Juncea* var. tumida (Yang et al., 2016), BjVaruna (Paritosh et al., 2020), and it’s parent species *B. rapa* (Wang et al., 2011; Cai et al., 2017), and *B. nigra* (Yang et al., 2016). However, the complete genome sequences were not developed during the assembling process which leads to deletion (∼10–20%) of sequences containing potential R genes. Contrastingly, the artificial *B. juncea* was synthesized by combining the Asian oil crop *B. rapa* (A^r^A^r^) and the B^c^ subgenome from the African oil crop *B. carinata* (B^c^B^c^C^c^C^c^) and synthesized allohexaploid (A^r^A^r^B^c^B^c^C^c^C^c^), crossed with traditional *B. juncea* to generate pentaploid F_1_ hybrids (A^r^A^j^B^c^B^j^C^c^), with subsequent self-pollination to obtain newly synthesized *B. juncea* (A^r/j^A^r^/^j^B^c/j^B^c^/^j^). The genetically stable new type of *B. juncea* population was obtained at the F_6_ generation retaining good fertility and rich genetic diversity while being distinct from the traditional *B. juncea*. This newly developed *B. juncea* had more than half a modified genome due to exotic introgressions and novel variations in gene copies, numbers, and sequences (Wei et al., 2016). This innovatively developed *B. juncea* can be utilized for the identification of novel R genes and the improvement of genetic base.

The R genes controlling vertical resistance and function of the host against a specific race or strain of the pathogen can be altered easily for resistance-breeding, compared to polygenic host resistance which involves the collective action of more than one gene. However, the identification of R genes in *B. juncea* is challenging due to complex genomic structures. The *B. juncea* genome was identified to contain a total of 289 NBS-LRR type resistance genes, more than its diploid progenitors (*B. rapa* and *B. nigra*). A total of 4 and 7 QTLs for the white rust and blackleg resistance in *B. juncea* respectively, were identified. The white rust resistant locus AcB1-A04.1 was present on the linkage group A04 and B01, while another locus AcB1-A05.1 was present on the same linkage group on an overlapping position at A05 with a similar copy at B06. Similarly, blackleg-resistant quantitative loci-PhR2 were identified on the linkage groups A03 and B03, and another QTL, RLM6, identified on three linkage groups (A07, A09, B01). However, PhR2 and LMJR1 resistant loci for blackleg were present on linkage group B03. This study concluded that the *B. juncea* genome has duplicated sequences (Inturrisi, 2018). The different resistant genes or QTLs were clustered together in the *B. juncea* genome. The clustering effect of resistant gene/QTL for white rust and blackleg diseases directs to the complexity of the *B. juncea* genome. The gene clustering is commonly found in plant genomes to adopt a quick response against pathogens through recombination incidents (Hulbert et al., 2001; Meyers et al., 2003). The information regarding resistant QTLs/genes for major diseases is useful to develop durable resistant cultivars effective against a wide range of pathogen strains or races. The overlapping, clustering, or allele copies of R genes can cause problems in the identification of candidate resistant genes as many such regions may not be a part of sequenced reference genome assemblies. The dependency on a single reference genome assembly to identify resistant genes can obstruct efforts in the identification and use of such R genes. All possible future endeavors should add innovative technologies for the sequencing of target chromosomes or regions.

### Genetic Variations in Pathogens

The genome complexity of *B. juncea* in terms of resistant genes and resistance levels is also variable in different hosts for the major disease-causing pathogens, as discussed before. It has been noticed frequently that variable disease symptoms were produced when infected by different isolates/strains obtained from a different source or geographical region of the same source. These variable symptoms could be due to genetic variations within the host genotypes and/or within the pathogen. The leaf blight or black spot pathogen of *B. juncea* has very high genetic diversity all over the world. *A. brassicae* has three races viz. RM-1, RM-2, and V-3 in India which are virulent on the rapeseed-mustard group of crops (Saharan and Kadian, 1983). However, in different investigations on the diversity of *A. brassicae*, Awasthi and Kolte (1989), Kolte et al. (1991), Kumar et al. (2003), and Sangwan and Mehta (2007) identified various races from Indian regions. In recent studies on the identification of genetic variability of *A. brassicae* in 32 isolates collected from Himachal Pradesh (India), a total of seven pathogenic races were identified designated as Abr1 to Abr7 (Kumar et al., 2014). The races/pathotypes identified by various studies presented a highly virulent to non-virulent response on screening with different Brassica crops. Sharma and Tewari (1998) collected isolates of *A. brassicae* from different geographical regions of the world (India, Canada, France, Costa-Rica, England, and Poland) and analyzed them with RAPD and RFLP markers. Having tested a total of 20 decamer primers of arbitrary nucleotide sequences for PCR amplification of *A. brassicae* genomic DNA, only five primers with amplified genomic DNA from 20 isolates of *A. brassicae* were selected and classified into four groups. However, the variations between isolates collected from intra-geographical regions were very less apparent. Kaur et al. (2007) evaluated 322 isolations of *A. brassicae* collected from a wide geographic area of north-west India. Out of these, 114 were identified as pathogenic and all were categorized under seven broad groups based on morphological characters showing a wide range of diversity. Goyal et al. (2013) characterized 13 isolates of *A. brassicae* collected from different parts of India through 100 RAPD decamer primers and found genetic variability among the isolates at the genomic level, but not in the highly conserved regions of the genome of *A. brassicae*. Aneja et al. (2014) studied genetic diversity through RAPD markers in 32 isolates of *A. brassicae* collected from eight north Indian states and found a total of 57–78% genetic diversity within all isolates with no correlation established. They determined the genetic relationship among 32 *A. brassicae* isolates by UPGMA revealing the clustering into four major classes that were further subdivided into nine subgroups. Rajarammohan et al. (2019a) identified 460 *A. brassicae* specific genes which included many secreted proteins and effectors. They also identified gene clusters responsible for the production of pathogenicity specific Destruxin-B (a cyclic depsipeptide). However, in the recent past, a chromosome level about a complete genome assembly of *A. brassicae* was developed that could be useful to uncover the *B. juncea–A. brassicae* pathosystem related genes (Rajarammohan et al., 2019b).

The races of white rust (*A. candida*) isolated from four Brassicaceae members behaved as four independent races in Saskatchewan (Canada). These races were evaluated for their virulence in various Brassica members collected from different parts of the world. The results showed that certain crop species were susceptible to specific race but not to all races. However, some accessions of Brassica show resistance to some of these races, but not all (Petrie, 1988). Jouet et al. (2019) analyzed 85 isolates of *A. candida* for genetic diversity collected from many European countries infecting various Brassicaceae hosts.

### Impact of Environmental Conditions on Disease Development

Environmental factors play a key role in pathogen inoculum distribution, host–pathogen interaction, disease development, and its severity. These factors included soil pH, moisture, and temperature, available soil nutrients, environmental temperature, relative humidity (RH), and inoculums dispersal conditions. All these environmental factors directly influence the host-resistance status by affecting pathogen growth and reproduction potential. The sclerotia developed by *S. sclerotiorum* at the end of each successful disease cycle can be germinated between 10 and 15°C temperatures, however above this temperature the germination is affected severely (Jones and Gray, 1973). The carpogenic germination of sclerotia was affected by variable soil moistures and relative humidity (Hao et al., 2003; Matheron and Porchas, 2005). The development of the stem rot lesion is favored by moist conditions and a temperature range of 20–25°C. However, the lesion growth will interrupt under dry and warm conditions but may resume when favorable conditions appeared again (Canola Council of Canada, 2020). The sporulation in *A. brassicae* on naturally infected leaves of oilseed rape needs a relative humidity of 91.5% or above with temperature ranging between 18 and 24°C. However, the sporulation is inhibited just above the 24°C temperatures (Jones and Phelps, 1989). For successful infection, a minimum wetness period of 4 h is required by *A. brassicae* at 18°C and disease severity increases with increasing wetness period up to 12 h. The disease severity also increases with increasing inoculum concentration from 80 to 660 spores ml^–1^ with increasing leaf age from 4 days (Hong and Fitt, 1995). The severity of white rust on *B. juncea* leaves was favored by >40% afternoon (minimum) RH, >97 mornings (maximum) RH with 16–24°C daily temperatures. However, the staghead formation was influenced positively between temperature ranges of 20–29°C and >97% morning RH (Chattopadhyay et al., 2011). In contrast, the powdery mildew pathogen of *B. juncea* needs warmer (24–30°C) environmental conditions and low relative humidity (∼65 RH) for successful disease establishment and conidial dispersion (Desai et al., 2004).

### Re-emergence of Virulent Pathogen Races and Host–Pathogen Interactions

The hybridization between two races/isolates of the pathogen is recognized as a major force in the evolution of new races or adaptation of exiting plant pathogenic race (Brasier, 2000, 2001). The global agriculture commodity trade moves pathogens and hosts around the world and has played a key role in the emergence of new races or diseases (Brown and Hovmoller, 2002). The hybrid race or pathogen can be formed by sexual hybridization between parental species or fusion between hyphae or vegetative cells. The fusion of vegetative cells can be followed by parasexual reproduction where mitotic crossing over generates recombinant cells or new races (Schardl and Craven, 2003). The cultivation of resistant varieties containing the R gene for vertical resistance against a particular pathogen race or isolate exerts selection pressure on the pathogen that is better adapted to breach the host defense system. The resistant crops cultivated continuously in a particular field should be avoided due to the direct exposure to virulent strains of pathogens that can overcome the host resistance. Thus, the vertical host resistance may effectively work against a particular pathogen race for a short period and lose its effectiveness after 3–5 crop seasons. A new race of *A. candida* was identified on *Lepidium latifolium* (pepperweed) in California which was unable to infect fifteen host differentials of the Brassicaceae family. Thus, the host range information of any particular pathogen is important to identify new races (Koike et al., 2011).

The host resistance mechanism is dependent on a particular host pathosystem as a single major gene or a group of genes involved in the defense mechanism. In the *B. juncea*–*S. sclerotiorum* pathosystem, a highly resistant response was recorded for stem rot in cotyledon and stem assays. The expression of differential resistance response in introgression lines showed quantitative trait inheritance governed by more than one gene which works cumulatively against *S. sclerotiorum*. In this pathosystem, the resistance at seedling or adult plant stage and in cotyledon, leaf, or stem were expressed independently. The resistance shown by a particular resistant host is also dependent on the pathogen aggressiveness or virulence. *A. thaliana*–*A. brassicae* pathosystem has a quantitative inheritance for resistance by major independent loci. There were common and population-specific QTLs and also a chance of different genes governing resistance to the pathogen. Out of these, one QTL expressed about 50% variation in disease resistance with the genes present within probably contributing to resistance even in heterogeneous conditions (Rajarammohan et al., 2017). In contrast, the genetic mechanism of host resistance in *B. juncea* is different for the white rust pathogen. The host resistance was reported to be governed by a single locus on LG A4, LG A5, and LG A6 in Heera, Donskaja-IV, and Tumida lines of *B. juncea*, respectively. However, the resistant genes conferring resistance in these loci were found closely related to R gene BjWRR1 (Bhayana et al., 2020). It will be beneficial for molecular studies to identify the hosts conferring resistance against a broadspectrum of pathogens i.e. hosts showing resistance to all races or isolates of the pathogen.

The use of different disease scoring parameters has also created troubles in the identification of true resistant lines resulting in misidentification of R genes. Atleast threescoring systems are used in Alternaria blight disease screening (Conn et al., 1990; Vishwanath et al., 1999; Meena et al., 2016; Ayuke et al., 2017). Similarly in *S. sclerotiorum*-*B. juncea* pathosystems, there are two scoring systems to identify resistant crop plants, and different plant parts and host-age used in the evaluation of resistance (Kumari and Singh, 2019; Gupta et al., 2020). Thus, it is necessary to develop a robust screening method for each pathosystem to identify the same or different races/pathotypes, as well as host resistance status.

## Strategies for Brassica Improvement

### Identification and Introgression of Novel R Genes

**FIGURE 2:**
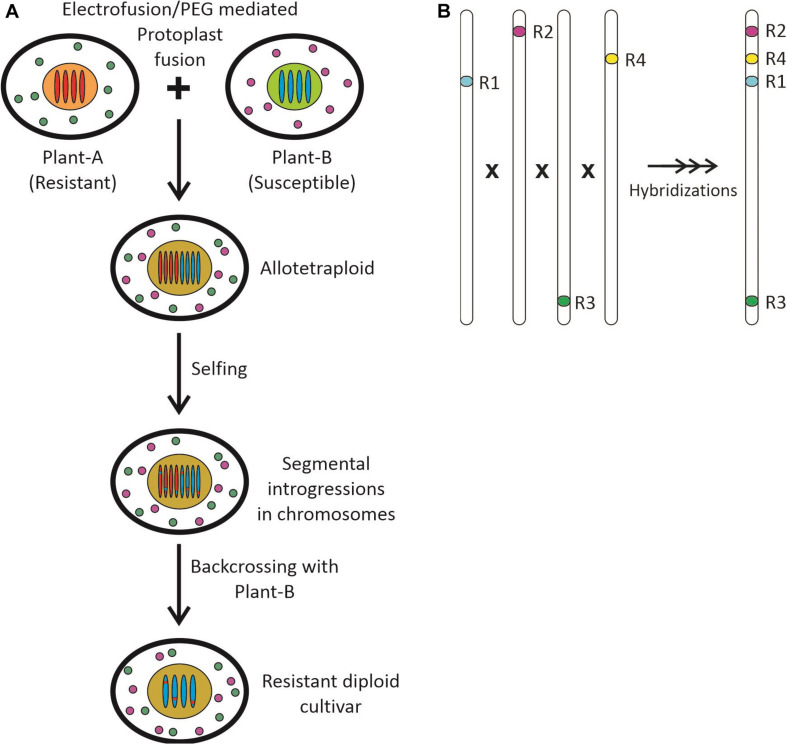
**(A)** The development of resistant segmental introgression lines from susceptible cultivars through PEG mediated protoplast fusion and subsequent backcrossing; **(B)** The gene pyramiding approach by wide hybridization and selection.

The Brassica coenospecies or wild members of the Brassicaceae family have a high degree of resistance against *S. sclerotiorum* such as *B. fruticulosa*, *S. alba*, *B. incana*, *C. sativa*, *D. tenuisiliqua*, *E. abyssinicum*, and *E. cardminoides*. These species were used to introgress resistance into *B. juncea* and *B. napus* through somatic hybrid production through PEG mediated protoplast fusion, followed by backcrossing with the cultivated parent. The introgression lines possessing segmental or chromosomal introgression demonstrated promising resistance against stem rot disease (Figure 2A). However, the screening experiments should be performed with virulent races/isolates and introgression lines through robust screening assays to identify true resistant breeding lines. Next-generation sequencing will help in developing a near-complete genome assembly of these lines serving as a good source in the identification of R genes through comparative genome analysis. This approach will help broaden the genetic base by introgression of novel R genes into Brassica lines.

In recent years, a large number of candidate R genes have been identified for various diseases of the mustard plant (Table 1). The information regarding these gene sequences will enhance the cloning capacity and also be transferred into cultivated species to develop resistant transgenic plants. However, there has been very little progress in the cloning of R genes. The use of completely characterized antifungal genes to develop transgenic Brassica lines will be beneficial to confer resistance against a range of pathogens. The hevein producing gene was used to develop transgenic Brassica which displayed enhanced resistance against leaf blight pathogen (Kanrar et al., 2002). There are some more examples for use of antifungal genes to develop transgenic Brassica for improving resistance such as barley antifungal genes class II chitinase (AAA56786) and type I ribosome-inactivating protein (RIP; AAA32951), and tomato glucanase. All these provide higher resistance to Brassica lines against *A. brassicae* (Mondal et al., 2003, 2007). The *B. juncea* transgenic lines developed with antimicrobial gene msrA1 have shown upto 85 and 71.5% disease reduction caused by *A. brassicae* and *S. sclerotiorum*, respectively (Rustagi et al., 2014). Moreover, BjNPR1 transgenic lines of *B. juncea* showed improved resistance to *A. brassicae* and *E. cruciferarum* as there was an impediment in symptom development and reduced disease severity than non-transgenic plants (Ali et al., 2017). The chickpea lectin transgenic of *B. juncea* conferred resistance against *A. brassicae* showing upto 60% disease reduction with additional resistance against salinity and drought stresses (Kumar et al., 2015). The transfer of resistant genes from non-host plants conferring resistance to biotic diseases into the high-yielding susceptible host is a promising strategy toward the development of resistant Brassica varieties. The use of non-host genes into host plants has opened a new way of crop improvement and protect susceptible lines that lack resistance sources.

### Resistance Breeding and Development of Pre-breeding Resistant Lines

Incessant efforts are being put in to introgress resistance into cultivated *B. juncea* lines by breeding with resistant allied wild genera or species. *B. incana* was identified to possess a high degree resistance against stem rot disease which can be transferred into cultivated susceptible *B. oleracea* var. *alboglabra*. The candidate R genes for SSR were identified on the C09 chromosome for leaf and stem rot which belongs to CC-NBS-LRR class (Mei et al., 2013). The *B. napus* lines evaluated for SSR resistance showed many putative QTLs on C02, C04, C06, C08, C09, A02, A03, and A08 chromosomes (Wu et al., 2013, 2016, 2019; Gyawali et al., 2016). The SSR resistant loci working cumulatively, thus pyramiding of resistant loci, could efficiently protect against a wide range of *S. sclerotiorum* races/isolates (Figure 2B). The *B. juncea* introgression lines carrying chromosomal segments of wild *B. fruticulosa* showed a resistant response against SSR and marker-trait association (Rana et al., 2017).

The related wild species of *B. juncea* was reported to possess genes conferring resistance against Alternaria blight disease that can be used for resistance breeding programs (Fatima et al., 2019). The advanced biotechnological approaches such as tissue culture and genome transformation have also been used for developing resistant Brassica lines. An *in vitro* ovary and ovule culture was attempted to transfer resistance against *A. brassicae* from *S. alba* to *B. napus* (Chevre et al., 1994) and *E. cardaminoides* to *B. oleracea* var. *alboglabra* (Mohanty et al., 2009). Agnihotri et al. (1991) obtained interspecific hybrids of *B. campestris* (*B. rapa*) and *B. spinescens* through ovary, ovule, and embryo rescue conferring resistance for Alternaria blight. Moreover, the somatic hybridization (protoplast fusion) technique has been used for transferring resistance from *S. alba* to *B. napus* (Primard et al., 1988; Wang et al., 2005), *B. juncea* (Gaikwad et al., 1996; Kumari et al., 2018, 2020c; Kumari and Bhat, 2019), *B. oleracea* (Hansen and Earle, 1997), *B. carinata* to *B. juncea* (Sharma and Singh, 1992), *B. nigra* to *B. oleracea* (Jourdan and Salazar, 1993), and *B. spinescens* to *B. juncea* (Kirti et al., 1991). There were some attempts made to introduce somaclonal variations to incorporate disease tolerance for Alternaria blight through mutagenesis (Verma and Rai, 1980; Agnihotri et al., 2009). The introgression lines developed for Alternaria blight resistance were used in the resistant breeding programs.

The *B. juncea* genome confers a good resistance source to white rust disease caused by *A. candida*. The marker-trait association was established in RAPD markers and white rust resistance that can be used for marker-assisted resistant breeding programs (Prabhu et al., 1998). The potential resistance genes against white rust come from the B genome of *B. nigra* and *B. juncea* (Delwiche and Williams, 1974; Massand et al., 2010). Moreover, some other lines of *B. rapa*, *B. napus*, *B. carinata*, and *A. thaliana* also possess genetic resistance to the white rust pathogen. However, the monogenic resistance was found effective against a particular race of *A. candida*. Thus, the resistant lines can be selected for resistant breeding programs according to the pathogenic race that is prevalent in a particular region or for resistant gene pyramiding program to develop lines governing resistance for more than one pathogenic race. In the breeding programs, the selection of parents who carry the R genes is important since their function may be masked in the newly evolved genome of offsprings due to an epistatic interaction (Werner et al., 2007). However, this problem can be managed successfully through the genome editing technique, the CRISPR-Cas (Pickar-Oliver and Gersbach, 2019). To select parent genotypes for resistant breeding, the field analysis of pathogen isolates for their virulent/avirulent genes should be considered. To select parent genotypes for resistant breeding, the field analysis of pathogen isolates for its virulence/avirulence genes should be considered that are adapted for host and identify the genetic variations in the pathogen races that can overcome the host resistance. Furthermore, the consideration of the host R gene is also important as pathogen pressure can affect the performance of R genes (Neik et al., 2017). *B. napus* cv. Surpass 400 was released in the year 2000 as the most resistant cultivar to *L. maculans* carrying a single dominant resistant gene from *B. rapa* subsp. *sylvestris*. However, in 2001 during an experiment, the cv. Surpass 400 and the susceptible cv. Westar were inoculated with pycnidiospore suspension of 18 isolates of *L. maculans*. The inoculated cotyledons of cv. Surpass 400 showed characteristic disease symptoms while susceptible cv. Westar was collapsed. The calculation of disease severity revealed that a total of 54% of cv. Surpass 400 and 100% plants of cv. Westar were susceptible to the disease. This study confirmed the ability of *L. maculans* isolates to overcome the resistance governed by a single dominant gene present in cv. Surpass 400. The information regarding the co-evolution of resistant genes and pathotypes is also important before the selection of a resistant host to develop durable resistant lines (McDonald and Linde, 2002).

### Co-inoculation to Improve the Resistance of Susceptible Brassica Lines

In the *L. maculans*–*B. napus* pathosystem, the *B. napus* cv. Madrigal seedlings were pre-treated with ascospores of *L. biglobosa*, foliar sprays of ASM, or MSB and showed improved resistance against *L. maculans* (Liu et al., 2007). *Pseudomonas syringae* pv. tomato, the causal pathogen of bacterial speck disease in tomato and Arabidopsis induces resistance on pre-inoculation in Chinese cabbage to *Erwinia carotovora* subsp. *carotovora* that causes soft rot disease. It was found that Pst activates both salicylate-dependent and salicylate-independent defense responses in Chinese cabbage (Park et al., 2005). The pre-inoculation of the virulent strain of *H. parasitica* enhanced resistance against *A. candida* in white rust susceptible *B. juncea* host (Singh et al., 2002). The susceptible *B. juncea* cv. PR-15 showed resistance against virulent isolates A and C of *A. brassicae* when pre-treated with avirulent isolate D of *A. brassicae*. However, *A. alternata* failed to induce resistance for *A. brassicae* isolates A and C, rather it induced susceptibility to them (Vishwanath et al., 1999). The pre-inoculation of *B. oleracea* var. italica (Accession: Milady) seedlings with avirulent isolates of *P. parasitica* induced systemic resistance against virulent isolates and reduced disease incidence upto 70% (Monot et al., 2002). In contrast, it was recorded that the successful infection by a pathogen of a susceptible host could enhance the susceptibility of the host to secondary infection even for avirulent pathogens. The host defense system was suppressed by oomycete pathogen *A. candida* in *A. thaliana* and *B. juncea*. A pre-infection of *B. juncea* by *A. candida* can suppress immunity in cotyledons to downy mildew pathogen (*H. parasitica*), however, *B. juncea* still possesses resistance to mildew pathogen. The pre-infection with *A. candida* can successfully suppress a broadspectrum resistance conferred by RPW8 to the two morphotypes of Erysiphe spp. (a powdery mildew pathogen) in *A. thaliana* (Cooper et al., 2008). In the *H. parasitica*–*B. juncea* pathosystems, the pre-inoculation of *A. candida* to *B. juncea* followed by inoculation of avirulent *H. parasitica* increased susceptibility for white rust pathogen. However, the host plant that was resistant to downy mildew was also found to be infected systemically by *H. parasitica*. This work suggested that the host resistance could be determined by pathogen strain, infection sequence, or virulence status of the pathogenic strain (Kaur et al., 2011b). However, the molecular basis of enhanced resistance or susceptibility performance of host plants is still unknown when co-infected by pathogens.

### Defense-Related Secondary Metabolites and Proteins

The plants have developed some alternative defense systems, such as secondary metabolites (Glucosinolate-myrosinase, phytoalexins, and phytoanticipins) and defense-related proteins (chitinases, glucanases, thionins, chitin-binding lectins, ribosome-inactivating proteins) to protect them from pathogens or stressful conditions other than occupying resistant genes (Terras et al., 1993; Kumar, 2017). The production of a large number of secondary metabolites from primary metabolites is induced when plants face biotic or abiotic stresses and they get accumulated in plant cells (Rejeb et al., 2014; Caretto et al., 2015; Narayani and Srivastava, 2018). These secondary metabolites usually belong to one of the three large chemical groups terpenoids, phenolics, and alkaloids. The gossypol, a member of the terpenoid class is produced by *Gossypium hirsutum* and has strong antibacterial and antifungal properties. Additionally, saponins (glycosylated triterpenoids) that are present in the cell membranes have detergent-like properties and disintegrate cell membranes of fungal pathogens. However, some fungal pathogens have saponin-degrading capacity causing diseases like *Botrytis cinerea*, *Fusarium oxysporum*, and *Septoria lycopersici*. The phytoalexins (camalexin, brassinin, and rapalexin A) have antibacterial and antifungal properties that are produced on the pathogenic attack. These are toxic molecules that disturb the metabolism or cellular structure of pathogens but are often toxic to a specific pathogen (Freeman and Beattie, 2008). The glucosinolates are sulfur-containing secondary metabolites synthesized by members of the *Brassicaceae* family. These glucosinolates are hydrolyzed by myrosinase enzyme secreted as a consequence of cell wall lysis (Osbourn, 1996). The reaction by-products are predominantly isothiocyanates (ITCs) having biocidal distinctiveness against a wide range of insects, nematodes, fungal pathogens, bacteria, and weeds (Brown and Morra, 1997; Bednarek, 2012).

The defensins are small cysteine-rich proteins that show broad-spectrum anti-microbial activity and were first isolated from the endosperm of *Hordeum vulgare* and *Triticum aestivum*. They are predominantly characterized in the seeds but can be present in all types of plant tissues (leaves, pods, tubers, fruit, roots, bark, and flowers). The specific mechanisms employed by defensins to inhibit fungal growth are still being characterized, but they appear to act upon molecular targets in the plasma membrane of pathogens. The chitinases and glucanases are enzymes that catalyze the degradation of chitin and glucans, respectively, polymers with a backbone similar to cellulose that is present in the cell walls of true fungi. The *in vitro* analysis has confirmed the anti-fungal activities of these enzymes, and transgenic plants expressing a high concentration of these enzymes exhibit increased resistance to a wide range of pathogens (Freeman and Beattie, 2008). Another class of cysteine-rich anti-fungal proteins is 2S albumin, AFP1, and AFP2 commonly present in members of the Brassicaceae family displaying broad antifungal properties (Terras et al., 1993). The receptor-like protein (RLP) encoded by a Ve1 gene from tomato has fungicidal properties and is transformed successfully into *A. thaliana*. However, very little is known about the RLP signaling in pathogen resistance (Fradin et al., 2011). Thionins are cysteine-rich proteins of low molecular weight (5 kDa) with anti-fungal activities and mainly accumulated in seeds but may be present in stems, roots, or leaves (Bohlmann, 1994; Bohlmann et al., 1998). These are toxic to a broad range of fungal pathogens, presumably attacking the cell membrane to increase their permeability and consequently leading to cell death (Cammue et al., 1992; Terras et al., 1995; Epple et al., 1997; Holtorf et al., 1998; Chan et al., 2005). The transgenics overexpressing thionin genes showed enhanced resistance against phytopathogens (Kanzaki et al., 2002; Khan et al., 2006; Almasia et al., 2008; Hao et al., 2016). A large number of thionin producing genes have been identified in members of the Brassicaceae family such as *Arabidopsis*, *Eutrema*, and *Raphanus* (Hoshikawa et al., 2012). The Kelch domain protein of *Trichoderma harzianum* T34 encoded by the *Thkel1* gene was found to confer salt tolerance and also improve resistance against phytopathogens by induction of Jasmonic acid-mediated systemic resistance and myrosinase activity when overexpressed in transformed *B. napus* (Poveda et al., 2019). It was shown that a pair of *A. thaliana* TIR-NLR proteins, RRS1 and RPS4, function together in disease resistance against different phytopathogen isolates. These dual R proteins also confer resistance to *Ralstonia solanacearum* wilt in transgenic *Brassica* crops (Narusaka et al., 2014). The *in vitro* bioassays demonstrated that ARACIN1 and ARACIN2 peptides have antifungal properties against necrotrophic fungi such as *B. cinerea*, *A. brassicicola*, *Fusarium graminearum*, and *S. sclerotiorum*, and yeast (*Saccharomyces cerevisiae*). Moreover, the transgenic *A. thaliana* plants expressing ARACIN1 were well protected from infections of *B. cinerea* and *A. brassicicola* (Neukermans et al., 2015).

### Characterization of Pathotypes and Pathogen Effectors/Elicitors

The identification of pathogen race or strain is important for the successful management of disease outbreaks in a particular region/country as some resistant genes were identified to work against a particular race or strain. This goal can be achieved by the characterization of pathotypes through genetic markers such as race/strain/isolate specific molecular markers, proteins, and genes. The strains of *A. alternata* can be identified by the comparative analysis of ITS, GAPDH, and RPB2 genetic markers (Zheng et al., 2017). Rajarammohan et al. (2019a) have identified a total of 460 genes specific to *A. brassicae* which were absent in all other Alternaria species. These genes have included 35 secreted protein-coding genes and out which 11 were predicted to be effectors. A large number of these proteins were uncharacterized proteins with no known function. These *A. brassicae* specific genes and effectors could be a potential source for categorization and identification of *A. brassicae* races.

The advanced sequencing technology provides near-complete genome assemblies that serve better in gene predictions and will also provide improved results for structures and functions of effector proteins. The characterization of effector genes/proteins can be a potential identification source for pathotypes (Gibriel et al., 2016). However, the effector candidates characterized functionally to date were below 300 amino acids with ∼4 cysteine residues (Stergiopoulos and de Wit, 2009). These characteristics can not be used as identification criteria because well-characterized effectors lack these properties (Lo Presti et al., 2015; Sperschneider et al., 2015). Therefore, the universal characters of effector candidates such as their secretion and differential expression in crops should be considered (Sperschneider et al., 2015). The effector candidates identified by comparative genome-based studies can be used in R gene identification (Vleeshouwers et al., 2008). The monitoring of the effector candidate diversity of pathotypes and deployment of plants carrying related R gene would contribute to disease management (Gibriel et al., 2016).

*Sclerotinia sclerotiorum* carried SCFE1 elicitor which evokes MTI responses and is sensed by RLP30, which confirms the association of MTI in resistance to *S. sclerotiorum* (Zhang et al., 2013). Some other elicitors, such as HRE (A heat-released elicitor) (Bertinetti and Ugalde, 1996), SsCut (cutinase) (Zhang et al., 2014), and SsSm1 (a Cerato-platanin family protein) (Pan et al., 2018), have also been identified from *S. sclerotiorum*. On being challenged by Sclerotinia, these elicitors can activate the plant immune system against the invading pathogen (Wang et al., 2019). A large number of chemical elicitors such as salicylic acid, benzothiadiazole (Thakur et al., 2014), Chitosan, *b*-aminobutyric acid, 2, 6-dichloro-isonicotinic acid (Mamgain et al., 2019), methyl jasmonate, glucose (Augustine and Bisht, 2015), arachidonic acid (Neerja and Sohal, 2012) were applied in the management of the Alternaria blight disease. Thus, the molecular studies should focus on the characterization of pathogen-specific genes and effectors which contribute to virulence and susceptibility of the host, further influencing the improvement in our understanding of the R gene-mediated resistance.

## Conclusion

The identification and introgression of resistant gene sources into *B. juncea* against major fungal pathogens is a difficult procedure owing to the close association of R genes with other agronomic traits, variations in host resistance-response against pathogenic races, the polygenic inheritance of resistance, and gene interactions. Also, the identification of R genes is cumbersome because of factors like genetic variations in host subpopulations and pathotypes, the emergence of new pathotypes, genomic tools used, environmental factors, resistance evaluation techniques, scoring criteria, etc. However, continuous efforts are being made to identify R genes or quantitative trait loci for major fungal diseases of Brassica crops. A stable and long-lasting resistance source is a prerequisite for resistance breeding programs. The cloning of previously characterized R genes in Brassica will help in the identification of plant defense mechanisms that support the search of novel R gene sources. The studies on host–pathogen interactions are important to have an insight into the pathogenesis and response of the host against pathogen infection. The genome assembly database played a vital role in identifying novel R genes as the advanced sequencing technologies provide near-complete genome assemblies of diploids or polyploids. This may allow us to identify race-specific or non-specific R genes.

There is a need for universally accepted robust screening and scoring methods for each host-pathogen systems so that true R gene sources could be identified. To increase host resistance for wider pathogen races, gene pyramiding provides durable resistance and sustainable establishment of crop plants in disease-prone areas. Improving knowledge about elicitors and effectors is also important to develop successful disease management strategies. The development of novel pre-breeding material from close or distant resistant wild relatives and marker-assisted selection approach may help to efficiently facilitate Brassica-improvement programs. This review presents a comprehensive analysis of the R gene sources and their utilization in *B. juncea* for improvement through advanced molecular techniques.

## Author Contributions

KS and PK conceptualized and drafted the manuscript, prepared the tables and figures. PK and PR edited and finalized the manuscript. All authors read and approved the final manuscript.

## Conflict of Interest

The authors declare that the research was conducted in the absence of any commercial or financial relationships that could be construed as a potential conflict of interest.

## References

[B1] Adam L. Somerville S. C. (1996). Genetic characterization of five powdery mildew disease resistant loci in *Arabidopsis thaliana*. *Plant J.* 9 341–356. 10.1046/j.1365-313x.1996.09030341.x 8919911

[B2] Agarwal R. A. K. Kumar A. Thakur H. L. (1997). Effect of *Sclerotinia* rot on oil quality in low erucic acid cultivars of rapeseed. *Crucif. Newslett.* 19 103–104.

[B3] Agnihotri A. Gupta K. Prem D. Sarkar G. Mehra V. S. Zargar S. M. (2009). “Genetic enhancement in rapeseed- mustard for quality and disease resistance through in vitro techniques,” in *Proceedings of 16th Australian Research Assembly on Brassicas*, Ballarat.

[B4] Agnihotri A. Lakshmikumaran M. S. Jagannathan V. Shivanna K. R. (1991). Wide hybridization for improvement in cultivated *Brassicas*. *Acta Hortic.* 289 213–214. 10.17660/actahortic.1991.289.49

[B5] Ahmed N. U. Park J. I. Jung H. J. Seo M. S. Kumar T. S. Lee I. H. (2012). Identification and characterization of stress resistance related genes of *Brassica rapa*. *Biotechnol. Lett.* 34 979–987. 10.1007/s10529-012-0860-4 22286206

[B6] Ali S. Mir Z. A. Tyagi A. Mehari H. Meena R. P. Bhat J. A. (2017). Overexpression of NPR1 in *Brassica juncea* confers broad spectrum resistance to fungal pathogens. *Front. Plant Sci*. 8:1693. 10.3389/fpls.2017.01693 29046679 PMC5632730

[B7] Alkooranee J. T. Yin Y. Aledan T. R. Jiang Y. Lu G. Wu J. (2015). Systemic resistance to powdery mildew in *Brassica napus* (AACC) and *Raphanus alboglabra* (RRCC) by *Trichoderma harzianum* TH12. *PLoS One* 10:e0142177. 10.1371/journal.pone.0142177 26540161 PMC4634854

[B8] Almasia N. I. Bazzini A. A. Hopp H. E. Vazquez-Rovere C. (2008). Overexpression of *snakin-1* gene enhances resistance to *Rhizoctonia solani* and *Erwinia carotovora* in transgenic potato plants. *Mol. Plant Pathol.* 9 329–338. 10.1111/j.1364-3703.2008.00469.x 18705874 PMC6640289

[B9] Aneja J. K. Agarwal A. Agnihotri A. (2014). Inter and intra-specific diversity in *Alternaria* species infecting oilseed *Brassicas* in India. *J. Oilseed Brass.* 5 102–117.

[B10] Arora H. Padmaja K. L. Paritosh K. Mukhi N. Tewari A. K. Mukhopadhyay A. (2019). BjuWRR1, a CC-NB-LRR gene identified in *Brassica juncea*, confers resistance to white rust caused by *Albugo candida*. *Theoret. Appl. Genet.* 132 2223–2236. 10.1007/s00122-019-03350-z 31049632

[B11] Attanayake R. N. Tennekoon V. Johnson D. A. Porter L. D. del Río-Mendoza L. Jiang D. (2014). Inferring outcrossing in the homothallic fungus *Sclerotinia sclerotiorum* using linkage disequilibrium decay. *Heredity* 113 353–363. 10.1038/hdy.2014.37 24781807 PMC4181068

[B12] Aubertot J. N. Sohbi Y. Brun H. Penaud A. Nutter F. W. (2006). Phomadidacte: a computer aided training program for the severity assessment of phoma stem canker of oilseed rape. *IOBC Bull*. 29 247–254.

[B13] Augustine R. Bisht N. C. (2015). Biotic elicitors and mechanical damage modulate glucosinolate accumulation by co-ordinated interplay of glucosinolate biosynthesis regulators in polyploid *Brassica juncea*. *Phytochemistry* 117 43–50. 10.1016/j.phytochem.2015.05.015 26057228

[B14] Awasthi R. P. Kolte S. J. (1989). Variability in *Alternaria brassicae* affecting rapeseed and mustard. *Indian Phytopath.* 42:275.

[B15] Axelsson T. Bowman C. M. Sharpe A. G. Lydiate D. J. Lagercrantz U. (2000). Amphidiploid *Brassica juncea* contains conserved progenitor genomes. *Genome* 43 679–688. 10.1139/gen-43-4-679 10984181

[B16] Ayuke F. O. Lagerlöf J. Jorge G. Söderlund S. Muturi J. J. Sarosh B. R. (2017). Effects of biocontrol bacteria and earthworms on the severity of *Alternaria brassicae* disease and the growth of oilseed rape plants (*Brassica napus*). *Appl. Soil Ecol.* 117–118 63–69. 10.1016/j.apsoil.2017.04.019

[B17] Bains P. S. Tewari J. P. (1987). Purification, chemical characterization and host-specificity of the toxin produced by *Alternaria brassicae*. *Physiol. Mol. Plant Pathol*. 30 259–271. 10.1016/0885-5765(87)90039-7

[B18] Bains S. S. Jhooty J. S. (1979). Mixed infections by *Albugo candida* and *Peronospora parasitica* on *Brassica juncea* inflorescence and their control. *Indian Phytopathol.* 32 268–271.

[B19] Banga S. S. Banga S. K. Labana K. S. (1983). “Nucleo-cytoplasmic interactions in *Brassica*,” in *Proceedings of 6th International Rapeseed Congress, 602-606, Paris, France. Groupe Consultatif International de Recherche sur la Colza [International Consultative Group for Research on Rapeseed]*, Paris.

[B20] Barari H. Alavi V. Badalyan S. M. (2012). Genetic and morphological differences among populations of *Sclerotinina sclerotiorum* by microsatellite markers, mycelial compatibility groups (MCGs) and aggressiveness in North Iran. *Roman. Agric. Res.* 29 323–331.

[B21] Barbetti M. J. (1981). Effects of sowing date and oospore seed contamination upon subsequent crop incidence of white rust (*Albugo candida*) in rapeseed. *Aus. J. Plant Pathol.* 10 44–46. 10.1071/app9810044

[B22] Barbetti M. J. Banga S. K. Fu T. D. Li Y. C. Singh D. Liu S. Y. (2014). Comparative genotype reactions to *Sclerotinia sclerotiorum* within breeding populations of *Brassica napus* and *B. juncea* from India and China. *Euphytica* 197 47–59. 10.1007/s10681-013-1049-1

[B23] Barbetti M. J. Carter E. C. (1986). “Diseases of rapeseed,” in *Rapeseed in Western Australia*, ed. Lawson J. A. (South Perth, WA: Western Australian Department of Agriculture), 14–19. Bulletin No. 4105.

[B24] Barret P. Guerif J. Reynoird J. P. Delourme R. Eber F. Renard M. (1998). Selection of stable *Brassica napus-Brassica juncea* recombinant lines resistant to blackleg (*Leptosphaeria maculans*). 2. A ‘to and fro’ strategy to localize and characterize inter-specific introgressions on the *B. napus* genome. *Theor. Appl. Genet.* 96 1097–1103. 10.1007/s001220050844

[B25] Bayer P. E. Hurgobin B. Golicz A. A. Chan C. K. K. Yuan Y. Lee H. T. (2017). Assembly and comparison of two closely related *Brassica napus* genomes. *Plant Biotechnol. J.* 15 1–9. 10.1111/pbi.12742 28403535 PMC5698052

[B26] Bednarek P. (2012). Sulfur-containing secondary metabolites from *Arabidopsis thaliana* and other *Brassicaceae* with function in plant immunity. *Chembiochem* 13 1846–1859. 10.1002/cbic.201200086 22807086

[B27] Berkeley M. J. (1836). “Fungi,” in *The English Flora*, Vol. 5 ed. Smith J. E. (London: BHL), 339.

[B28] Berkeley M. J. (1848). On the white rust of cabbages. *J. Hort. Soc. Lond.* 3 265–271.

[B29] Berkenkamp B. Kirkham C. (1989). Canola disease survey in N.E. Saskatchewan, 1988. *Can. Plant Dis. Sur.* 69:62.

[B30] Bertinetti C. Ugalde R. A. (1996). Studies on the response of carrot cells to a *Sclerotinia sclerotiorum* elicitor: induction of the expression of an extracellular glycoprotein mRNA. *Mol. Plant Microb. Interact.* 9 658–663. 10.1094/MPMI-9-0658 8810081

[B31] Bhayana L. Paritosh K. Arora H. Yadava S. K. Singh P. Nandan D. (2020). A mapped locus on LG A6 of *Brassica juncea* line Tumida conferring resistance to white rust contains a CNL type R gene. *Front. Plant Sci.* 10:1690. 10.3389/fpls.2019.01690 31998351 PMC6960627

[B32] Bittner-Eddy P. D. Crute I. R. Holub E. B. Beynon J. L. (2000). RPP13 is a simple locus in *Arabidopsis thaliana* for alleles that specify downy mildew resistance to different avirulence determinants in *Peronospora parasitica*. *Plant J.* 21 177–188. 10.1046/j.1365-313x.2000.00664.x 10743658

[B33] Bohlmann H. (1994). The role of thionins in plant protection. *Crit. Rev. Plant Sci.* 13 1–16. 10.1080/713608052

[B34] Bohlmann H. Vignutelli A. Hilpert B. Miersch O. Wasternack C. Apel K. (1998). Wounding and chemicals induce expression of the *Arabidopsis thaliana* gene *Thi2.1*, encoding a fungal defense thionin, via the octadecanoid pathway. *FEBS Lett.* 437 281–286. 10.1016/s0014-5793(98)01251-4 9824308

[B35] Boland G. J. Hall R. (1994). Index of plant hosts of *Sclerotinia*. *Can. J. Plant Pathol.* 16 93–108. 10.1080/07060669409500766

[B36] Borhan M. H. Brose E. Beynon J. L. Holub E. B. (2001). White rust (*Albugo candida*) resistance loci on three *Arabidopsis* chromosomes are closely linked to downy mildew (*Peronospora parasitica*) resistance loci. *Mol. Plant Pathol.* 2 87–95. 10.1046/j.1364-3703.2001.00056.x 20572995

[B37] Borhan M. H. Gunn N. Cooper A. Gulden S. Tor M. Rimmer S. R. (2008). WRR4 encodes a TIR-NB-LRR protein that confers broad-spectrum white rust resistance in *Arabidopsis thaliana* to four physiological races of *Albugo candida*. *MPMI* 21 757–768. 10.1094/mpmi-21-6-0757 18624640

[B38] Borhan M. H. Holub E. B. Kindrachuk C. Omidi M. Ozorgmanesh-Frad G. Rimmer S. R. (2010). *WRR4*, a broad-spectrum *TIR-NB-LRR* gene from *Arabidopsis thaliana* that confers white rust resistance in transgenic oilseed *Brassica* crops. *Mol. Plant Pathol.* 11 283–291. 20447277 10.1111/j.1364-3703.2009.00599.x PMC6640464

[B39] Botella M. A. Parker J. E. Frost L. N. Bittner-Eddy O. D. Beynon J. L. Daniels J. B. (1998). Three genes of the *Arabidopsis* RPP1 complex resistance locus recognize distinct *Peronospora parasitica* avirulence determinants. *Plant Cell* 10 1847–1860. 10.2307/3870908 9811793 PMC143951

[B40] Brasier C. M. (2000). Plant pathology: the rise of the hybrid fungi. *Nature* 405 134–135. 10.1038/35012193 10821256

[B41] Brasier C. M. (2001). Rapid evolution of introduced plant pathogens via intespecific hybridization is leading to rapid evolution of Dutch elm disease and other fungal plant pathogens. *Bioscience* 51 123–133. 10.1641/0006-3568(2001)051[0123:reoipp]2.0.co;2

[B42] Bremer H. Lsmen H. Karel G. Ozkan H. Ozkan M. (1947). Contribution to knowledge of the parasitic fungi of Turkey. *Rev. Facul. Sci. Univ. Istanbul Ser. B* 13 122–172.

[B43] Brown J. K. M. Hovmoller M. S. (2002). Aerial dispersal of pathogens on the global and continental scales and its impact on plant disease. *Science* 297 537–541. 10.1126/science.1072678 12142520

[B44] Brown P. D. Morra M. J. (1997). Control of soil-borne plant pests using glucosinolate-containing plants. *Adv. Agron.* 61 167–231. 10.1016/s0065-2113(08)60664-1

[B45] Browne L. M. Conn K. L. Ayer W. A. Tewari J. P. (1991). The Camalexins: new phytoalexins produced in the leaves of *Camelina sativa* (Cruciferae). *Etrahedron* 47 3909–3914. 10.1016/s0040-4020(01)86431-0

[B46] Brun H. Chèvre A. M. Fitt B. D. Powers S. Besnard A. L. Ermel M. (2010). Quantitative resistance increases the durability of qualitative resistance to *Leptosphaeria maculans* in *Brassica napus*. *New Phytol.* 185 285–299. 10.1111/j.1469-8137.2009.03049.x 19814776

[B47] Brun H. Plessis J. Renard M. (1987). “Resistance of some crucifers to *Alternaria brassicae* (Berk.) Sacc,” in *Proceedings of the Seventh International Rapeseed Congress*, Poznan.

[B48] Cai X. Wang X. Liu B. Wu J. Liang J. Cui Y. (2017). *Brassica rapa* genome 2.0: a reference upgrade through sequence re-assembly and gene re-annotation. *Mol. Plant* 10 649–651. 10.1016/j.molp.2016.11.008 27890636

[B49] Cammue B. P. A. De Bolle M. F. C. Terras F. R. G. Proost P. Van Damme J. Rees S. B. (1992). Isolation and characterization of a novel class of plant antimicrobial peptides form *Mirabilis jalapa* L. seeds. *J. Biol. Chem.* 267 2228–2233. 10.1016/s0021-9258(18)45866-8 1733929

[B50] Canola Council of Canada (2020). *Sclerotinia Stem Rot.* Available online at: https://www.canolacouncil.org/canola-encyclopedia/diseases/sclerotinia-stem-rot/ (accessed June 28, 2020).

[B51] Caretto S. Linsalata V. Colella G. Mita G. Lattanzio V. (2015). Carbon fluxes between primary metabolism and phenolic pathway in plant tissues under stress. *Int. J. Mol. Sci.* 16 26378–26394. 10.3390/ijms161125967 26556338 PMC4661826

[B52] Carlier J. D. Alabaça C. A. Coelho P. S. Monteiro A. A. Leitão J. M. (2011). The downy mildew resistance locus Pp523 is located on chromosome C8 of *Brassica oleracea* L. *Plant Breed.* 131 170–175. 10.1111/j.1439-0523.2011.01904.x

[B53] Carlsson M. Bothmer R. V. Merker A. (2004). Screening and evaluation of resistance to downy mildew (*Peronospora parasitica*) and clubroot (*Plasmodiophora brassicae*) in genetic resources of *Brassica* oleracea. *Hereditas* 141 293–300. 10.1111/j.1601-5223.2004.01818.x 15703046

[B54] Cevik V. Boutrot F. Apel W. Robert-Seilaniantz A. Furzer O. J. Redkar A. (2019). Transgressive segregation reveals mechanisms of *Arabidopsis* immunity to *Brassica*-infecting races of white rust (*Albugo candida*). *Proc. Natl. Acad. Sci. U.S.A.* 116 2767–2773. 10.1073/pnas.1812911116 30692254 PMC6377460

[B55] Chalhoub B. Denoeud F. Liu S. Parkin I. A. Tang H. Wang X. (2014). Early allopolyploid evolution in the post-neolithic *Brassica napus* oilseed genome. *Science* 345 950–953. 10.1126/science.1253435 25146293

[B56] Chan Y. L. Prasad V. Sanjaya L. Chen K. H. Liu P. C. Chan M. T. (2005). Transgenic tomato plants expressing an *Arabidopsis thionin* (*Thi2.1*) driven by fruit-inactive promoter battle against phytopathogenic attack. *Planta* 221 386–393. 10.1007/s00425-004-1459-3 15657715

[B57] Chattopadhyay C. Agarwal R. Kumar A. Meena R. L. Faujdar K. Chakravarthy N. V. K. (2011). Epidemiology and development of forecasting models for White rust of *Brassica juncea* in India. *Archiv. Phytopathol. Plant Protect.* 44 751–763. 10.1080/03235400903458571

[B58] Chen X. Hou X. Zhang J. Zheng J. (2008). Molecular characterization of two important antifungal proteins isolated by downy mildew infection in non-heading Chinese cabbage. *Mol. Biol. Rep.* 35 621–629. 10.1007/s11033-007-9132-0 17762976

[B59] Cheung W. Y. Gugel R. K. Landry B. S. (1998). Identification of RFLP markers linked to the white rust resistance gene (*Acr*) in mustard (*Brassica juncea* (L.) Czern. and Coss.). *Genome* 41 626–628. 10.1139/gen-41-4-626

[B60] Chevre A. M. Barret P. Eber F. Dupuy P. Brun H. Tanguy X. (1997). Selection of stable *Brassica napus-B. juncea* recombinant lines resistant to blackleg (*Leptosphaeria maculans*). 1. Identification of molecular markers, chromosomal and genomic origin of the introgression. *Theor. Appl. Genet.* 95 1104–1111. 10.1007/s001220050669

[B61] Chèvre A. M. Brun H. Eber F. Letanneur J. C. Vallee P. Ermel M. (2008). Stabilization of resistance to *Leptosphaeria maculans* in *Brassica napus*-*B. juncea* recombinant lines and its introgression into spring-type *Brassica napus*. *Plant Dis.* 92 1208–1214. 10.1094/pdis-92-8-1208 30769494

[B62] Chevre A. M. Eber F. Margale E. Kerlan M. C. (1994). Comparison of somatic and sexual *Brassica napus Sinapis alba* hybrids and their progeny by cytogenetic studies and molecular characterization. *Genome* 37 367–371. 10.1139/g94-052 18470081

[B63] Chevre A. M. Eber F. This P. Barret P. Tanguy X. Brun H. (1996). Characterization of *Brassica nigra* chromosomes and of blackleg resistance in *B. napus*-*B. nigra* addition lines. *Plant Breed.* 115 113–118. 10.1111/j.1439-0523.1996.tb00884.x

[B64] Chèvre A. M. Leon A. P. Jenczewski E. Eber F. Delourme R. (2003). “Introduction of blackleg resistance from *Brassica rapa* into *Brassica napus*,” in *Proceedings of the 6th Blackleg Workshop*, Copenhagen.

[B65] Chhikara S. Chaudhury D. Dhankher O. P. Jaiwal P. K. (2012). Combined expression of a barley class II chitinase and type I ribosome inactivating protein in transgenic *Brassica juncea* provides protection against *Alternaria brassicae*. *Plant Cell Tiss. Organ Cult.* 108 83–89. 10.1007/s11240-011-0015-7

[B66] Choi Y. J. Hong S. B. Shin H. D. (2006). Genetic diversity within the *Albugo candida* complex (Peronosporales, Oomycota) inferred from phylogenetic analysis of ITS rDNA and COX2 mtDNA sequences. *Mol. Phylogenet. Evol.* 40 400–409. 10.1016/j.ympev.2006.03.023 16644244

[B67] Choi Y. J. Park M. J. Park J. H. Shin H. D. (2011). White blister rust caused by *Albugo candida* on oilseed rape in Korea. *Plant Pathol. J.* 27 192. 10.5423/ppj.2011.27.2.192

[B68] Choi Y. J. Shin H. D. Hong S. B. Thines M. (2007). Morphological and molecular discrimination among *Albugo candida* materials infecting *Capsella bursa-pastoris* world-wide. *Fungal Divers.* 27 11–34.

[B69] Choi Y. J. Shin H. D. Hong S. B. Thines M. (2009). The host range of *Albugo candida* extends from *Brassicaceae* through cleomaceae to capparaceae. *Mycol. Prog.* 8 329–335. 10.1007/s11557-009-0604-6

[B70] Choi Y. J. Shin H. D. Ploch S. Thines M. (2008). Evidence for uncharted biodiversity in the *Albugo candida* complex, with the description of a new species. *Mycol. Res.* 112 1327–1334. 10.1016/j.mycres.2008.04.015 18951775

[B71] Chowdhary S. (1944). Some fungi from Assam. *Indian J. Agric. Sci.* 14 230–233.

[B72] Christianson J. A. Rimmer S. R. Good A. G. Lydiate D. J. (2006). Mapping genes for resistance to *Leptosphaeria maculans* in *Brassica juncea*. *Genome* 49 30–41. 10.1139/G05-085 16462899

[B73] Clarkson J. P. Coventry E. Kitchen J. Carter H. E. Whipps J. M. (2012). Population structure of *Sclerotinia sclerotiorum* in crop and wild hosts in the UK. *Plant Pathol.* 62 309–324. 10.1111/j.1365-3059.2012.02635.x

[B74] Coelho P. S. Leckie D. Bahcevandziev K. Valério L. Astley D. Louise N. (1998). The relationship between cotyledon and adult plant resistance to downy mildew (*Peronospora parasitica*) in *Brassica oleracea*. *Acta Hortic.* 459 335–342. 10.17660/actahortic.1998.459.39

[B75] Coelho P. S. Monteiro A. A. (2018). Genetic and histological characterization of downy mildew resistance at the cotyledon stage in *Raphanus sativus* L. *Euphytica* 214:208. 10.1007/s10681-018-2289-x

[B76] Coelho P. S. Valério L. Monteiro A. A. (2009). Leaf position, leaf age and plant age affect the expression of downy mildew resistance in *Brassica oleracea*. *Eur. J. Plant Pathol.* 125 179–188. 10.1007/s10658-009-9469-4

[B77] Conn K. L. Tewari J. P. (1986). Hypersensitive reaction induced by *Alternaria brassicae* in *Eruca sativa*, an oil-yielding crucifer. *Can. J. Plant Pathol.* 8:348.

[B78] Conn K. L. Tewari J. P. (1990). Survey of *Alternaria* blackspot and *Sclerotinia* stem rot in central Alberta in 1989. *Can. Plant Dis. Surv.* 70 66–67.

[B79] Conn K. L. Tewari J. P. Awasthi R. P. (1990). A disease assessment key for *Alternaria* black spot in rapeseed and mustard. *Can. Plant Dis. Surv.* 70:1.

[B80] Conn K. L. Tewari J. P. Dahiya J. S. (1988). Resistance to *Alternaria brassicae* and phytoalexins-elicitation in rapeseed and other crucifers. *Plant Sci.* 56 21–25. 10.1016/0168-9452(88)90180-X

[B81] Cooper A. J. Latunde-Dada A. O. Woods-Tor A. Lynn J. Lucas J. A. Crute I. R. (2008). Basic compatibility of *Albugo candida* in *Arabidopsis thaliana* and *Brassica juncea* causes broad-spectrum suppression of innate immunity. *Mol. Plant Microb. Interact.* 21 745–756. 10.1094/MPMI-21-6-0745 18624639

[B82] Dang J. K. Sangwan M. S. Mehta N. Kaushik C. D. (2000). Multiple disease resistance against four fungal foliar diseases of rapeseed-mustard. *Indian Phytopath.* 53 455–458.

[B83] Dange K. K. Patel R. L. Patel S. I. Patel K. K. (2002). Assessment of losses in yield due to powdery mildew disease in mustard under north Gujarat conditions. *J. Mycol. Plant Pathol.* 32 249–250.

[B84] Delourme R. Pilet-Nayel M. L. Archipiano M. Horvais R. Tanguy X. Rouxel T. (2004). A cluster of major specific resistance genes to *Leptosphaeria maculans* in *Brassica napus*. *Phytopathology* 94 578–583. 10.1094/phyto.2004.94.6.578 18943482

[B85] Delwiche P. A. Williams P. H. (1974). Resistance to *Albugo candida* race 2 in *Brassica* sp. *Proc. Amer. Phytopathol. Soc.* 1:66.

[B86] Desai A. G. Chattopadhyay C. Agrawal R. Kumar A. Meena R. L. Meena P. D. (2004). *Brassica juncea* powdery mildew epidemiology and weatherbased forecasting models for India - a case study. *J. Plant Dis. Protect.* 111 429–438.

[B87] Dickson M. H. Petzoldt R. (1993). Plant age and isolate source affect expression of downy mildew resistance in broccoli. *Hortscience* 28 730–731. 10.21273/hortsci.28.7.730

[B88] Dilmaghani A. Balesdent M. Didier J. Wu C. Davey J. Barbetti M. J. (2009). The *Leptosphaeria maculans-Leptosphaeria biglobosa* species complex in the American continent. *Plant Pathol.* 58 1044–1058.

[B89] Dixelius C. (1999). Inheritance of the resistance to *Leptosphaeria maculans* of *Brassica nigra* and *B. juncea* in near isogenic lines of *B. napus*. *Plant Breed.* 118 151–156. 10.1046/j.1439-0523.1999.118002151.x

[B90] Dixelius C. Wahlberg S. (1999). Resistance to *Leptosphaeria maculans* is conserved in a specific region of the *Brassica* B genome. *Theor. Appl. Genet.* 99 368–372. 10.1007/s001220051246

[B91] Doughty K. J. Blight M. M. Bock C. H. Fieldsend J. K. Pickett J. A. (1996). Release of alkenyl isothiocyanates and other volatiles from *Brassica rapa* seedlings during infection by *Alternaria brassicae*. *Phytochemistry* 43 371–374. 10.1016/0031-9422(96)00189-6

[B92] Ebrahimi A. G. Delwiche P. A. Williams P. H. (1976). Resistance in *Brassica juncea* to *Peranospora parasítica* an*d Albugo candida* Race 2. *Proc. Am. Phytopathol. Soc.* 3:273.

[B93] Elliott V. L. Norton R. M. Khangura R. K. Salisbury P. A. Marcroft S. J. (2015). Incidence and severity of blackleg caused by *Leptosphaeria* spp. in juncea canola (*Brassica juncea* L.) in Australia. *Austral. Plant Pathol.* 44 149–159. 10.1007/s13313-014-0337-0

[B94] Epple P. Apel K. Bohlmann H. (1997). Overexpression of an endogenous thionin enhances resistance of *Arabidopsis* against *Fusarium oxysporum*. *Plant Cell* 9 509–520. 10.2307/3870503 9144959 PMC156935

[B95] Farinhó M. Coelho P. Monteiro A. Leitão J. (2007). SCAR and CAPS markers flanking the *Brassica oleracea* L. Pp523 downy mildew resistance locus demarcate a genomic region syntenic to the top arm end of *Arabidopsis thaliana* L. chromosome 1. *Euphytica* 157 215–221. 10.1007/s10681-007-9414-6

[B96] Farnham M. W. Wang M. Thomas C. E. (2002). A single dominant gene for downy mildew resistance in broccoli. *Euphytica* 128 405–407.

[B97] Farr D. F. Bills G. F. Chamuris G. P. Rossman A. Y. (1989). “Ipomoea,” in *Fungi on Plants and Plant Products in the United States*, eds Farr D. F. Billis F. G. Chamuris G. P. Rossman A. Y. (St Paul: APS Press), 142–143.

[B98] Farr D. F. Rossman A. Y. (2013). *Fungal Databases,” Systematic Mycology and Microbiology Laboratory, ARS, USDA.* Available online at: http://nt.Ars-grin.gov/fungaldatabases/ (accessed June 28, 2020).

[B99] Fatima U. Bhorali P. Borah S. Kumar M. S. (2019). Perspectives on the utilization of resistance mechanisms from host and nonhost plants for durable protection of *Brassica* crops against *Alternaria* blight. *PeerJ* 7:e7486. 10.7717/peerj.7486 31579565 PMC6766370

[B100] Ferreira M. E. Williams P. H. Osborn T. C. (1994). RFLP mapping of *Brassica napus* using doubled haploid lines. *Theor. Appl. Genet.* 89 615–621. 10.1007/bf00222456 24177938

[B101] Fradin E. F. Abd-El-Haliem A. Masini L. Van den Berg G. C. M. Joosten M. H. A. J. Thomma B. P. H. J. (2011). Interfamily Transfer of Tomato Ve1 Mediates Verticillium Resistance in *Arabidopsis*. *Plant Physiol.* 156 2255–2265. 10.1104/pp.111.180067 21617027 PMC3149960

[B102] Franks S. J. (2011). Plasticity and evolution in drought avoidance and escape in the annual plant *Brassica rapa*. *New Phytol.* 190 249–257. 10.1111/j.1469-8137.2010.03603.x 21210818

[B103] Fredua-Agyeman R. Coriton O. Huteau V. Parkin I. A. P. Chevre A. M. Rahman H. (2014). Molecular cytogenetic identification of B genome chromosomes linked to blackleg disease resistance in *Brassica napus* × *B. carinata* interspecific hybrids. *Theor. Appl. Genet.* 127 1305–1318. 10.1007/s00122-014-2298-7 24687759

[B104] Freeman B. C. Beattie G. A. (2008). An overview of plant defenses against pathogens and herbivores. *Plant Health Instr.* 10.1094/phi-i-2008-0226-01

[B105] Frye C. A. Innes R. W. (1998). An *Arabidopsis* mutant with enhanced resistance to powdery mildew. *Plant Cell* 10 947–956. 10.2307/3870681 9634583 PMC144036

[B106] Gaebelein R. Alnajar D. Koopmann B. Mason A. (2019). Hybrids between *Brassica napus* and *B. nigra* show frequent pairing between the B and A/C genomes and resistance to blackleg. *Chrom. Res.* 27 221–236. 10.1007/s10577-019-09612-2 31280459

[B107] Gaikwad K. Kirti P. B. Sharma A. Prakash S. Chopra V. L. (1996). Cytogenetical and molecular investigations on somatic hybrids of *Sinapis alba* and *Brassica juncea* and their backcross progeny. *Plant Breed.* 115 480–483. 10.1111/j.1439-0523.1996.Tb00961.x

[B108] Garg H. Atri C. Sandhu P. S. Kaur B. Renton M. Banga S. K. (2010). High level of resistance to *Sclerotinia sclerotiorum* in introgression lines derived from hybridization between wild crucifers and the crop *Brassica* species *B. napus and B. juncea*. *Field Crops Res.* 117 51–58. 10.1016/j.fcr.2010.01.013

[B109] Garg H. Sivasithamparam K. Banga S. S. Barbetti M. J. (2008). Cotyledon assay as a rapid and reliable method of screening for resistance against *Sclerotinia sclerotiorum* in *Brassica napus* genotypes. *Austral. Plant Pathol.* 37 106–111. 10.1071/ap08002

[B110] Ge X. T. You M. P. Barbetti M. J. (2015). Virulence differences among *Sclerotinia sclerotiorum* isolates determines host cotyledon resistance responses in *Brassicaceae* genotypes. *Eur. J. Plant Pathol.* 143 527–541. 10.1007/s10658-015-0696-6

[B111] Ghasolia R. P. Shivpuri A. Bhargava A. K. (2004). *Sclerotinia* rot of Indian mustard (*Brassica juncea*) in Rajasthan. *Indian Phytopathol.* 57 76–79.

[B112] Gibriel H. A. Thomma B. P. Seidl M. F. (2016). The age of effectors: genome-based discovery and applications. *Phytopathology* 106 1206–1212. 10.1094/PHYTO-02-16-0110-FI 27050568

[B113] Gladders P. (1987). Current status of diseases and disease control in winter oilseed rape in England and Wales. *Bull. SROP* 10 7–10.

[B114] Golicz A. A. Bayer P. E. Barker G. C. Edger P. P. Kim H. Martinez P. A. (2016). The pangenome of an agronomically important crop plant *Brassica oleracea*. *Nat. Commun.* 7:13390. 10.1038/ncomms13390 27834372 PMC5114598

[B115] Goyal P. Chattopadhyay C. Mathur A. P. Kumar A. Meena P. D. Datta S. (2013). Pathogenic and molecular variability among *Brassica* isolates of *Alternaria brassicae* from India. *Ann. Plant Protect. Sci.* 21 349–359.

[B116] Gupta M. L. Singh G. Raheja R. K. Ahuja K. L. Banga S. K. (1997). Chlorophyll content in relation to white rust (*A. candida*) resistance in Indian mustard. *Crucif. Newslett.* 19 105–106.

[B117] Gupta N. C. Sharma P. Rao M. Rai P. K. Gupta A. K. (2020). Evaluation of non-injury inoculation technique for assessing *Sclerotinia* stem rot (*Sclerotinia sclerotiorum*) in oilseed *Brassica*. *J. Microbiol. Methods* 175:105983. 10.1016/j.mimet.2020.105983 32544486

[B118] Gyawali S. Harrington M. Durkin J. Hornerauthor K. (2016). Microsatellite markers used for genome-wide association mapping of partial resistance to *Sclerotinia sclerotiorum* in a world collection of *Brassica napus*. *Mol. Breed.* 36:72. 10.1007/s11032-016-0496-5 27330402 PMC4889634

[B119] Hammett K. P. W. (1969). White rust diseases. *New Zeal. Gardner* 26:43.

[B120] Hansen L. H. Earle E. D. (1995). Transfer of resistance to *Xanthomonas cam-pestris* pv. *campestris*(L.) by proto-plast fusion. *Theor. Appl. Genet.* 91 1293–1300. 10.1007/BF00220944 24170061

[B121] Hansen L. H. Earle E. D. (1997). Somatic hybrids between *Brassica oleracea* (L.) and *Sinapis alba* (L.) with re-sistance to *Alternaria brassicae* (Berk.) Sacc. *Theor. Appl. Genet.* 94 1078–1085. 10.1007/s001220050518

[B122] Hao G. Stover E. Gupta G. (2016). Overexpression of a modified plant thionin enhances disease resistance to citrus canker and huanglongbing (HLB). *Front. Plant Sci.* 7:1078. 10.3389/fpls.2016.01078 27499757 PMC4956653

[B123] Hao J. J. Subbarao K. V. Duniway J. M. (2003). Germination of *Sclerotinia minor* and *S. sclerotiorum sclerotia* under various soil moisture and temperature combinations. *Phytopathology* 93 443–450. 10.1094/phyto.2003.93.4.443 18944359

[B124] Harper F. R. Pittman U. J. (1974). Yield loss by *Brassica campestris* and *Brassica napus* from systemic stem infection by *Albugo curciferarum*. *Phytopathology* 64 408–410. 10.1094/phyto-64-408

[B125] Hemmati R. Javan-Nikkhah M. Linde C. C. (2009). Population genetic structure of *Sclerotinia sclerotiorum* on canola in Iran. *Eur. J. Plant Pathol.* 125 617–628. 10.1007/s10658-009-9510-7

[B126] Hirata S. (1954). Studies on the phytohormone in the malformed portion of the diseased plants. I. The relation between the growth rate and the amount of free auxin in the fungous galls and virusinfected plants. *Ann. Phytopathol. Soc. Jpn.* 19 33–38. 10.3186/jjphytopath.19.33

[B127] Holtorf S. Ludwig-Müller J. Apel K. Bohlmann H. (1998). High-level expression of a viscotoxin in *Arabidopsis thaliana* gives enhanced resistance against *Plasmodiophora brassicae*. *Plant Mol. Biol.* 36 673–680. 9526499 10.1023/a:1005947904830

[B128] Hong C. X. Fitt B. D. L. (1995). Effects of inoculum concentration, leaf age and wetness period on the development of dark leaf and pod spot (*Alternaria brassicae*) on oilseed rape (*Brassica napus*). *Plant Dis.* 127 283–295. 10.1111/j.1744-7348.1995.Tb06673.x

[B129] Hoshikawa K. Ishihara G. Takahashi H. Nakamura I. (2012). Enhanced resistance to gray mold (*Botrytis cinerea*) in transgenic potato plants expressing thionin genes isolated from *Brassicaceae* species. *Plant Biotechnol.* 29 87–93. 10.5511/plantbiotechnology.12.0125a

[B130] Hossain M. R. Ferdous M. J. Park J. Robin A. H. K. Natarajan S. Jung H. (2020). In-silico identification and differential expression of putative disease resistance-related genes within the collinear region of *Brassica napus* blackleg resistance locus *LepR2’* in *Brassica oleracea*. *Hortic. Environ. Biotechnol.* 61 879–890. 10.1007/s13580-020-00271-5

[B131] Howlett B. J. Idnurm A. Pedras M. S. C. (2001). *Leptosphaeria maculans*, the causal agent of blackleg disease of *Brassicas*. *Fungal Genet. Biol.* 33 1–14. 10.1006/fgbi.2001.1274 11407881

[B132] Huang Y. J. Jestin C. Welham S. J. King G. J. Manzanares-Dauleux M. J. Fitt B. D. L. (2016). Identification of environmentally stable QTL for resistance against *Leptosphaeria maculans* in oilseed rape (*Brassica napus*). *Theor. Appl. Genet.* 129 169–180. 10.1007/s00122-015-2620-z 26518572 PMC4703627

[B133] Huang Y. J. Paillard S. Kumar V. King G. J. Fitt B. D. L. Delourme R. (2019). Oilseed rape (*Brassica napus*) resistance to growth of *Leptosphaeria maculans* in leaves of young plants contributes to quantitative resistance in stems of adult plants. *PLoS One* 14:e0222540. 10.1371/journal.pone.0222540 31513677 PMC6742359

[B134] Hulbert S. H. Webb C. A. Smith S. M. Sun Q. (2001). Resistance gene complexes: evolution and utilization. *Ann. Rev. Phytopathol.* 39 285–312. 10.1146/annurev.phyto.39.1.285 11701867

[B135] Humaydan H. S. Williams P. H. (1976). Inheritance of seven characters in *Raphanus sativus*. *Hort. Sci.* 11 146–147.

[B136] Inturrisi F. C. (2018). *Genome-Wide Analysis of NBS-LRR genes in Indian Mustard (Brassica juncea) and Prediction of Candidate Disease Resistance Genes.* Ph. D. thesis, The University of Western Australia, Perth. 10.26182/5b57f1254d265

[B137] Jejelowo O. A. Conn K. L. Tewari J. P. (1991). Relationship between conidial concentration, germling growth and phytoalexin production by *Camelina sativa* leaves inoculated with *Alternaria brassicae*. *Mycol. Res.* 95 928–934. 10.1016/s0953-7562(09)80089-0

[B138] Jensen B. D. Værbak S. Munk L. Andersen S. B. (1999). Characterization and inheritance of partial resistance to downy mildew, *Peronospora parasitica*, in breeding material of broccoli, *Brassica oleracea* convar. botrytis var. italica. *Plant Breed.* 118 549–554. 10.1046/j.1439-0523.1999.00409.x

[B139] Jimenez L. D. Ayer W. A. Tewari J. P. (1997). Phytoalexins produced in leaves of *Capsella bursa-pastoris* (Shepherd’s Purse). *Phytoprotection* 78 99–103. 10.7202/706124ar 33270396

[B140] Jones D. Gray E. G. (1973). Factors affecting germination of sclerotia of *Sclerotinia sclerotiorum* from peas. *Trans. Br. Mycol. Soc.* 60 495–500. 10.1016/s0007-1536(73)80033-6

[B141] Jones F. M. H. Phelps K. (1989). Climatic factors influencing spore production in *Alternaria brassicae* and *Alternaria brassicicola*. *Plant Pathol.* 114 449–458. 10.1111/j.1744-7348.1989.Tb03360.x

[B142] Jouet A. Saunders D. G. O. McMullan M. Ward B. Furzer O. Jupe F. (2019). *Albugo candida* race diversity, ploidy and host-associated microbes revealed using DNA sequence capture on diseasedplants in the field. *New Phytol.* 221 1529–1543. 10.1111/nph.15417 30288750

[B143] Jourdan P. Salazar E. (1993). *Brassica carinata* resynthesized by protoplast fusion. *Theor. Appl. Genet.* 86 567–572. 10.1007/BF00838710 24193704

[B144] Kaczmarek J. Jêdryczka M. (2011). Characterization of two coexisting pathogen populations of *Leptosphaeria* spp., the cause of stem canker of *Brassicas*. *Acta Agrobot.* 64 3–14. 10.5586/aa.2011.012

[B145] Kadian A. K. Saharan G. S. (1983). Symptomatology, host range and assessment oflosses due to *Alternaria brassica*e infection inrapeseed and mustard. *Indian J. Mycol. Pl. Pathol.* 13 319–323.

[B146] Kamble A. Bhargava S. (2007). β-Aminobutyric acid-induced resistance in *Brassica juncea* against the necrotrophic pathogen *Alternaria brassicae*. *J. Phytopathol.* 155 152–158. 10.1111/j.1439-0434.2007.01209.x

[B147] Kanrar S. Venkateswari J. C. Kirti P. B. Chopra V. L. (2002). Transgenic expression of hevein, the rubber tree lectin, in Indian mustard confers protection against *Alternaria brassicae*. *Plant Sci.* 162 441–448. 10.1016/s0168-9452(01)00588-x

[B148] Kanzaki H. Nirasawa S. Saitoh H. Ito M. Nishihara M. Terauchi R. (2002). Overexpression of the wasabi defensin gene confers enhanced resistance to blast fungus (*Magnaporthe grisea*) in transgenic rice. *Theor. Appl. Genet.* 105 809–814. 10.1007/s00122-001-0817-9 12582903

[B149] Kaur P. Banga S. Kumar N. Gupta S. Akhatar J. Banga S. S. (2014). Polyphyletic origin of *Brassica juncea* with *B. rapa* and *B. nigra* (*Brassicaceae*) participating as cytoplasm donor parents in independent hybridization events. *Am. J. Bot.* 101 1157–1166. 10.3732/ajb.1400232 25030348

[B150] Kaur P. Jost R. Sivasithamparam K. Barbetti M. J. (2011a). Proteome analysis of the *Albugo candida*-*Brassica juncea* pathosystem reveals that the timing of the expression of defense-related genes is a crucial determinant of pathogenesis. *J. Exper. Bot.* 62 1285–1298. 10.1093/jxb/erq365 21193577 PMC3022411

[B151] Kaur P. Sivasithamparam K. Li H. Barbetti M. J. (2011b). Pre-inoculation with *Hyaloperonospora parasitica* reduces incubation period and increases severity of disease caused by *Albugo candida* in a *Brassica juncea* variety resistant to downy mildew. *J. Gen. Plant Pathol.* 77 101–106. 10.1007/s10327-011-0293-2

[B152] Kaur P. Li C. X. Barbetti M. J. You M. P. Li H. Sivasithamparam K. (2008). First report of powdery mildew caused by *Erysiphe cruciferarum* on *Brassica juncea* in Australia. *Plant Dis.* 92:650. 10.1094/pdis-92-4-0650c 30769625

[B153] Kaur S. Singh G. Banga S. S. (2007). “Documenting variation in *Alternaria brassicae* isolates based on conidial morphology, fungicidal sensitivity and molecular profile,” in *Proceedings of the International Rapeseed Congress-iv on Sustainable Development in Cruciferous Oilseed Crops Production*, Wuhan.

[B154] Kevin J. D. Blight M. M. Bock C. H. Fieldsend J. K. Picket J. A. (1996). Release of alkenyl isothiocyanates and other volatiles from *brassica rapa* seedlings during infection by *Alternaria brassicae*. *Phytochemistry* 43 371–374.

[B155] Khan R. S. Nishihara M. Yamamura S. Nakamura I. Mii M. (2006). Transgenic potatoes expressing wasabi defensin peptide confer partial resistance to gray mold (*Botrytis cinerea*). *Plant Biotechnol.* 23 179–183. 10.5511/plantbiotechnology.23.179

[B156] Khangura R. Barbetti M. J. (2001). Prevalence of blackleg (*Leptosphaeria maculans*) on canola (*Brassica napus*) in Western Australia. *Austr. J. Exper. Agric.* 41 71–80. 10.1071/ea00068

[B157] Kiermayer O. (1958). Paper chromatographic studies of the growth substances of *Capsella bursa-pastoris* after infection by *Albugo candida* and *Peronospora parasitica*. *Osterr. Bot. Z.* 105 515–528.

[B158] Kim J. Y. Kim B. S. Cho S. E. Shin H. D. (2013). First report of powdery mildew caused by *Erysiphe cruciferarum* on Indian mustard (*Brassica juncea*) in Korea. *Plant Dis.* 97:1383. 10.1094/PDIS-04-13-0378-PDN 30722157

[B159] Kim S. Song Y. H. Lee J.-Y. Choi S. R. Dhandapani V. Jang C. S. (2011). Identification of the BrRHP1 locus that confers resistance to downy mildew in Chinese cabbage (*Brassica rapa* ssp. pekinensis) and development of linked molecular markers. *Theor. Appl. Genet.* 123 1183–1192. 10.1007/s00122-011-1658-9 21814857

[B160] Kirti P. B. Prakash S. Chopra V. L. (1991). Interspecific hybridization between *Brassica juncea* and *B. spinescens* through protoplast fusion. *Plant Cell Rep.* 9 639–642. 10.1007/BF00231806 24213667

[B161] Klemm M. (1938). The most important diseases and pests of Colza and Rape. *Dtsch. Landw.* 20 251–252.

[B162] Kohn L. M. Stasovski E. Carbone I. Royer J. Anderson J. B. (1991). Mycelial incompatibility and molecular markers identify genetic variability in field populations of *Sclerotinia sclerotiorum*. *Phytopathology* 81 480–485. 10.1094/Phyto-81-480

[B163] Koike S. T. Sullivan M. J. Southwick C. Feng C. Correll J. C. (2011). Characterization of White Rust of Perennial Pepperweed Caused by *Albugo candida* in California. *Plant Dis.* 95 876. 10.1094/PDIS-12-10-0912 30731711

[B164] Kole C. Teutonico R. Mengistu A. Williams P. H. Osborn T. C. (1996). Molecular mapping of a locus controlling resistance to *Albugo candida* in *Brassica rapa*. *Phytopathology* 86 367–369. 10.1094/phyto-86-367

[B165] Kolte S. J. (1985). “Diseases of annual edible oilseed crops,” in *Rapeseed-Mustard and Sesame Diseases*, Vol. 2 (Boca Raton, FL: CRC Press Inc), 135.

[B166] Kolte S. J. Bardoloi D. K. Awasthi R. P. (1991). “Thesearch for resistance to major diseases of rapeseed-mustard in India,” in *Proceedings of GCIRC 8th Internationa lRapeseed Congress*, Saskatoon.

[B167] Kumar A. Katoch A. Sharma P. N. Kumari V. Kumar A. (2014). Pathogenic and genetic variability in *Alternaria brassicae* infecting rapeseed-mustard and evaluation of resistance sources. *Indian Phytopathol.* 67 257–262.

[B168] Kumar D. Shekhar S. Bisht S. Kumar V. Varma A. (2015). Ectopic overexpression of lectin in transgenic *Brassica juncea* plants exhibit resistance to fungal phytopathogen and showed alleviation to salt and drought stress. *J. Bioeng. Biomed. Sci.* 5:147. 10.4172/2155-9538.1000147

[B169] Kumar S. (2017). Plant secondary metabolites (PSMs) of *Brassicaceae* and their role in plant defense against insect herbivores -A review. *J. Appl. Nat. Sci.* 9 508–519. 10.31018/jans.v9i1.1222

[B170] Kumar S. Sangwan M. S. Mehta N. Kumar R. (2003). Pathogenic diversity in isolates of *Alternaria brassicae* infecting rapeseed and mustard. *J. Mycol. Pl. Pathol*. 33 59–64.

[B171] Kumari P. Bhat S. R. (2019). Allohexaploid H2 (IC0626000; INGR18031), an Indian mustard (*Brassica juncea*) germplasm with heat tolerance, resistant to *Alternaria brassicae*. *Indian J. Plant Genet. Resourc.* 32:439.

[B172] Kumari P. Bisht D. S. Bhat S. R. (2018). Stable, fertile somatic hybrids between *Sinapis alba* and *Brassica juncea* show resistance to *Alternaria brassicae* and heat stress. *Plant Cell Tiss. Organ Cult.* 133 77–86. 10.1007/s11240-017-1362-9

[B173] Kumari P. Singh K. P. (2019). Characterization of stable somatic hybrids of *Sinapis alba* and *Brassica juncea* for *Alternaria* blight, *Sclerotinia sclerotiorum* resistance and heat tolerance. *Indian Res. J. Ext. Educ*. 19 99–103.

[B174] Kumari P. Singh K. P. Bisht D. Kumar S. (2020b). Somatic hybrids of *Sinapis alba* + *Brassica juncea*: study of backcross progenies for morphological variations, chromosome constitution and reaction to *Alternaria brassicae*. *Euphytica* 216:93. 10.1007/s10681-020-02629-3

[B175] Kumari P. Singh K. P. Kumar S. Yadava D. K. (2020c). Development of a yellow-seeded stable allohexaploid *brassica* through inter-generic somatic hybridization with a high degree of fertility and resistance to *Sclerotinia sclerotiorum*. *Front. Plant Sci.* 11:575591. 10.3389/fpls.2020.575591 PMC7732669 33329636

[B176] Kumari P. Singh K. P. Rai P. K. (2020a). Draft genome of multiple resistance donor plant *Sinapisalba*: an insight into SSRs, annotations and phylogenetics. *PLoS One* 15:e0231002. 10.1371/journal.pone.0231002 PMC7145005 32271806

[B177] Lakra B. S. Saharan G. S. (1989). Correlation of leaf and staghead infection intensities of white rust with yield and yield components of mustard. *Indian J. Mycol. Plant Pathol.* 19 279–281.

[B178] Larkan N. J. Lydiate D. J. Parkin I. A. P. Nelson M. N. Epp D. J. Cowling W. A. (2013). The *Brassica* napus blackleg resistance gene LepR3 encodes a receptor-like protein triggered by the *Leptosphaeria maculans* effector AVRLM1. *New Phytol.* 197 595–605. 10.1111/nph.12043 23206118

[B179] Larkan N. J. Lydiate D. J. Yu F. Rimmer S. R. Borhan M. H. (2014). Co-localisation of the blackleg resistance genes *Rlm2* and *LepR3* on *Brassica napus* chromosome A10. *BMC Plant Biol.* 14:387. 10.1186/s12870-014-0387-z 25551287 PMC4302512

[B180] Lee H. Chawla H. S. Obermeier C. Dreyer F. Abbadi A. Snowdon R. (2020). Chromosome-scale assembly of winter oilseed rape *Brassica napus*. *Front. Plant Sci.* 11:496. 10.3389/fpls.2020.00496 32411167 PMC7202327

[B181] Li C. X. Liu S. Y. Sivasithamparam K. Barbetti M. J. (2008). New sources of resistance to *sclerotinia* stem rot caused by *Sclerotinia sclerotiorum* in Chinese and Australian *Brassica napus* and *Brassica juncea* germplasm screened under Western Australian conditions. *Austr. Plant Pathol.* 38 149–152.

[B182] Li J. Zhao Z. Hayward A. Cheng H. Fu D. (2015). Integration analysis of quantitative trait loci for resistance to *Sclerotinia sclerotiorum* in *Brassica napus*. *Euphytica* 205 483–489. 10.1007/s10681-015-1417-0

[B183] Li Q. Li J. Sun J. L. Ma X. F. Li Y. Wang M. W. (2016). Multiple evolutionary events involved in maintaining homologs of resistance to powdery mildew 8 in *Brassica napus*. *Front. Plant Sci.* 7:1065. 10.3389/fpls.2016.01065 27493652 PMC4955382

[B184] Liban S. H. Cross D. J. Kutcher H. R. Peng G. Fernando W. G. D. (2016). Race structure and frequency of avirulence genes in the western Canadian *Leptosphaeria maculans* pathogen population, the causal agent of blackleg in *Brassica* species. *Plant Pathol.* 65 1161–1169. 10.1111/ppa.12489

[B185] Litholdo J. C. G. Gomes E. V. Lobo M. Nasser L. C. B. Petrofeza S. (2011). Genetic diversity and mycelial compatibility groups of the plant-pathogenic fungus *Sclerotinia sclerotiorum* in Brazil. *Genet. Mol. Res.* 10 868–877. 10.4238/vol10-2gmr937 21644203

[B186] Liu S. Y. Liu R. H. Latunde-Dada A. O. Cools H. J. Foster S. J. Huang Y. J. (2007). Comparison of *Leptosphaeria biglobosa*-induced and chemically induced systemic resistance to *L. maculans* in *Brassica napus*. *Chin. Sci. Bull.* 52 1053–1062. 10.1007/s11434-007-0181-5

[B187] Lo Presti L. Lanver D. Schweizer G. Tanaka S. Liang L. Tollot M. (2015). Fungal effectors and plant susceptibility. *Ann. Rev. Plant Biol.* 66 513–545. 10.1146/annurev-arplant-043014-114623 25923844

[B188] Loon L. C. Rep M. Pieterse C. M. J. (2006). Significance of inducible defense-related proteins in infected plants. *Annu. Rev. Phytopathol.* 44 135–162. 10.1146/annurev.phyto.44.070505.143425 16602946

[B189] Lucas J. A. Crute I. R. Sherriff’t C. Gordon P. L. (1988). The identification of a gene for race-specific resistance to *Peronospora parasitica* (downy mildew) in *Brassica napus* var. *oleifera* (oilseed rape). *Plant Pathot.* 37 538–545. 10.1111/j.1365-3059.1988.tb02112.x

[B190] Lysak M. A. Koch M. A. Pecinka A. Schubert I. (2005). Chromosome triplication found across the tribe *Brassiceae*. *Genome Res.* 15 516–525. 10.1101/gr.3531105 15781573 PMC1074366

[B191] Ma L. Borhan M. H. (2015). The receptor-like kinase SOBIR1 interacts with *Brassica* napus LepR3 and is required for *Leptosphaeria maculans* AvrLm1-triggered immunity. *Front. Plant Sci.* 6:933. 10.3389/fpls.2015.00933 26579176 PMC4625043

[B192] Mamgain A. Biswas M. K. Dey N. (2019). Potential role of chemical elicitors in induced systemic resistance for the effective management of *Alternaria* blight in mustard. *J. Pharmacogn. Phytochem.* 8 2246–2250.

[B193] Mandal A. K. Dubey S. C. (2012). Genetic diversity analysis of *Sclerotinia sclerotiorum* causing stem rot in chickpea using RAPD, ITS-RFLP, ITS sequencing and mycelial compatibility grouping. *World J. Microbiol. Biotechnol.* 28 1849–1855. 10.1007/s11274-011-0981-2 22805971

[B194] Marcroft S. Wratten N. Purwantara A. Salisbury P. A. Potter T. D. Barbetti M. J. (2002). Reaction of a range of *Brassica* species under Australian conditions to the fungus, *Leptosphaeria maculans*, the causal agent of blackleg. *Austr. J. Exper. Agric.* 42 587–594. 10.1071/ea01112

[B195] Massand P. P. Yadava S. K. Sharma P. Kaur A. Kumar A. Arumugam N. (2010). Molecular mapping reveals two independent loci conferring resistance to *Albugo candida* in the east European germplasm of oilseed mustard *Brassica juncea*. *Theor. Appl. Genet.* 121 137–145. 10.1007/s00122-010-1297-6 20213517

[B196] Matheron M. Porchas M. (2005). Influence of soil temperature and moisture on eruptive germination and viability of sclerotia of *Sclerotinia minor* and *S. sclerotiorum*. *Plant Dis.* 89 50–54. 10.1094/pd-89-0050 30795284

[B197] Mayerhofer R. Wilde K. Mayerhofer M. Lydiate D. Bansal V. K. Good A. G. (2005). Complexities of chromosome landing in a highly duplicated genome: toward map-based cloning of a gene controlling blackleg resistance in *Brassica napus*. *Genetics* 171 1977–1988. 10.1534/genetics.105.049098 16143600 PMC1456120

[B198] Mazumder M. Das S. Saha U. Chatterjee M. Bannerjee K. Basu D. (2013). Salicylic acid-mediated establishment of the compatibility between *Alternaria brassicicola* and *Brassica juncea* is mitigated by abscisic acid in *Sinapis alba*. *Plant Physiol. Biochem.* 70 43–51. 10.1016/j.plaphy.2013.04.025 23770593

[B199] McDonald B. A. Linde C. (2002). Pathogen population genetics, evolutionary potential and durable resistance. *Annu. Rev. Phytopathol.* 40 349–379. 10.1146/annurev.phyto.40.120501.101443 12147764

[B200] McDonald M. R. Boland G. J. (2004). Forecasting diseases caused by *Sclerotinia* spp. in eastern Canada: fact or fiction? *Can. J. Plant Pathol.* 26 480–488. 10.1080/07060660409507168

[B201] McDowell J. M. Dhandaydham M. Long T. A. Aarts M. G. Goff S. Li S. (1998). Intragenic recombination and diversifying selection contribute to the evolution of downy mildew resistance at the RPP8 locus of *Arabidopsis*. *Plant Cell* 10 1861–1874. 10.2307/3870909 9811794 PMC143965

[B202] McDowell J. M. Williams S. G. Funderburg N. T. Eulgem T. Dangl J. L. (2005). Genetic analysis of developmentally regulated resistance to downy mildew (*Hyaloperonospora parasitica*) in *Arabidopsis thaliana*. *Mol. Plant Microb. Interact.* 18 1226–1234. 10.1094/MPMI-18-1226 16353557

[B203] Meena P. D. Awasthi R. P. Chattopadhyay C. Kolte S. J. Kumar A. (2010). *Alternaria* blight: a chronic disease in rapeseed-mustard. *J. Oilseed Brassica* 1 1–11. 10.5455/faa.120733

[B204] Meena P. D. Jambhulkar S. J. Gupta R. Meena H. S. Singh D. (2016). Rapid screening technique for *alternaria* blight resistance in indian mustard (*Brassica juncea* L.) using cotyledonary leaf method. *J. Plant Pathol.* 98 463–469.

[B205] Meena P. D. Thomas L. Singh D. (2014). Assessment of yield losses in *Brassica juncea* due to downy mildew (*Hyaloperonospora brassicae*). *J. Oilseed Brassica* 5 73–77.

[B206] Mehta N. Sangwan M. S. Srivastava M. P. Kumar R. (2002). Survival of *Alternaria brassicae* causing *Alternaria* blight in rapeseed-mustard. *J. Mycol. Plant Pathol.* 32 64–67.

[B207] Mei J. Ding Y. Lu K. Wei D. Liu Y. Onwusemu J. (2013). Identification of genomic regions involved in resistance against *Sclerotinia sclerotiorum* from wild *Brassica oleracea*. *Theor. Appl. Genet.* 126 549–556. 10.1007/s00122-012-2000-x 23096003

[B208] Mei J. Shao C. Yang R. Feng Y. Gao Y. Ding Y. (2020). Introgression and pyramiding of genetic loci from wild *Brassica oleracea* into *B. napus* for improving *Sclerotinia* resistance of rapeseed. *Theor. Appl. Genet.* 133 1313–1319. 10.1007/s00122-020-03552-w 32008057

[B209] Mei J. Wei D. Disi J. O. Ding Y. Liu Y. Qian W. (2012). Screening resistance against *Sclerotinia sclerotiorum* in *Brassica* crops with use of detached stem assay under controlled environment. *Eur. J. Plant Pathol.* 134 599–604. 10.1007/s10658-012-0040-3

[B210] Meyers B. C. Kozik A. Griego A. Kuang H. Michelmore R. W. (2003). Genome-Wide Analysis of NBS-LRR-Encoding Genesin *Arabidopsis*. *Plant Cell* 15 809–834. 10.1105/tpc.009308 12671079 PMC152331

[B211] Mohammed A. E. You M. P. Banga S. S. Barbetti M. J. (2018). Resistances to downy mildew (*Hyaloperonospora brassicae*) in diverse *Brassicaceae* offer new disease management opportunities for oilseed and vegetable crucifer industries. *Eur. J. Plant Pathol.* 153 915–929. 10.1007/s10658-018-01609-7

[B212] Mohanty A. Chrungu B. Verma N. Shivanna K. R. (2009). Broadening the genetic base of crop *Brassicas* by production of new intergeneric hybrid. *Czech. J. Genet. Plant Breed.* 45 117–122. 10.17221/35/2009-CJGPB

[B213] Mondal K. K. Bhattacharya R. C. Koundal K. R. Chatterjee S. C. (2007). Transgenic Indian mustard (*Brassica juncea*) expressing tomato glucanase leads to arrested growth of *Alternaria brassicae*. *Plant Cell Rep.* 26 247–252. 10.1007/s00299-006-0241-3 17016733

[B214] Mondal K. K. Chatterjee S. C. Viswakarma N. Bhattacharya R. C. Grover A. (2003). Chitinase-mediated inhibitory activity of *Brassica* transgenic on growth of *Alternaria brassicae*. *Curr. Microbiol.* 47 171–173. 10.1007/s00284-002-3980-6 14570264

[B215] Monot C. Pajot E. Corre D. L. Silue D. (2002). Induction of systemic resistance in broccoli (*Brassica oleracea* var. *botrytis*) against downy mildew (*Peronospora parasitica*) by avirulent isolates. *Biol. Control.* 24 75–81. 10.1016/s1049-9644(02)00006-3

[B216] Monteiro A. A. Coelho P. S. Bahcevandziev K. Valerio L. (2005). Inheritance of downy mildew resistance at cotyledon and adult-plant stages in ‘Couve Algarvia’ (*Brassica oleracea* var. *tronchuda*). *Euphytica* 141 85–92. 10.1007/s10681-005-5696-8

[B217] Morrall R. A. A. Dueck J. (1982). Epidemiology of *Sclerotinia* stem rot of rapeseed in Saskatchewan. *Can. Plant Pathol.* 4 161–168. 10.1080/07060668209501319

[B218] Naher N. Shamsi S. Ali M. R. Bashar M. A. (2018). Screening of *Sclerotinia* stem rot resistance in Bangladesh mustard germplasm using cotyledon assay method. *Dhaka Univ. J. Biol. Sci.* 27 85–92. 10.3329/dujbs.v27i1.46414

[B219] Nanjundan J. Manjunatha C. Radhamani J. Thakur A. K. (2020). Identification of new source of resistance to powdery mildew of Indian mustard and studying its inheritance. *Plant Pathol. J.* 36 111–120. 10.5423/PPJ.OA.07.2019.0205 32296291 PMC7143518

[B220] Narayani M. Srivastava S. (2018). Elicitation: a stimulation of stress in in vitro plant cell/tissue cultures for enhancement of secondary metabolite production. *Phytochem. Rev.* 16 1227–1252. 10.1007/s11101-017-9534-0

[B221] Narusaka M. Hatakeyama K. Shirasu K. Narusaka Y. (2014). *Arabidopsis* dual resistance proteins, both RPS4 and RRS1, are required for resistance to bacterial wilt in transgenic *Brassica* crops. *Plant Signal. Behav.* 9:e29130. 10.4161/psb.29130 25763492 PMC4203570

[B222] Nashaat N. I. Awasthi R. P. (1995). Evidence for differential resistance to *Peronospora parasitica* (downy mildew) in accessions of *Brassica juncea* (mustard) at the cotyledon stage. *J. Phytopathol.* 143 157–159. 10.1111/j.1439-0434.1995.tb00250.x

[B223] Nashaat N. I. Heran A. Awasthi R. P. Kolte S. J. (2004). Differential response and genes for resistance to *Peronospora parasitica* (downy mildew) in *Brassica juncea* (mustard). *Plant Breed.* 123 512–515. 10.1111/j.1439-0523.2004.01037.x

[B224] Nashaat N. I. Heran A. Mitchell S. E. Awasthi R. P. (1997). New genes for resistnace to downy mildew (*Peronospora parasitica*) in oilseed rape (*Brassica napus* ssp. *oleifrea*). *Plant Pathol.* 46 964–968. 10.1046/j.1365-3059.1997.d01-76.x

[B225] Nayanakantha N. M. C. Rawat S. Ali S. Grover A. (2016). Differential expression of defense-related genes in *Sinapis alba* and *Brassica juncea* upon the infection of *Alternaria brassicae*. *Trop. Agric. Res.* 27 123–136. 10.4038/tar.v27i2.8161

[B226] Neerja S. Sohal B. S. (2012). Induced resistance in *Brassica juncea* L. against *Alternaria* blight by foliar application of arachidonic acid. *Plant Dis. Res.* 27 153–157.

[B227] Nees Von Esenbeck G. G. (1817). *System der Plize Urid Schwamme.* Wurzburg: Arkose Press, 234.

[B228] Neik T. X. Barbetti M. J. Batley J. (2017). Current status and challenges in identifying disease resistance genes in *Brassica napus*. *Front. Plant Sci.* 8:1788. 10.3389/fpls.2017.01788 29163558 PMC5681527

[B229] Neukermans J. Inzé A. Mathys J. Coninck B. D. Cotte B. V. Bruno P. A. (2015). ARACINs, *Brassicaceae*-specific peptides exhibiting antifungal activities against necrotrophic pathogens in *Arabidopsis*. *Plant Physiol.* 167 1017–1029. 10.1104/pp.114.255505 25593351 PMC4348783

[B230] Nishimura S. Kohmoto K. (1983). Host-specific toxins and chemical struc-tures from *Alternaria* species. *Annu. Rev. Phytopathol.* 21 87–116. 10.1146/annurev.py.21.090183.000511 25946338

[B231] Nourani S. L. Minassian V. Safaie N. (2008). Identification, pathogenicity and distribution of *Alternaria* spp. of canola in Iran. *Iran. J. Plant Path.* 44 33–36.

[B232] Nowicki M. Nowakowska M. Niezgoda A. Kozik E. U. (2012). *Alternaria* black spot of crucifers: symptoms, importance of disease, and perspectives of resistance breeding. *Veget. Crops Res. Bull.* 76 5–19. 10.2478/v10032-012-0001-6

[B233] Osbourn A. E. (1996). Preformed antimicrobial compounds and plant defense against fungal attack. *Plant Cell* 8 1821–1831. 10.1105/tpc.8.10.1821 12239364 PMC161317

[B234] Pan Y. Wei J. Yao C. Reng H. Gao Z. (2018). SsSm1, a Cerato-platanin family protein, is involved in the hyphal development and pathogenic process of *Sclerotinia sclerotiorum*. *Plant Sci.* 270 37–46. 10.1016/j.plantsci.2018.02.001 29576085

[B235] Parada R. Y. Sakuna E. Mori N. Oka K. Egusa M. Kodoma M. (2008). *Alternaria brassicae* produces a host-specific protein toxin from germinating spores on host leaves. *Phytopathology* 98 458–463. 10.1094/PHYTO-98-4-0458 18944195

[B236] Paritosh K. Yadava S. K. Singh P. Bhayana L. Mukhopadhyay A. Gupta V. (2020). A chromosome-scale assembly of allotetraploid *Brassica juncea* (AABB) elucidates comparative architecture of the A and B genomes. *Plant Biotechnol. J.* 10.1111/pbi.13492 33073461 PMC7955877

[B237] Park Y. S. Jeon M. H. Lee S. H. Moon J. S. Cha J. S. Kim H. Y. (2005). Activation of defense responses in chinese cabbage by a nonhost pathogen, *Pseudomonas syringae* pv. tomato. *J. Biochem. Mol. Biol.* 38 748–754. 10.5483/bmbrep.2005.38.6.748 16336791

[B238] Parker J. E. Coleman M. J. Szabò V. Frost L. N. Schmidt R. Wang L. (1997). The *Arabidopsis* downy mildew resistance gene RPP5 shares similarity to the toll and interleukin-1 receptors with N and L6. *Plant Cell* 9 879–894. 10.1105/tpc.9.6.879 9212464 PMC156965

[B239] Parker J. E. Holub E. B. Frost L. N. Falk A. Gunn N. D. Danielsa M. J. (1996). Characterization of *edsl*, a mutation in *Arabidopsis* suppressing resistance to *Peronospora parasíiíca* specified by several different *RPP* genes. *Plant Cell* 8 2033–2046. 8953768 10.1105/tpc.8.11.2033 PMC161332

[B240] Pedras M. C. Zaharia L. I. (2000). Sinalbins A and B, phytoalexins from *Sinapis alba*:elicitation, isolation, and synthesis. *Phytochemistry* 55 213–216. 10.1016/s0031-9422(00)00277-6 11142844

[B241] Pedras M. S. Smith K. C. (1997). Sinalexin, a phytoalexin from white mustard elicited by destruxin B and *Alternaria brassicae*. *Phytochemistry* 46 833–837. 10.1016/s0031-9422(97)00362-2

[B242] Peltier A. J. Bradley C. A. Chilvers M. I. Malvick D. K. Mueller D. S. Wise K. A. (2012). Biology, yield loss and control of *Sclerotinia* stem rot of soybean. *J. Integ. Pest Mngmt.* 3 1–7. 10.1603/IPM11033 33044624

[B243] Perumal S. Koh C. S. Jin L. Sankoff D. Robinson S. J. Ian L. (2020). A high-contiguity *Brassica nigra* genome localizes active centromeres and defines the ancestral *Brassica* genome. *Nat. Plants* 6 929–941. 10.1038/s41477-020-0735-y 32782408 PMC7419231

[B244] Perwaiz M. S. Moghal S. M. Kamal M. (1969). Studies on the chemical control of white rust and downy mildew of rape (*Sarsoon*). *West Pak. J. Agric. Res.* 7 71–75.

[B245] Petrie G. A. (1973). Diseases of *Brassica* species in Saskatchewan, 1970-72. I. Staghead and aster yellows. *Can. Plant Dis. Surv.* 53 19–25.

[B246] Petrie G. A. (1978). Occurrence of a highly virulent strain of blackleg (*Leptosphaeria maculans*) on rape in Saskatchewan (1975-77). *Can. Plant Dis. Surv.* 58 21–25.

[B247] Petrie G. A. (1986). Consequences of survival of *Leptosphaeria maculans* (blackleg) in canola stubble residue through an entire crop rotation sequence. *Can. J. Plant Pathol.* 8:353.

[B248] Petrie G. A. (1988). Races of *Albugo candida* (white rust and staghead) on cultivated Cruciferae in Saskatchewan. *Can. J. Plant Pathol.* 10 142–150. 10.1080/07060668809501746

[B249] Petrie G. A. (1995). Long-term survival and sporulation of *Leptosphaeria maculans* (blackleg) on naturally-infected rapeseed/canola stubble in Saskatchewan. *Can. Plant Dis. Surv.* 75 23–34. 10.1071/app9790023

[B250] Petrie G. A. Vanterpool T. C. (1974). Fungi associated with hypertrophies caused by infection of Cruciferae by *Albugo cruciferatum*. *Can. Plant Dis. Surv.* 54 37–42.

[B251] Pickar-Oliver A. Gersbach C. A. (2019). The next generation of CRISPR-Cas technologies and applications. *Nat. Rev. Mol. Cell. Biol.* 20 490–507. 10.1038/s41580-019-0131-5 31147612 PMC7079207

[B252] Plieske J. Struss D. (2001). STS markers linked to *Phoma* resistance genes of the *Brassica* B-genome revealed sequence homology between *Brassica nigra* and *Brassica napus*. *Theor. Appl. Genet.* 102 483–488. 10.1007/s001220051671

[B253] Pokotylo I. Janda M. Kalachova T. Zachowski A. Ruelland E. (2017). “Phosphoglycerolipid signaling in response to hormones under stress,” in *Mechanism of Plant Hormone Signaling Under Stress-II, Part II: Interaction of Other Components with Phytohormones*, ed. Pandey G. K. (Cham: Springer), 91–126. 10.1002/9781118889022.ch22

[B254] Poveda J. Hermosa R. Monte E. Nicolás C. (2019). The *Trichoderma harzianum* kelch protein ThKEL1 plays a key role in root colonization and the induction of systemic defense in *Brassicaceae* plants. *Front. Plant Sci.* 10:1478. 10.3389/fpls.2019.01478 31803213 PMC6873215

[B255] Prabhu K. V. Somers D. J. Rakow G. Gugel R. K. (1998). Molecular markers linked to white rust resistance in mustard *Brassica juncea*. *Theor. Appl. Genet.* 97 865–870. 10.1007/s001220050966

[B256] Pradhan A. K. Prakash S. Mukhopadhyay A. Pental D. (1992). Phylogeny of *Brassica* and allied genera based on the variation in chloroplast and mitochondrial DNA patterns. Molecular and taxo-nomic classifi cations are incongruous. *Theor. Appl. Genet.* 85 331–340. 10.1007/bf00222878 24197323

[B257] Prakash S. Bhat S. R. Quiros C. F. Kirti P. B. Chopra V. L. (2009). *Brassica* and its close allies: cytogenetics and evolution. *Plant Breed. Rev.* 31 21–187. 10.1002/9780470593783.ch2

[B258] Prakash S. Hinata K. (1980). Taxonomy, cytogenetics and origin of crop *Brassicas*, a review. *Operar. Bot.* 55 1–57. 10.1016/b978-1-891127-79-3.50001-9

[B259] Primard C. Vedel F. Mathieu C. Pelletier G. Chevre A. M. (1988). Interspecific somatic hybridization between *Brassica napus* and *Brassica hirta* (*Sinapis alba* L.). *Theory Appl. Genet.* 75 546–552. 10.1007/BF00289119

[B260] Purdy L. H. (1979). *Sclerotinia sclerotiorum*: history, diseases and symptomatology, host range, geographic distribution, and impact. *Phytopathology* 69 875–880. 10.1094/phyto-69-875

[B261] Purnamasari M. I. Erskine W. Croser J. S. You M. P. Barbetti M. J. (2019). Comparative reaction of *Camelina sativa* to *Sclerotinia sclerotiorum* and *Laptosphaeria maculans*. *Plant Dis.* 103 2884–2892. 10.1094/PDIS-03-19-0664-RE 31486740

[B262] Qasim M. U. Zhao Q. Shahid M. Samad R. A. Ahmar S. Wu J. (2020). Identification of QTLs containing resistance genes for *Sclerotinia* stem rot in *Brassica napus* using comparative transcriptomic studies. *Front. Plant Sci.* 11:776. 10.3389/fpls.2020.00776 32655594 PMC7325899

[B263] Rajarammohan S. Kumar A. Gupta V. Pental D. Pradhan A. K. Kaur J. (2017). Genetic architecture of resistance to *Alternaria brassicae* in *Arabidopsis thaliana*: QTL mapping reveals two major resistance-conferring loci. *Front. Plant Sci.* 8:260. 10.3389/fpls.2017.00260 28286515 PMC5323384

[B264] Rajarammohan S. Paritosh K. Pental D. Barbulescu D. M. Qiu Y. Liu S. (2019a). Comparative genomics of *Alternaria* species provides insights into the pathogenic lifestyle of *Alternaria brassicae* - a pathogen of the *Brassicaceae* family. *BMC Genom.* 20:1036. 10.1186/s12864-019-6414-6 31888481 PMC6937934

[B265] Rajarammohan S. Pental D. Kaur J. (2019b). Near-complete genome assembly of *Alternaria brassicae*-A necrotrophic pathogen of *Brassica* crops. *MPMI* 32 928–930. 10.1094/MPMI-03-19-0084-A 30920345

[B266] Raman H. McVittie B. Pirathiban R. Raman R. Zhang Y. Barbulescu D. M. (2020). Genome-wide association mapping identifies novel loci for quantitative resistance to blackleg disease in canola. *Front. Plant Sci.* 11:1184. 10.3389/fpls.2020.01184 PMC7432127 32849733

[B267] Raman H. Raman R. Diffey S. Qiu Y. McVittie B. Barbulescu D. M. (2018). Stable quantitative resistance loci to blackleg disease in canola (*Brassica napus* L.) over continents. *Front. Plant Sci.* 9:1622. 10.3389/fpls.2018.01622 30532758 PMC6265502

[B268] Raman R. Taylor B. Lindbeck K. Coombes N. Barbulescu D. Salisbury P. (2012a). Molecular mapping and validation of Rlm1 gene for resistance to *Leptosphaeria maculans* in canola (*Brassica napus* L.). *Crop Past. Sci.* 63 1007–1017. 10.1071/CP12255

[B269] Raman R. Taylor B. Marcroft S. Stiller J. Eckermann P. Coombes N. (2012b). Molecular mapping of qualitative and quantitative loci for resistance to *Leptosphaeria maculans* causing blackleg disease in canola (*Brassica napus* L.). *Theor. Appl. Genet.* 125 405–418. 10.1007/s00122-012-1842-6 22454144

[B270] Rana K. Atri C. Akhatar J. Kaur R. Goyal A. Singh M. P. (2019). Detection of first marker trait associations for resistance against *Sclerotinia sclerotiorum* in *Brassica juncea*-*Erucastrum cardaminoides* introgression lines. *Front. Plant Sci.* 10:1015. 10.3389/fpls.2019.01015 31447876 PMC6691357

[B271] Rana K. Atri C. Gupta M. Akhatar J. Sandhu P. Kumar N. (2017). Mapping resistance to sclerotina infestation in introgression lines of *Brassica juncea* carring genomic segments from wild *Brassicaceae B. fruticulosa*. *Sci. Rep.* 7:5904. 10.1038/s41598-017-05992-9 28724956 PMC5517529

[B272] Rayss T. (1938). Nouvelle contribution altetude de la mycofbre de Palestine. *Palestian J. Bot.* 1 143–160.

[B273] Rejeb I. B. Pastor V. Mauch-Mani B. (2014). Plant responses to simultaneous biotic and abiotic stress: molecular mechanisms. *Plants* 3 458–475. 10.3390/plants3040458 27135514 PMC4844285

[B274] Ren L. Xu L. Liu F. Chen K. Sun C. Li J. (2016). Host range of *Plasmodiophora brassicae* on cruciferous crops and weeds in China. *Plant Dis.* 100 933–939. 10.1094/PDIS-09-15-1082-RE 30686153

[B275] Rimmer S. R. Van-den Berg C. G. J. (1992). Resistance of oilseed *Brassica* spp. to blackleg caused by *Leptosphaeria maculans*. *Can. J. Plant Pathol.* 14 56–66.

[B276] Ripley V. Thorpe M. Iler S. Mizier K. Beversdorf W. D. (1992). Isozyme analysis as a tool for introgression of *Sinapis albagerm* plasm into *Brassica napus*. *Theor. Appl. Genet.* 84 403–410. 10.1007/BF00229500 24203201

[B277] Rouxel T. Balesdent M. H. (2005). The stem canker (blackleg) fungus, *Leptosphaeria maculans*, enters the genomic era. *Mol. Plant Pathol.* 6 225–241. 10.1111/j.1364-3703.2005.00282.x 20565653

[B278] Rouxel T. Penaud A. Pinochet X. Brun H. Gout L. Delourme R. (2003). A 10-year survey of populations of *Leptosphaeria maculans* in France indicates a rapid adaptation towards the rlm1 resistance gene of oilseed rape. *Eur. J. Plant Pathol.* 109 871–881.

[B279] Rustagi A. Kumar D. Shekhar S. Yusuf M. A. Misra S. Sarin N. B. (2014). Transgenic *Brassica juncea* plants expressing MsrA1, a synthetic cationic antimicrobial peptide, exhibit resistance to fungal phytopathogens. *Mol. Biotechnol.* 56 535–545. 10.1007/s12033-013-9727-8 24452332

[B280] Saal B. Brun H. Glais I. Struss D. (2004). Identification of a *Brassica juncea*-derived recessive gene conferring resistance to *Leptosphaeria maculans* in oilseed rape. *Plant Breed.* 123 505–511. 10.1111/j.1439-0523.2004.01052.x

[B281] Saal B. Struss D. (2005). RGA- and RAPD-derived SCAR markers for a *Brassica* B-genome introgression conferring resistance to blackleg in oilseed rape. *Theoret. Appl. Genet.* 111 281–290. 10.1007/s00122-005-2022-8 15887037

[B282] Saccardo P. A. (1886). “Hyphomyceteae,” in *Sylloge Fungorum Omninum Hucusque Cognitorum*, Vol. 4 (Pavia: BHL), 807.

[B283] Saharan G. S. Kadian A. K. (1983). Physiologic specialization in *Alternaria brassicae*. *Crucif. News Lett.* 8 32–33.

[B284] Saharan G. S. Mehta N. (2008). *Sclerotinia Diseases of Crop Plants: Biology, Ecology and Disease Management.* Netherlands: Springer, 485.

[B285] Saharan G. S. Verma P. R. (1992). *White Rust. A Review of Economically Important Species.* Ottawa, ON: International Development Research Centre, 65. IDRC-MR315e, IV.

[B286] Sangwan M. S. Mehta N. (2007). Pathogenic variability in isolates of *Alternaria brassicae* (Berk.) Sacc. from different agro-climatic zones of India. *Plant Dis. Res.* 22 101–107.

[B287] Savulescu O. (1946). Study of the species of *Cystopus* (Pers.) Lev. Bucharest, 1946. *Anal. Acad. Rous. Mem. Sect. Stimtiface. Soc.* 21:13.

[B288] Schardl C. L. Craven K. D. (2003). Interspecific hybridization in plant associated fungi and oomycetes: a review. *Mol. Ecol.* 12 2861–2873. 10.1046/j.1365-294x.2003.01965.x 14629368

[B289] Sexton A. C. Whitten A. R. Howlett B. J. (2006). Population structure of *Sclerotinia sclerotiorum* in an Australian canola field at flowering and stem-infection stages of the disease cycle. *Genome* 49 1408–1415. 10.1139/g06-101 17426756

[B290] Sharma G. Kumar V. D. Haque A. Bhat S. R. Prakash S. Chopra V. L. (2002). *Brassica* coenospecies: a rich reser-voir for genetic resistance to leaf spot caused by *Alternaria brassicae*. *Euphytica* 125 411–417. 10.1023/A:1016050631673

[B291] Sharma P. Deep S. Sharma M. Bhati D. S. (2013). Genetic variation of *Alternaria brassicae* (Berk.) Sacc., causal agent of dark leaf spot of cauliflower and mustard in India. *J. Gen. Plant Pathol.* 79 41–45. 10.1007/s10327-012-0417-3

[B292] Sharma T. R. Singh B. M. (1992). Transfer of resistance to *Alternaria brassicae* in *Brassica juncea* through interspecific hybridization among *Brassicas*. *J. Genet. Breed.* 46 373–378.

[B293] Sharma T. R. Tewari J. P. (1998). RAPD analysis of three *Alternaria* species pathogenic to crucifers. *Mycol. Res.* 102 807–814. 10.1017/S0953756297005479

[B294] Shoemaker R. A. Brun H. (2001). The teleomorph of the weakly aggressive segregate of *Leptosphaeria maculans*. *Can. J. Bot.* 79 412–419. 10.1139/cjb-79-4-412

[B295] Shrestha S. K. Munk L. Mathur S. B. (2005). Role of weather on *Alternaria* leaf blight disease and its effect on yield and yield component of mustard. *Nepal Agric. Res. J.* 6 62–72. 10.3126/narj.v6i0.3366

[B296] Shukla A. K. (2005). Estimation of yield losses to Indian mustard (*Brassica juncea*) due to *Sclerotinia* stem rot. *J. Phytol. Res.* 18 267–268.

[B297] Sinapidou E. Williams K. Nott L. Bahkt S. Tör M. (2004). Two TIR: NB:LRR genes are required to specify resistance to *Peronospora parasitica* isolate Cala2 in *Arabidopsis*. *Plant J.* 38 898–909. 15165183 10.1111/j.1365-313X.2004.02099.x

[B298] Singh B. K. Nandan D. Supriya A. Ram B. Kumar A. Singh T. (2015). Validation of molecular markers for marker-assisted pyramiding of white rust resistance loci in Indian mustard (*Brassica juncea* L.). *Can. J. Plant Sci.* 95 939–945. 10.4141/cjps-2014-215

[B299] Singh H. V. (2000). Biochemical basis of resistance in *Brassica* species against downy mildew and white rust of mustard. *Plant Dis. Res.* 15 75–77.

[B300] Singh R. Singh D. Barbetti M. J. Singh H. Caixia L. Sivasithamparam K. (2008). *Sclerotinia* rot tolerance in oilseed *Brassica*. *J. Oilseeds Res.* 25 223–225.

[B301] Singh S. Sharma S. R. Kalia P. Deshmukh R. Kumar V. Sharma P. (2012). Molecular mapping of the downy mildew resistance gene Ppa3 in cauliflower (*Brassica oleracea* var. *botrytis* L.). *J. Hortic. Sci. Biotechnol.* 87 137–143. 10.1080/14620316.2012.11512844

[B302] Singh U. S. Nashaat N. I. Doughty K. J. Awasthi R. P. (2002). Altered phenotypic response to *Peronospora parasitica* in *Brassica juncea* seedlings following prior inoculation with an avirulent or virulent isolate of *Albugo candida*. *Eur. J. Plant Pathol.* 108 555–564. 10.1023/A:1019937115378

[B303] Sirhindi G. Mushtaq R. Sharma P. Kaur H. Ahmad M. M. (2017). “Jasmonate signaling and stress management in plants,” in *Mechanism of Plant Hormone Signaling under Stress-II, Part I: Action of Phytohormones in Stress*, ed. Pandey G. K. (Hoboken, NY: Wiley), 43–171. 10.1002/9781118889022.ch7

[B304] Sjodin C. Glimelius K. (1988). Screening for resistance to blackleg *Phoma lingam* (Tode ex Fr.) Desm. within *Brassicaceae*. *J. Phytopathol.* 123 322–332. 10.1111/j.1439-0434.1988.tb04484.x

[B305] Sodelade M. Pedras C. Khallaf I. (2012). Molecular interactions of the phytotoxins destruxin B and sirodesmin PL with crucifers and cereals. Metabolism and elicitation of plant defenses. *Phytochemistry* 77 129–139. 10.1016/j.phytochem.2012.02.010 22414311

[B306] Somers D. J. Rakow G. Rimmer S. R. (2002). *Brassica napus* DNA markers linked to white rust resistance in *Brassica juncea*. *Theor. Appl. Genet.* 104 1121–1124. 10.1007/s00122-001-0812-1 12582621

[B307] Song J. Guan Z. Hu J. (2020). Eight high-quality genomes reveal pan-genome architecture and ecotype differentiation of *Brassica napus*. *Nat. Plants* 6 34–45. 10.1038/s41477-019-0577-7 31932676 PMC6965005

[B308] Sperschneider J. Dodds P. N. Gardiner D. M. Agarwal P. Howlett B. J. Li Y. (2015). Advances and challenges in computational prediction of effectors from plant pathogenic fungi. *PLoS Pathg.* 11:e1004806. 10.1371/journal.ppat.1004806 26020524 PMC4447458

[B309] Sprague S. J. Balesdent M. H. Brun H. Hayden H. L. Marcroft S. J. Pinochet X. (2006a). Major gene resistance in *Brassica napus* (oilseed rape) is overcome by changes in virulence of populations of *Leptosphaeria maculans* in France and Australia. *Eur. J. Plant Pathol.* 114 33–40. 10.1007/1-4020-4525-5_3

[B310] Sprague S. J. Marcroft S. J. Hayden H. L. Howlett B. J. (2006b). Major gene resistance to blackleg in *Brassica napus* overcome within three years of commercial production in southeastern Australia. *Plant Dis.* 90 190–198. 10.1094/pd-90-0190 30786411

[B311] Stergiopoulos I. de Wit P. J. G. M. (2009). Fungal effector proteins. *Ann. Rev. Phytopathol.* 47 233–263. 10.1146/annurev.phyto.112408.132637 19400631

[B312] Stotz H. U. Mitrousia G. K. de Wit P. J. G. M. Fitt B. D. L. (2014). Effector-triggered defence against apoplastic fungal pathogens. *Trends Plant Sci.* 19 491–500. 10.1016/j.tplants.2014.04.009 24856287 PMC4123193

[B313] Taj G. Agarwal P. Grant M. Kumar A. (2011). Co-expression and *in-silico* interaction studies for inter-linking the activation of MAPK3 and LOX genes during pathogenesis of *Alternaria brassicae* in *Brassica juncea*. *J. Oilseed Brassica* 2 13–20.

[B314] Taylor I. D. Conway S. Roberts S. J. Astley D. Vicente I. G. (2002). Sources and origin of resistance to *Xanthomonas campestris* pv. *campestris* in *Brassica* genomes. *Phytopathology* 92 105–111. 10.1094/PHYTO.2002.92.1.105 18944146

[B315] Terras F. R. G. Eggermont K. Kovaleva V. Raikhel N. V. Osborn R. W. Kester A. (1995). Small cysteine-rich antifungal proteins from radish: their role in host defense. *Plant Cell* 7 573–588. 10.1105/tpc.7.5.573 7780308 PMC160805

[B316] Terras F. R. G. Torrekens S. Leuven F. V. Osborn R. W. Vanderleyden J. Cammuea B. P. A. (1993). A new family of basic cysteine-rich plant antifungal proteins from *Brassicaceae* species. *FEBS Lett.* 316 233–240. 10.1016/0014-5793(93)81299-f 8422949

[B317] Tewari J. P. (1983). Cellular alterations in the blackspot of rapeseed caused by *Alternaria brassicae*. *Phytopathology* 73:831.

[B318] Tewari J. P. (1991a). Resistance to *Alternaria brassicae* in crucifers. *Bull. SROP* 14 154–161.

[B319] Tewari J. P. (1991b). Structural and biochemical bases of the black spot diseases of crucifers. *Adv. Struc. Biol*. 1 25–34.

[B320] Tewari J. P. Conn K. L. (1993). Reactions of some wild crucifers to *Alternaria brassicae*. *Bull. OILS SROP* 16 53–58.

[B321] Thakur M. Sohal B. S. Sandhu P. S. (2014). Impact of elicitor spray on *Alternaria* blight severity and yield of *Brassica juncea* and *Brassica napus* species. *J. Oilseed Brassica* 5 78–82.

[B322] Thomma B. P. H. J. Eggermont K. Broekaert W. F. Cammue B. P. A. (2000). Disease development of several fungi on *Arabidopsis* can bereduced by treatment with methyl jasmonate. *Plant Physiol. Biochem.* 38 421–427. 10.1016/s0981-9428(00)00756-7

[B323] Thomma B. P. H. J. Eggermont K. Penninckx I. A. M. A. Mauch-Mani B. Vogelsang R. Cammue B. P. A. (1998). Separate jasmonate-dependent and salicylate-dependent defense response pathways in *Arabidopsis* are essential for resistance to distinct microbial pathogens. *Proc. Natl. Acad. Sci. U.S.A.* 95 15107–15111. 10.1073/pnas.95.25.15107 9844023 PMC24583

[B324] Thomma B. P. H. J. Eggermont K. Tierens F. M. J. Broekaert W. F. (1999a). Requirement of functional EIN2 (ethylene insensitive 2) gene for efficient resistance of *Arabidopsis thaliana* to infection by *Botrytis cinerea*. *Plant Physiol.* 121 1093–1101. 10.1104/pp.121.4.1093 10594097 PMC59477

[B325] Thomma B. P. H. J. Nelissen I. Eggermont K. Broekaert W. F. (1999b). Deficiency in phytoalexin production causes enhanced susceptibility of *Arabidopsis* thaliana to the fungus *Alternaria brassicicola*. *Plant J.* 19 163–171. 10.1046/j.1365-313x.1999.00513.x 10476063

[B326] Thomma B. P. H. J. Penninckx I. A. M. A. Broekaert W. F. Cammue B. P. A. (2001). The complexity of disease signaling in *Arabidopsis*. *Curr. Opin. Immunol.* 13 63–68. 10.1016/s0952-7915(00)00183-7 11154919

[B327] Thukral S. K. Singh H. (1986). Inheritance of white rust resistance in *Brassica juncea*. *Plant Breed.* 97 75–77. 10.1111/j.1439-0523.1986.tb01304.x

[B328] Tiwari A. S. Petrie G. A. Downey R. K. (1988). Inheritance of resistance to *Albugo candida* race 2 in mustard [*Brassica juncea* (L.) Czem.]. *Can. J. Plant Sci.* 68 297–300. 10.4141/cjps88-039

[B329] Tollenaere R. Hayward A. Dalton-Morgan J. Campbell E. Lee J. R. M. Lorenc M. T. (2012). Identification and characterization of candidate Rlm4 blackleg resistance genes in *Brassica napus* using next-generation sequencing. *Plant Biotechnol. J.* 10 709–715. 10.1111/j.1467-7652.2012.00716.x 22726421

[B330] Tonguc M. Griffiths P. D. (2004). “Transfer of powdery mildew resistance from *Brassica carinata* to *B. oleracea*,” in *Proceeings of the 101st International conference of American Society of Horticulture Science*, Austin, TX.

[B331] Tosi L. Zezzerini A. (1985). *Septoria helianthi* Ell. & Kell. nuovo parassita del girasole in Italia. *Inform. Fitopatol.* 39 43–44.

[B332] Tripathi N. N. Kaushik C. D. (1984). Studies on the survival of *Alternaria brassicae* the causal organism of leaf spot of rapeseed and mustard. *Madras Agric. J.* 71 237–241.

[B333] Tylkowska K. Korbas M. Kurzawińska H. Rataj-Guranowska M. (2004). “Objawy chorób powodowanych przez grzyby rodzaju *Alternaria*,” in *Kompendium Symptomów Cho-rób Roślin Oraz Morfologii Ich Sprawców*, ed. Rataj-Guranowska M. (Poznań: Bogucki Wydawnictwo Nau-kowe), 3–11.

[B334] Uloth M. You M. P. Finnegan P. M. Banga S. S. Yi H. Barbetti M. J. (2014). Seedling resistance to *Sclerotinia sclerotiorum* as expressed across diverse cruciferous species. *Plant Dis*. 98 184–190. 10.1094/pdis-06-13-0612-re 30708771

[B335] Uloth M. B. You M. P. Finnegan P. M. Banga S. S. Banga S. K. Sandhu P. S. (2013). New sources of resistance to *Sclerotinia sclerotiorum* for crucifer crops. *Field Crops Res.* 154 40–52. 10.1016/j.fcr.2013.07.013

[B336] USDA (2020). *Foreign Agriculture Service. United States Department of Agriculture.* Available online at: https://www.fas.usda.gov/regions/india.

[B337] Van Der Biezen E. A. Freddie C. T. Kahn K. Parker J. E. Jones J. D. (2002). *Arabidopsis* RPP4 is a member of the RPP5 multigene family of TIR-NB-LRR genes and confers downy mildew resistance through multiple signalling components. *Plant J.* 29 439–451. 10.1046/j.0960-7412.2001.01229.x 11846877

[B338] Van-de Wouw A. Lowe R. G. T. Elliott C. E. Dubois D. J. Howlett B. J. (2014). An avirulence gene, *AvrLmJ1*, from the blackleg fungus, *Leptosphaeria maculans*, confers avirulence to *Brassica juncea* cultivars. *Mol. Plant Pathol.* 15 523–530. 10.1111/mpp.12105 24279453 PMC6638781

[B339] Van-de Wouw A. P. Howlett B. J. Idnurm A. (2017). Changes in allele frequencies of avirulence genes in the blackleg fungus, *Leptosphaeria maculans*, over two decades in Australia. *Crop Pasture Sci.* 69 20–29. 10.1071/cp16411

[B340] Varshney A. Mohapatra T. Sharma R. P. (2004). Development and validation of CAPS and AFLP markers for white rust resistance gene in *Brassica juncea*. *Theor. Appl. Genet.* 109 153–159. 10.1007/s00122-004-1607-y 14997300

[B341] Vellios E. Karkanis A. Bilalis D. (2017). Powdery mildew (*Erysiphe cruciferarum*) infection on camelina (*Camelina sativa*) under Mediterranean conditions and the role of wild mustard (*Sinapis arvensis*) as alternative host of this pathogen. *Emirat. J. Food Agric.* 29 639–642. 10.9755/ejfa.2017-02-493

[B342] Verma A. Singh Y. (2018). Inheritance of downy mildew resistance and its relationship with biochemical traits in cauliflower (*Brassica oleracea* L. var. *botrytis*). *Crop Protect.* 106 132–138. 10.1016/j.cropro.2017.12.024

[B343] Verma P. R. Saharan G. S. (1994). Monograph on *Alternaria* diseases of crucifers. *Agric. Can. Res. Stat.* 6:162.

[B344] Verma S. S. Yajima W. R. Rahman M. H. Shah S. Liu J. Ekramoddoullah A. K. M. (2012). A cysteine-rich antimicrobial peptide from *Pinus monticola* (PmAMP1) confers resistance to multiple fungal pathogens in canola (*Brassica napus*). *Plant Mol. Biol.* 79 61–74. 10.1007/s11103-012-9895-0 22351159

[B345] Verma V. D. Rai B. (1980). Note on induced mutagenesis for spotting out the sources of resistance to *Alternaria* leaf spot in Indian mustard. *Indian J. Agric. Sci.* 50 278–280.

[B346] Vicente J. G. Gunn N. D. Bailey L. Pink D. A. C. Holub E. B. (2012). Genetics of resistance to downy mildew in *Brassica oleracea* and breeding towards durable disease control for UK vegetable production. *Plant Pathol.* 61 600–609. 10.1111/j.1365-3059.2011.02539.x

[B347] Viegas A. P. Teixeira A. R. (1943). Alguns fungos do Brasil. *Bragantia Sao Paulo* 3 223–269. 10.1590/s0006-87051943000800003

[B348] Vincenot L. Balesdent M. Li H. Barbetti M. J. Sivasithamparam K. Gout L. (2008). Occurrence of a new subclade of *Leptosphaeria biglobosa* in Western Australia. *Phytopathology* 98 321–329. 10.1094/phyto-98-3-0321 18944083

[B349] Vishwanath Kolte S. J. (1997). Variability in *Alternaria brassicae*: Response to host genotypes, toxin production and fungicides. *Indian Phytopathol.* 50 373–381.

[B350] Vishwanath Kolte S. J. Singh M. P. Awasthi R. P. (1999). Induction of resistance in mustard (*Brassica juncea*) against *Alternaria* black spot with an avirulent *Alternaria brassicae* isolate-D. *Eur. J. Plant Pathol.* 105 217–220.

[B351] Vleeshouwers V. G. A. A. Rietman H. Krenek P. Champouret N. Young C. Oh S. K. (2008). Effector genomics accelerates discovery and functional profiling of potato disease resistance and *Phytophthora Infestans* avirulence genes. *PLoS One* 3:e2875. 10.1371/journal.pone.0002875 18682852 PMC2483939

[B352] Vogel J. Somerville S. (2000). Isolation and characterization of powdery mildew-resistant *Arabidopsis* mutants. *Proc. Natl. Acad. Sci. U.S.A.* 97 1897–1902. 10.1073/pnas.030531997 10677553 PMC26533

[B353] Volkan C. Freddy B. Wiebke A. Alexandre R.-S. Oliver J. F. Amey R. (2019). Transgressive segregation reveals mechanisms of Arabidopsis immunity to Brassica-infecting races of white rust (*Albugo candida*). *Proc. Natl. Acad. Sci.* 116 2767–2773. 10.1073/pnas.1812911116 30692254 PMC6377460

[B354] Waalen W. M. Tanino K. K. Olsen J. E. Eltun R. Rognli O. A. Gusta L. V. (2011). Freezing tolerance of winter canola cultivars is best revealed by a prolonged freeze test. *Crop Sci.* 51 1988–1996. 10.2135/cropsci2011.02.0098

[B355] Walker J. C. (1957). *Plant Pathology.* New York, NY: McGraw-Hill Book Co., Inc, 214–219.

[B356] Wang M. Farnham M. W. Thomas C. E. (2001). Inheritance of true leaf stage downy mildew resistance in broccoli. *J. Amer. Soc. Hort. Sci.* 126 727–729. 10.21273/jashs.126.6.727

[B357] Wang X. Wang H. Wang J. Sun R. Wu J. Liu S. (2011). *Brassica rapa* genome sequencing project consortium. The genome of the mesopolyploid crop species *Brassica rapa*. *Nat. Genet.* 43 1035–1039. 10.1038/ng.919 21873998

[B358] Wang Y. P. Sonntag K. Rudloff E. Chen J. (2005). Intergeneric somatic hybridization between *Brassica napus* L. and *Sinapis alba* L. *J. Integr. Plant Biol.* 47 84–91. 10.1111/j.1744-7909.2005.00009.x

[B359] Wang Z. Ma L. Y. Cao J. Li Y. L. Ding L. N. Zhu K. M. (2019). Recent Advances in Mechanisms of Plant Defense to *Sclerotinia sclerotiorum*. *Front. Plant Sci.* 10:1314. 10.3389/fpls.2019.01314 31681392 PMC6813280

[B360] Warwick S. I. (2011). “*Brassicaceae* in agriculture,” in *Genetics and Genomics of the Brassicaceae. Plant Genetics and Genomics: Crop Models 9*, eds Schmidt R. Bancroft I. (Berlin: Springer Science Business Media), 33–65. 10.1007/978-1-4419-7118-0_2

[B361] Warwick S. I. Black L. D. (1991). Molecular systematics of *Brassica* and allied genera (Subtribe *Brassicinae Brassicae*) chloroplast genome and cytodeme congruence. *Theor. Appl. Genet.* 82 81–92. 10.1007/bf00231281 24212864

[B362] Warwick S. I. Francis A. Al-Shehbaz I. A. (2006). *Brassicaceae*: species checklist and database on CD-Rom. *Plant Syst. Evol.* 259 249–258. 10.1007/s00606-006-0422-0

[B363] Wei D. Mei J. Fu Y. Disi J. O. Li J. Qian W. (2014). Quantitative trait loci analyses for resistance to *Sclerotinia sclerotiorum* and flowering time in *Brassica napus*. *Mol. Breed.* 34 1797–1804. 10.1007/s11032-014-0139-7

[B364] Wei Z. Wang M. Chang S. Wu C. Liu P. Meng J. (2016). Introgressing subgenome components from *Brassica rapa* and *B. carinata* to *B. juncea* for broadening its genetic base and exploring intersubgenomic heterosis. *Front. Plant Sci.* 7:1677. 10.3389/fpls.2016.01677 27909440 PMC5112257

[B365] Werner S. Diederichsen E. Frauen M. Schondelmaier J. Jung C. (2007). Genetic mapping of clubroot resistance genes in oilseed rape. *Theor. Appl. Genet.* 116 363–372. 10.1007/s00122-007-0674-2 18040658

[B366] West J. S. Kharbanda P. Barbetti M. J. Fitt B. D. L. (2001). Epidemiology and management of *Leptosphaeria maculans* (phoma stem canker) in Australia, Canada and Europe. *Plant Pathol.* 50 10–27. 10.1046/j.1365-3059.2001.00546.x

[B367] Westman A. L. Kresovich S. Dickson M. H. (1999). Regional variation in *Brassica nigra* and other weedy crucifers for disease reaction to *Alternaria brassicicola* and *Xanthomonas campestris* pv. *campestris*. *Euphytica* 106 253–259. 10.1023/A:1003544025146

[B368] Wight W. D. Kim K. H. Lawrence C. B. Walton J. D. (2009). Biosynthesis and role in virulence of the histone deacetylase inhibitor depudecin from *Alternaria brassicicola*. *MPMI* 22 1258–1267. 10.1094/MPMI-22-10-1258 19737099

[B369] Williams P. H. Saha L. R. (1993). *Diseases of Mustard (Brassica juncea).* Available online at: https://www.Apsnet.org/edcenter/resources/commonnames/Pages/Mustard.Aspx (accessed June 28, 2020).

[B370] Winter H. Diestel A. Gärtig S. Wu J. Chen P. Zhao Q. (2003). “Transfer of new blackleg resistances into oilseed rape,”in *Proceedings of GCIRC 11th International Rapeseed Congress*, Saskatoon, Vol. 1 19–21.

[B371] Winter M. Koopmann B. (2016). Race spectra of *Leptosphaeria maculans*, the causal agent of blackleg disease of oilseed rape, in different geographic regions in northern Germany. *Eur. J. Plant Pathol.* 145 629–641. 10.1007/s10658-016-0932-8

[B372] Wu J. Cai G. Q. Tu J. Y. Li L. X. Liu S. Luo X. P. (2013). Identification of QTLs for resistance to *Sclerotinia* stem rot and BnaC.IGMT5. A as a candidate gene of the major resistant QTL SRC6 in *Brassica napus*. *PLoS One* 8:e67740. 10.1371/journal.pone.0067740 23844081 PMC3699613

[B373] Wu J. Chen P. Zhao Q. Cai G. Hu Y. Xiang Y. (2019). Co-location of QTL for *Sclerotinia* stem rot resistance and flowering time in *Brassica napus*. *Crop J.* 7 227–237. 10.1016/j.cj.2018.12.007

[B374] Wu J. Zhao Q. Yang Q. Liu H. Li Q. Yi X. (2016). Comparative transcriptomic analysis uncovers the complex genetic network for resistance to *Sclerotinia sclerotiorum* in *Brassica napus*. *Sci. Rep.* 6:19007. 10.1038/srep19007 26743436 PMC4705546

[B375] Xiao S. Calis O. Patrick E. Zhang G. Charoenwattana P. Muskett P. (2005). The atypical resistance gene, RPW8, recruits components of basal defense for powdery mildew resistance in *Arabidopsis*. *Plant J.* 42 95–110. 10.1111/j.1365-313X.2005.02356.x 15773856

[B376] Xiao S. Charoenwattana P. Holcombe L. Turner J. G. (2003). The *Arabidopsis* genes *RPW8.1* and *RPW8.2* confer induced resistance to powdery mildew diseases in tobacco. *MPMI* 16 289–294. 10.1094/mpmi.2003.16.4.289 12744457

[B377] Xiao S. EIIwood S. Findlay K. Oliver R. P. Turner J. G. (1997). Characterization of three loci controlling resistance of *Arabidopsis thaliana* accession Ms-0 to two powdery mildew diseases. *Plant J.* 12 757–768. 10.1046/j.1365-313x.1997.12040757.x 9375391

[B378] Yang J. Liu D. Wang X. Ji C. Cheng F. Liu B. (2016). The genome sequence of allopolyploid *Brassica juncea* and analysis of differential homoeolog gene expression influencing selection. *Nat. Genet.* 48 1225–1232. 10.1038/ng.3657 27595476

[B379] Yang J. Liu D. Wang X. Ji C. Cheng F. Liu B. (2018). The genome sequence of allopolyploid *Brassica juncea* and analysis of differential homoeolog gene expression influencing selection. *Nat. Genet.* 50:1616. 10.1038/s41588-018-0227-4 30250127

[B380] Yin X. Yi B. Chen W. Liang X. Yu O. Zhang Y. (2010). Mapping of QTLs detected in a *Brassica napus* DH population for resistance to *Sclerotinia sclerotiorum* in multiple environments. *Euphytica* 173 25–35. 10.1007/s10681-009-0095-1

[B381] You M. P. Uloth M. B. Li X. X. Banga S. S. Banga S. K. Barbetti M. J. (2016). Valuable new resistances ensure improved management of *Sclerotinia* Stem Rot (*Sclerotinia sclerotiorum*) in horticultural and oilseed *Brassica* species. *J. Phytopathol.* 164 291–299. 10.1111/jph.12456

[B382] Yu F. Gugel R. K. Kutcher H. R. Peng G. Rimmer S. R. (2013). Identification and mapping of a novel blackleg resistance locus LepR4 in the progenies from *Brassica napus* × *B. rapa* subsp. *sylvestris*. *Theor. Appl. Genet.* 126 307–315. 10.1007/s00122-012-1919-2 22733446

[B383] Yu F. Lydiate D. Rimmer S. R. (2005). Identification of two novel genes for blackleg resistance in *Brassica napus*. *Theo. Appl. Genet.* 110 969–979. 10.1007/s00122-004-1919-y 15798929

[B384] Yu F. Lydiate D. Rimmer S. R. (2008). Identification and mapping of a third blackleg resistance locus in *Brassica napus* derived from *B. rapa* subsp. *sylvestris*. *Genome* 51 64–72. 10.1139/g07-103 18356940

[B385] Yu J. Tehrim S. Zhang F. Tong C. Huang J. Cheng X. (2014). Genome-wide comparative analysis of NBS-encoding genes between *Brassica* species and *Arabidopsis thaliana*. *BMC Genom.* 15:3. 10.1186/1471-2164-15-3 24383931 PMC4008172

[B386] Yu S. Su T. Zhi S. Zhang F. Wang W. Zhang D. (2016). Construction of a sequence-based bin map and mapping of QTLs for downy mildew resistance at four developmental stages in Chinese cabbage (*Brassica rapa* L. ssp. *pekinensis*). *Mol. Breed.* 36:44. 10.1007/s11032-016-0467-x

[B387] Yu S. Zhang F. Zhao X. Yu Y. Zhang D. (2011). Sequence-characterized amplified region and simple sequence repeat markers for identifying the major quantitative trait locus responsible for seedling resistance to downy mildew in Chinese cabbage (*Brassica rapa* ssp. *pekinensis*). *Plant Breed.* 130 580–583.

[B388] Zeilmaker T. Ludwig N. R. Elberse J. Seidl M. F. Berke L. Doorn A. V. (2015). DOWNY MILDEW RESISTANT 6 and DMR6-LIKE OXYGENASE 1 are partially redundant but distinct suppressors of immunity in *Arabidopsis*. *Plant J.* 81 210–222. 10.1111/tpj.12719 25376907

[B389] Zhang H. Wu Q. Cao S. Zhao T. Chen L. Zhuang P. (2014). A novel protein elicitor (SsCut) from *Sclerotinia sclerotiorum* induces multiple defense responses in plants. *Plant Mol. Biol.* 86 495–511. 25149470 10.1007/s11103-014-0244-3

[B390] Zhang L. Cai X. Wu J. Berke L. Doorn A. V. Cao S. (2018). Improved *Brassica rapa* reference genome by single-molecule sequencing and chromosome conformation capture technologies. *Hortic. Res.* 5:50. 10.1038/s41438-018-0071-9 30131865 PMC6092429

[B391] Zhang W. Fraiture M. Kolb D. Loffelhardt B. Desaki Y. Boutrot F. F. (2013). *Arabidopsis* receptor-like protein30 and receptor-like kinase suppressor of BIR1-1/EVERSHED mediate innate immunity to necrotrophic fungi. *Plant Cell* 25 4227–4241. 10.1105/tpc.113.117010 24104566 PMC3877809

[B392] Zhang X. Fernando W. G. D. (2018). Insights into fighting against blackleg disease of *Brassica* napus in Canada. *Crop Pasture Sci.* 69 40–47. 10.1071/cp16401

[B393] Zhang X. Peng G. Kutcher H. R. Balesdent M. H. Delourme R. Fernando W. G. D. (2016). Breakdown of *Rlm3* resistance in the *Brassica napus-Leptosphaeria maculans* pathosystem in western Canada. *Eur. J. Plant Pathol.* 145 659–674. 10.1007/s10658-015-0819-0

[B394] Zhang Z. Y. Wang Y. X. Liu Y. L. (1984). Taxonomic studies of the family Albuginaceae of China. II. A new species of *Albugo* on Acanthaceae and known species of *Albugo* on cruciferae. *Acta Mycol. Sin.* 3 65–71.

[B395] Zhao J. Meng J. (2003). Genetic analysis of loci associated with partial resistance to *Sclerotinia sclerotiorum* in rapeseed (*Brassica napus* L.). *Theor. Appl. Genet.* 106 759–764. 10.1007/s00122-002-1171-2 12596007

[B396] Zhao J. Udall J. A. Quijada P. A. Grau C. R. Meng J. Osborn T. C. (2006). Quantitative trait loci for resistance to *Sclerotinia sclerotiorum* and its association with a homeologous non-reciprocal transposition in *Brassica napus* L. *Theor. Appl. Genet.* 112 509–516. 10.1007/s00122-005-0154-5 16333614

[B397] Zheng L. Zhao J. Liang X. Zhan G. Jiang S. Kang Z. (2017). Identification of a novel *Alternaria alternata* strain able to hyperparasitize *Puccinia striiformis* f. sp. *tritici*, the causal agent of wheat stripe rust. *Front. Microbiol.* 8:71. 10.3389/fmicb.2017.00071 28197134 PMC5281574

[B398] Zhu J. Spanier A. (1991). Resistance sources to *Phoma lingam* and *Alternaria brassicae*. *Eucarpia Crucif. Newsl*. 14:143.

